# Loss of Bacitracin Resistance Due to a Large Genomic Deletion among *Bacillus anthracis* Strains

**DOI:** 10.1128/mSystems.00182-18

**Published:** 2018-10-30

**Authors:** Yoshikazu Furuta, Hayato Harima, Emiko Ito, Fumito Maruyama, Naomi Ohnishi, Ken Osaki, Hirohito Ogawa, David Squarre, Bernard Mudenda Hang'ombe, Hideaki Higashi

**Affiliations:** a Division of Infection and Immunity, Research Center for Zoonosis Control, Hokkaido University, Sapporo, Japan; b Hokudai Center for Zoonosis Control in Zambia, Research Center for Zoonosis Control, Hokkaido University, Sapporo, Japan; c Department of Microbiology, Graduate School of Medicine, Kyoto University, Kyoto, Japan; d TOMY Digital Biology Co., Ltd., Tokyo, Japan; e Department of National Parks and Wildlife, Chilanga, Zambia; f Department of Para-clinical Studies, School of Veterinary Medicine, University of Zambia, Lusaka, Zambia; Agricultural Biotechnology Research Center

**Keywords:** *Bacillus anthracis*, *Bacillus cereus* group, antibiotic resistance, bacitracin, genome analysis, rRNA operon, unequal crossing over

## Abstract

Bacillus anthracis is a Gram-positive endospore-forming bacterial species that causes anthrax in both humans and animals. In Zambia, anthrax cases are frequently reported in both livestock and wildlife, with occasional transmission to humans, causing serious public health problems in the country. To understand the genetic diversity of B. anthracis strains in Zambia, we sequenced and compared the genomic DNA of B. anthracis strains isolated across the country. Single nucleotide polymorphisms clustered these strains into three groups. Genome sequence comparisons revealed a large deletion in strains belonging to one of the groups, possibly due to unequal crossing over between a pair of rRNA operons. The deleted genomic region included genes conferring resistance to bacitracin, and the strains with the deletion were confirmed with loss of bacitracin resistance. Similar deletions between rRNA operons were also observed in a few B. anthracis strains phylogenetically distant from Zambian strains. The structure of bacitracin resistance genes flanked by rRNA operons was conserved only in members of the Bacillus cereus group. The diversity and genomic characteristics of B. anthracis strains determined in this study would help in the development of genetic markers and treatment of anthrax in Zambia.

**IMPORTANCE** Anthrax is caused by Bacillus anthracis, an endospore-forming soil bacterium. The genetic diversity of B. anthracis is known to be low compared with that of Bacillus species. In this study, we performed whole-genome sequencing of Zambian isolates of B. anthracis to understand the genetic diversity between closely related strains. Comparison of genomic sequences revealed that closely related strains were separated into three groups based on single nucleotide polymorphisms distributed throughout the genome. A large genomic deletion was detected in the region containing a bacitracin resistance gene cluster flanked by rRNA operons, resulting in the loss of bacitracin resistance. The structure of the deleted region, which was also conserved among species of the Bacillus cereus group, has the potential for both deletion and amplification and thus might be enabling the species to flexibly control the level of bacitracin resistance for adaptive evolution.

## Abstract (precis)

Anthrax is caused by Bacillus anthracis, an endospore-forming soil bacterium. The genetic diversity of B. anthracis is known to be low compared with that of Bacillus species. In this study, we performed whole-genome sequencing of Zambian isolates of B. anthracis to understand the genetic diversity between closely related strains. Comparison of genomic sequences revealed that closely related strains were separated into three groups based on single nucleotide polymorphisms distributed throughout the genome. A large genomic deletion was detected in the region containing a bacitracin resistance gene cluster flanked by rRNA operons, resulting in the loss of bacitracin resistance. The structure of the deleted region, which was also conserved among species of the Bacillus cereus group, has the potential for both deletion and amplification and thus might be enabling the species to flexibly control the level of bacitracin resistance for adaptive evolution.

## INTRODUCTION

Bacillus anthracis is an endospore-forming Gram-positive bacterium that causes anthrax, a worldwide zoonotic disease (1). Among animals, the bacterium mainly infects herbivores and causes anthrax in livestock such as cattle, sheep, and goats, as well as in wildlife such as kudus, elephants, and hippopotamuses (2). Contact with infected animals or materials contaminated with B. anthracis results in the transmission of anthrax to humans. Two plasmids of B. anthracis, pXO1 and pXO2, carry genes producing virulence factors and capsule synthetases, respectively (3). The pXO1 plasmid harbors genes encoding a protective antigen, lethal factor, and edema factor, all of which contribute to virulence (4), whereas pXO2 harbors an operon encoding proteins for synthesis of a poly-γ-d-glutamate capsule that increases the stability of cells in a natural environment (3).

In Zambia, anthrax cases are observed every year, and outbreaks occur once every few years. Anthrax cases occur in both livestock in communities and wildlife in national parks, with occasional cases of infection among humans (5, 6). Most recent cases of human infection and animal infection were observed in an outbreak in the eastern part of Zambia in 2011, with more than 500 cases of human infection and five deaths, following the occurrence of an anthrax outbreak among hippopotamuses (7).

Several genetic markers have been used for the phylogenetic analysis of B. anthracis strains (8), including amplified fragment length polymorphisms (AFLPs) (9), multilocus variable-number tandem repeats (10, 11), single nucleotide polymorphisms (SNPs) (12), canonical SNPs (13, 14), and whole-genome sequences (15), although the species is known to have low genetic diversity (16, 17). Recent analysis using high-throughput short read sequencers enabled comparison of genome-wide polymorphisms and revealed phylogenetic clusters and the distribution and migration history of species at a higher resolution (18, 19). The results of this whole-genome analysis are consistent with phylogenetic groups, previously defined as A, B, and C, and additionally revealed subgroups and diversity within these phylogenetic groups (20–22).

To assess the genetic diversity of B. anthracis strains in Zambia in detail, we performed whole-genome sequencing of B. anthracis strains collected from Zambia, including isolates from recent cases of anthrax in animals and humans. Phylogenetic analysis of these strains revealed three groups among closely related strains based on genome-wide SNPs. Despite the characteristics of few genome rearrangements in the species (23), a large genomic deletion within a pair of rRNA operons was detected among strains belonging to one of the three groups, resulting in the loss of bacitracin resistance. A similar genomic deletion was also observed in other B. anthracis strains, as well as in species of the Bacillus cereus group, suggesting that such a deletion may contribute to the flexible control of the level of bacitracin resistance and to the adaptive evolution of B. anthracis strains.

## RESULTS

### SNPs and a large genomic deletion in Zambian *B. anthracis* strains.

**FIG 1:**
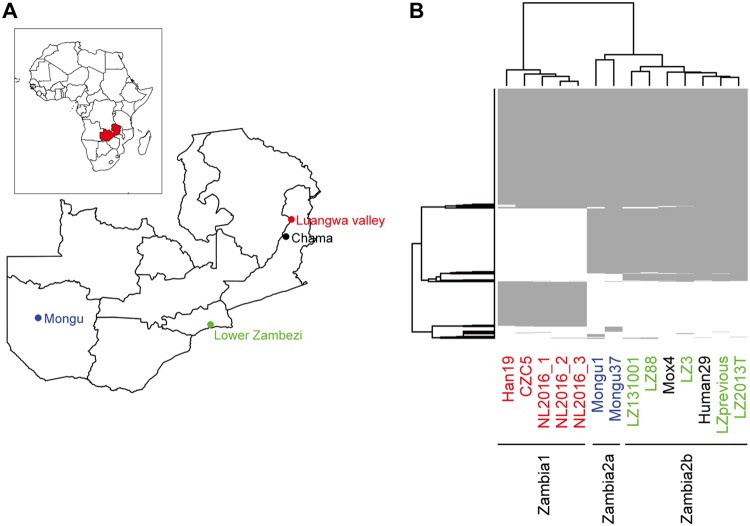
Single nucleotide polymorphisms (SNPs) in B. anthracis strains isolated in Zambia. (A) Map of Zambia and sampling locations of B. anthracis strains. (B) Heat map of SNPs in each B. anthracis strain isolated in Zambia. Gray indicates the presence of SNPs. The genome sequence of the B. anthracis Ames ancestor strain was used as reference. The color of strains corresponds to the color of the sampling locations in panel A.

To elucidate the diversity in B. anthracis strains in Zambia, genomic DNA of 14 Zambian strains sampled from various locations and sources and over several years (Fig. 1A and Table 1) was sequenced using the Illumina MiSeq platform. Sequence reads from all 14 strains were mapped on the reference genome sequence of the B. anthracis Ames ancestor to detect SNPs. A total of only 883 SNPs were identified, which is consistent with the low genetic diversity of B. anthracis species (see Table S1 in the supplemental material). Of the 883 SNPs, 409 were detected in all 14 Zambian strains. The remaining 474 core SNPs were therefore used for phylogenetic analysis of the Zambian strains. The Zambian strains were clustered into three groups showing some relationship with the sampling locations; these three groups were named Zambia1, Zambia2a, and Zambia2b (Fig. 1B).

**TABLE 1:** *Bacillus anthracis* strains isolated in Zambia

Strain	Location^*a*^	Source	Yr	Read depth	Accession no.	Group
NL2016_1	L.V.	Soil	2016	723	DRR118317	Zambia1
NL2016_2	L.V.	Soil	2016	848	DRR118318	Zambia1
NL2016_3	L.V.	Soil	2016	695	DRR118319	Zambia1
CZC5	L.V.	Hippopotamus	2011	171	DRR014735	Zambia1
Han19	L.V.	Soil	2012	50	DRR125654	Zambia1
Mongu1	Mongu	Cattle	2012	69	DRR125653	Zambia2a
Mongu37	Mongu	Cattle	2013	161	DRR014741	Zambia2a
Human29	Chama	Human	2013	207	DRR014739	Zambia2b
Mox4	Chama	Hippopotamus	2013	138	DRR014740	Zambia2b
LZprevious	L.Z.	Elephant	2011	161	DRR014736	Zambia2b
LZ2013T	L.Z.	Elephant	2011	98	DRR014737	Zambia2b
LZ88	L.Z.	Elephant	2013	70	DRR125657	Zambia2b
LZ3	L.Z.	Elephant	2013	59	DRR125655	Zambia2b
LZ131001	L.Z.	Elephant	2013	65	DRR125656	Zambia2b

a L.V., Luangwa Valley; L.Z., Lower Zambezi.

10.1128/mSystems.00182-18.7 TABLE S1 SNPs in B. anthracis Zambian isolates. Download Table S1, PDF file, 0.2 MB. Copyright © 2018 Furuta et al. 2018 Furuta et al. This content is distributed under the terms of the Creative Commons Attribution 4.0 International license.

**FIG 2:**
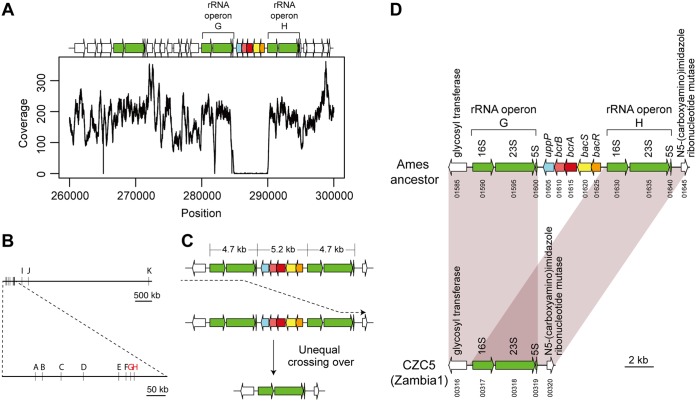
Large genomic deletion between rRNA operons in Zambia1 B. anthracis strains. (A) Sequence coverage of short reads of the CZC5 strain mapped to the reference genome sequence of the Ames ancestor. Arrows above the chart indicate open reading frames in the corresponding genomic regions of the Ames ancestor chromosome from bp 260000 to 300000. Arrows of different colors indicate protein coding regions (with the protein product in parentheses) or rRNA as follows: green, rRNA operons; blue, *uppP* (undecaprenyl-diphosphatase); pink, *bcrB* (ABC transporter permease); red, *bcrA* (ABC transporter ATP-binding protein); yellow, *bacS* (sensor histidine kinase); orange, *bacR* (DNA-binding response regulator); white, other genes. (B) Positions of 11 rRNA operons in the Ames ancestor strain. Genomic DNA is shown in linear form. The bottom panel magnifies the genomic region encompassing the rRNA operons A to H. rRNA operons G and H, which flank the deleted region, are indicated in red. (C) Schematic representation of the deletion via unequal crossing over between rRNA operons present as 4.7-kb-long direct repeats. (D) Deletion of the genomic region flanked by rRNA operons G and H. Genes are indicated by the number of the locus tag that in the full locus tag designation follows the prefix “GBAA_RS” for the Ames ancestor or “BAZ_” for CZC5.

In addition to SNPs, other genomic characteristics differentiating the Zambian strains were searched across the whole-genome sequences, and a large genomic deletion was identified specifically among the Zambia1 strains. Mapping of short reads of Zambian strains on the genome sequence of the B. anthracis Ames ancestor revealed a stretch of approximately 5 kb of unmapped genomic region specifically among the Zambia1 strains (Fig. 2A; see Fig. S1 in the supplemental material). Deletion of this region in Zambia1 strains was further confirmed by sequencing one of the Zambia1 strains, CZC5, using the PacBio RS platform, which generated long reads spanning the deleted region (see Fig. S2 in the supplemental material).

10.1128/mSystems.00182-18.1 FIG S1 Sequence coverage of short reads of Zambian strains mapped to the reference genome sequence of the Ames ancestor strain. (A) Zambia1 strains. (B) Zambia2a strains. (C) Zambia2b strains. Arrows of different colors indicate protein coding regions (with protein products in parentheses) or rRNA as follows: green, rRNA operons; blue, *uppP* (undecaprenyl-diphosphatase); pink, *bcrB* (ABC transporter permease); red, *bcrA* (ABC transporter ATP-binding protein); yellow, *bacS* (sensor histidine kinase); orange, *bacR* (DNA-binding response regulator); white, other genes. Download FIG S1, TIF file, 2.4 MB. Copyright © 2018 Furuta et al. 2018 Furuta et al. This content is distributed under the terms of the Creative Commons Attribution 4.0 International license.

10.1128/mSystems.00182-18.2 FIG S2 Mapping of long reads of CZC5. (A) Long reads of CZC5 mapped to the genome sequence of the Ames ancestor. (B) Long reads of CZC5 mapped to the genome sequence of CZC5. Arrows of different colors indicate protein coding regions (with protein products in parentheses) or rRNA as follows: green, rRNA operons; blue, *uppP* (undecaprenyl-diphosphatase); pink, *bcrB* (ABC transporter permease); red, *bcrA* (ABC transporter ATP-binding protein); yellow, *bacS* (sensor histidine kinase); orange, *bacR* (DNA-binding response regulator); white, other genes. Download FIG S2, PDF file, 0.9 MB. Copyright © 2018 Furuta et al. 2018 Furuta et al. This content is distributed under the terms of the Creative Commons Attribution 4.0 International license.

The deleted region was flanked by rRNA operons, which exist as 11 copies per genome of typical B. anthracis strains (Fig. 2B). Most of the rRNA operons harbored 16S, 23S, and 5S rRNA genes with the same order, orientation, and nucleotide sequence, suggesting that the deletion was caused by unequal crossing over between two long direct repeats of rRNA operons (24) (Fig. 2C). The flanking pair of rRNA operons was annotated as *rrnG* and *rrnH*. We, therefore, named the deletion DelGH.

Five genes deleted by DelGH were annotated as encoding undecaprenyl-diphosphatase, subunits of an ABC transporter, and members of a two-component system (Fig. 2D). The undecaprenyl-diphosphatase gene has sequence similarity to *uppP* of Escherichia coli (56% amino acid identity), which is known to confer resistance to bacitracin, a dodecapeptide antibiotic produced by Bacillus licheniformis and Bacillus subtilis (25). The ABC transporter genes and the two-component system genes also showed sequence similarity to *bcrAB* and *bacRS*, respectively, both of which sense bacitracin and regulate bacitracin resistance in B. licheniformis (26, 27). Hence, strains showing the deletion of these five genes were speculated to be more sensitive to bacitracin than strains without the deletion.

### Loss of bacitracin resistance by DelGH.

**FIG 3:**
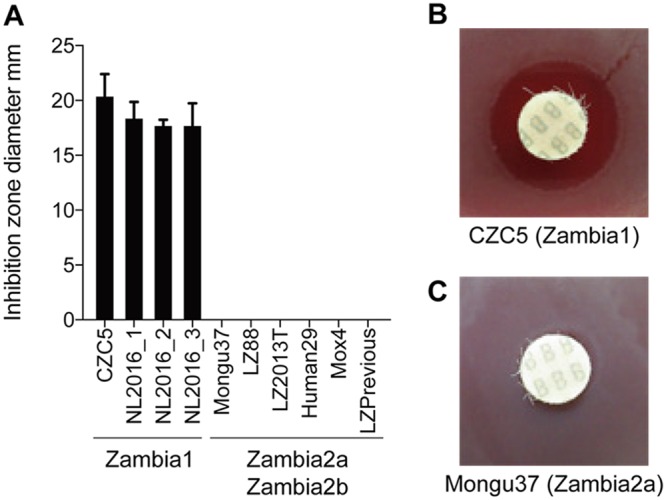
Bacitracin disk test of Zambian B. anthracis strains. (A) Mean diameter of the inhibition zone. The error bar indicates standard deviation (*n* = 3). No inhibition zone was observed among Zambia2a and Zambia2b strains. (B) An example of bacitracin disk test of CZC5. (C) An example of bacitracin disk test of Mongu37.

To confirm the influence of DelGH on bacitracin resistance, disk diffusion tests were conducted using representative Zambian isolates. Zambia1 strains showed an inhibitory zone of approximately 20 mm, whereas Zambia2a and Zambia2b strains showed no inhibitory zones (Fig. 3; see Fig. S3 in the supplemental material). This suggests that the deletion of genes conferring bacitracin resistance (DelGH) rendered the Zambia1 strains susceptible to bacitracin.

10.1128/mSystems.00182-18.3 FIG S3 Whole plates of the bacitracin disk test. (A) CZC5. (B) Mongu37. (C) LZ88. Download FIG S3, PDF file, 1.5 MB. Copyright © 2018 Furuta et al. 2018 Furuta et al. This content is distributed under the terms of the Creative Commons Attribution 4.0 International license.

**FIG 4:**
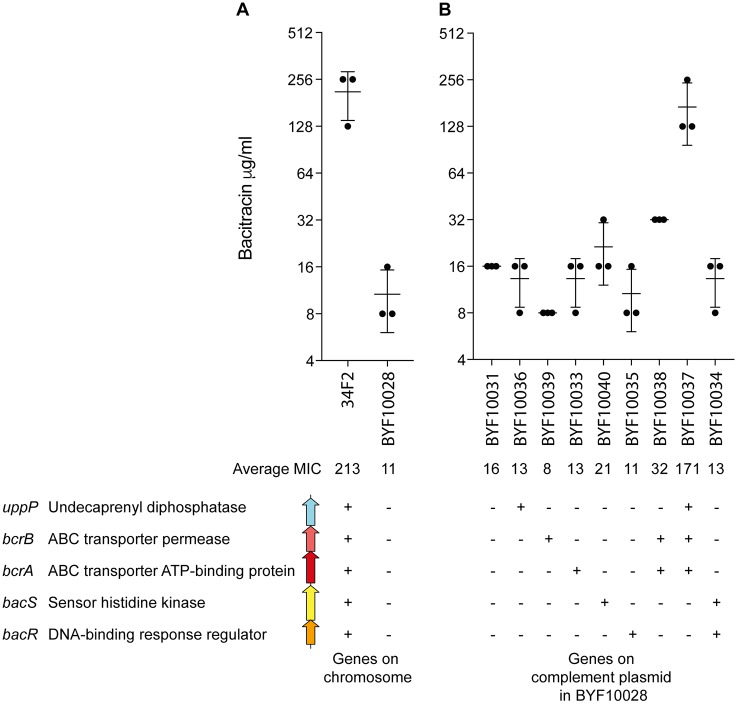
Effect of the deletion between rRNA operons G and H (DelGH) on bacitracin resistance. (A) MICs of the 34F2 vaccine strain and the derivative strain introduced with DelGH. Plus and minus signs indicate the presence and absence of bacitracin resistance genes, respectively, on the chromosome of each strain. (B) MICs of strains with DelGH complemented with bacitracin resistance gene(s). Plus and minus signs indicate the presence and absence of bacitracin resistance genes, respectively, on complementing plasmids. All strains in this panel lack all the bacitracin resistance genes on their chromosome. The error bar indicates the standard deviation (*n* = 3).

We further analyzed the contribution of each gene in the deleted region by strains constructed by artificially introducing DelGH to B. anthracis vaccination strain 34F2 (Table 2). First, the MICs of bacitracin were compared between the wild-type 34F2 strain and DelGH mutant (BYF10028). This showed an approximately 19-fold lower MIC in BYF10028 (Fig. 4A), supporting the results of the disk test for Zambian isolates. Next, BYF10028 was complemented with each gene or combination of genes in the deleted region to test the contribution of these genes to bacitracin resistance (Fig. 4B). Complementation of a single gene or a combination of ABC transporter and two-component system genes did not show any recovery of bacitracin resistance. Recovery of bacitracin resistance approximately to the level of the 34F2 strain was observed only with the complementation of a combination of *uppP* and both ABC transporter genes, suggesting that these three genes are required for bacitracin resistance.

**TABLE 2:** Strains and plasmids for bacitracin MIC measurement

Strain or plasmid	Description	Reference
Strains		
E. coli		
SM10	*thi thr leu tonA lacY supE recA* RP4-2-Tc::Mu (Km)	64
BYF684	SM10 harboring pYF684	This study
S17.1	*thi pro hsdR* RP4-2-Tc::Mu Km::Tn*7* (Tp Sm)	64
BYF701	S17.1 harboring pRP1099	This study
BYF794	S17.1 harboring pYF794	This study
BYF796	S17.1 harboring pYF796	This study
BYF797	S17.1 harboring pYF797	This study
BYF798	S17.1 harboring pYF798	This study
BYF799	S17.1 harboring pYF799	This study
BYF800	S17.1 harboring pYF800	This study
BYF801	S17.1 harboring pYF801	This study
BYF802	S17.1 harboring pYF802	This study
BYF803	S17.1 harboring pYF803	This study
B. anthracis		
34F2	B. anthracis vaccine strain	62
BYF10028	34F2 with the region between *rrnG* and *rrnH* deleted (DelGH)	This study
BYF10031	BYF10028 harboring pYF794	This study
BYF10033	BYF10028 harboring pYF798	This study
BYF10034	BYF10028 harboring pYF799	This study
BYF10035	BYF10028 harboring pYF800	This study
BYF10036	BYF10028 harboring pYF801	This study
BYF10037	BYF10028 harboring pYF796	This study
BYF10038	BYF10028 harboring pYF797	This study
BYF10039	BYF10028 harboring pYF802	This study
BYF10040	BYF10028 harboring pYF803	This study

Plasmids		
pRP1028	Vector for introduction of I-SceI recognition site	61
pYF684	pRP1028 cloned with sequence around *bcrB* of 34F2	This study
pRP1099	Vector for introduction of the gene encoding I-SceI	61
pYF794	pRP1099 without I-SceI and AmCyan	This study
pYF796	pYF794 with cloned *uppP*, *bcrB*, and *bcrA* of 34F2	This study
pYF797	pYF794 with cloned *bcrB* and *bcrA* of 34F2	This study
pYF798	pYF794 with cloned *bcrA* of 34F2	This study
pYF799	pYF794 with cloned *bacS* and *bacR* of 34F2	This study
pYF800	pYF794 with cloned *bacR* of 34F2	This study
pYF801	pYF794 with cloned *uppP* of 34F2	This study
pYF802	pYF794 with cloned *bcrB* of 34F2	This study
pYF803	pYF794 with cloned *bacS* of 34F2	This study

### Occurrence of large genomic deletions during the evolution of *B. anthracis*.

**FIG 5:**
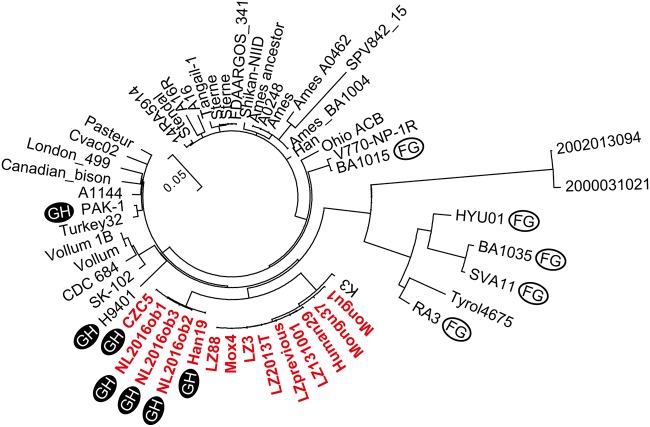
Phylogenetic tree of Zambian and non-Zambian strains of B. anthracis. Zambian strains are indicated in red and bold letters. Strains with the DelFG deletion are indicated by “FG” in a white circle. Strains with the DelGH deletion are indicated by “GH” in a black circle.

To understand the phylogenetic position of Zambian strains among B. anthracis strains, genome-wide SNPs were compared with genome sequences of other non-Zambian B. anthracis strains (Fig. 5; see Table S2 in the supplemental material). The results showed that Zambian strains clustered in the same clade, together with the K3 strain isolated in South Africa.

10.1128/mSystems.00182-18.8 TABLE S2 Bacillus anthracis strains whose complete genome sequences are available at the RefSeq database. Download Table S2, PDF file, 0.1 MB. Copyright © 2018 Furuta et al. 2018 Furuta et al. This content is distributed under the terms of the Creative Commons Attribution 4.0 International license.

We also searched for DelGH and genomic deletions between other pairs of rRNA operons in non-Zambian B. anthracis strains and identified such a deletion within a region flanked by different pairs of rRNA operons, *rrnF* and *rrnG*, which we here refer to as DelFG. DelFG resulted in the deletion of genes annotated as encoding class I *S*-adenosylmethionine (SAM)-dependent methyltransferase, RNA methyltransferase, and glycosyl transferase (see Fig. S4 in the supplemental material). All of these genes are involved in the modification of nucleotides, proteins, and membrane lipids. However, the targets and types of modification of these enzymes were unclear; thus, it is difficult to speculate about the influence of DelFG on the phenotype.

10.1128/mSystems.00182-18.4 FIG S4 Comparison of genomic regions encompassing the deletion DelFG. Genes are indicated by the number of the locus tag that in the full locus tag designation follows the prefix “GBAA_RS” for the Ames ancestor or “HYU01_RS” for HYU01. Arrows of different colors indicate protein coding regions (with protein products in parentheses) or rRNA as follows: green, rRNA operons; blue, *uppP* (undecaprenyl-diphosphatase); white, other genes. Download FIG S4, PDF file, 0.9 MB. Copyright © 2018 Furuta et al. 2018 Furuta et al. This content is distributed under the terms of the Creative Commons Attribution 4.0 International license.

Among the non-Zambian B. anthracis strains, DelFG was observed in BA1015, BA1035, HYU01, RA3, and SVA11, whereas DelGH was observed in H9401 and PAK-1. These deletions were confirmed by mapping short reads of strains to the reference genome sequence of the Ames ancestor (see Fig. S5 in the supplemental material). Most of the strains with the same deletion grouped in a single clade: strains RA3, HYU01, SVA11, and BA1035 carrying the DelFG deletion formed one cluster, and Zambia1 strains carrying the DelGH deletion grouped in another clade. This implies the occurrence of single deletion event in a common ancestor, followed by vertical transmission of the deletion. Three strains, including H9401 and PAK-1 with DelGH and BA1015 with DelFG, were phylogenetically distant from both the DelFG and DelGH clades, suggesting that these deletions occurred at a low frequency but multiple times during the course of evolution of B. anthracis.

10.1128/mSystems.00182-18.5 FIG S5 Sequence coverage of short reads of strains with deletion between rRNA operons. The short reads were mapped to the reference genome sequence of the Ames ancestor strain. (A) PAK-1. (B) BA1015. (C) SVA11. (D) BA1035. Arrows of different colors indicate protein coding regions (with protein products in parentheses) or rRNA as follows: green, rRNA operons; blue, *uppP* (undecaprenyl-diphosphatase); pink, *bcrB* (ABC transporter permease); red, *bcrA* (ABC transporter ATP-binding protein); yellow, *bacS* (sensor histidine kinase); orange, *bacR* (DNA-binding response regulator); white, other genes. Download FIG S5, PDF file, 2.7 MB. Copyright © 2018 Furuta et al. 2018 Furuta et al. This content is distributed under the terms of the Creative Commons Attribution 4.0 International license.

### Structure of a bacitracin resistance gene cluster in *Bacillus* species.

To address whether the deletion of the whole bacitracin resistance gene cluster possibly via unequal crossing over between rRNA operons occurred only in B. anthracis or could be found in other Bacillus species, we searched for homologs of genes within this cluster in genomic sequences of strains of Bacillus species (see Table S3 in the supplemental material).

10.1128/mSystems.00182-18.9 TABLE S3 Locus tags of gene homologs within the bacitracin resistance gene cluster in strains of *Bacillus* species. Download Table S3, PDF file, 0.1 MB. Copyright © 2018 Furuta et al. 2018 Furuta et al. This content is distributed under the terms of the Creative Commons Attribution 4.0 International license.

**FIG 6:**
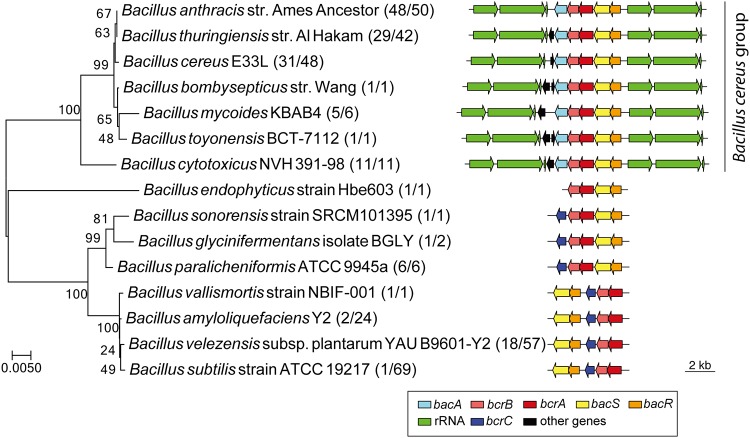
Structure of the bacitracin resistance gene cluster in Bacillus species. The phylogenetic tree was constructed based on the 16S rRNA sequences of each species. Numbers following the names of strains indicate the number of strains of the species possessing the bacitracin resistance gene cluster out of the number of strains analyzed.

The structure of the bacitracin resistance gene cluster flanked by rRNA operons was found only in strains of the B. cereus group, including B. anthracis (Fig. 6). More than 60% of strains of each species of the B. cereus group were found with the gene cluster flanked by rRNA operons. Thus, it is likely that the bacitracin resistance gene operon was present in the common ancestor of the B. cereus group but was lost in some strains during the evolution of each species of the B. cereus group.

Other Bacillus species either did not possess the cluster or possessed it only at a very low frequency. For example, only two Bacillus amyloliquefaciens strains and one B. subtilis strain were found to possess the cluster, suggesting that these species acquired the cluster at a very low frequency. In addition, the *uppP* gene was substituted for with the *bcrC* gene homolog, whose product has the same activity of undecaprenyl-diphosphatase but is composed of different domain sequences (26, 28). Differences in the relative positions of the two-component system genes were also observed in Bacillus vallismortis, B. amyloliquefaciens, Bacillus velezensis, and B. subtilis. Taken together, the structure of bacitracin resistance gene cluster flanked by rRNA operons and the presence of *uppP* homolog within the cluster was specifically conserved among species of the B. cereus group.

## DISCUSSION

In this study, we conducted genome sequencing of Zambian isolates of B. anthracis to determine their genetic diversity. Results revealed three groups based on genome-wide SNPs and the deletion of a large genomic region encompassing the bacitracin resistance genes, possibly via unequal crossing over between a pair of rRNA operons flanking the region. Experiments with natural isolates and artificially constructed strains of B. anthracis confirmed that the deletion of bacitracin resistance gene cluster results in the loss of bacitracin resistance. The deletion of this gene cluster was also identified in a few other non-Zambian B. anthracis strains, and the structure of this genomic region was highly conserved only in species of the B. cereus group.

All of the Zambian strains of B. anthracis in this study were classified in the A.Br.005/006 group, according to the canonical SNPs (see Table S4 in the supplemental material) and clustered with the K3 strain isolated in South Africa, according to the clustering based on genome-wide SNPs (Fig. 5). These results were consistent with previous works on Zambian B. anthracis strains (13, 22, 29). Within the closely related Zambian strains, genome-wide SNPs clustered the strains into three groups—Zambia1, Zambia2a, and Zambia2b—which showed some relationship with the sampling location. The genomic deletion resulted in a clear phenotypic difference between Zambia1 and the other groups, but not between the Zambia2a and Zambia2b groups. This information on the genetic diversity and phenotypic differences among B. anthracis strains would be useful for the development of genetic markers to differentiate among Zambian B. anthracis strains. Additionally, these markers would help in determining the strains’ group in the event of an anthrax outbreak in Zambia in the future.

10.1128/mSystems.00182-18.10 TABLE S4 Canonical SNPs in B. anthracis Zambian isolates. Download Table S4, PDF file, 0.1 MB. Copyright © 2018 Furuta et al. 2018 Furuta et al. This content is distributed under the terms of the Creative Commons Attribution 4.0 International license.

The large genomic deletion DelGH included the bacitracin resistance gene cluster and resulted in a significant loss of bacitracin resistance, suggesting bacitracin as an alternative antibiotic when treating anthrax caused by Zambia1 strains. Bacitracin is a cyclic peptide antibiotic, which inhibits bacterial cell wall synthesis by sequestering undecaprenyl-pyrophosphate, an essential carrier lipid (25). Anthrax is usually treated with antibiotics such as benzylpenicillin, amoxicillin, doxycycline, and ciprofloxacin or ofloxacin (30, 31). To our knowledge, B. anthracis infections in animals and in humans are quite rarely treated with bacitracin in Zambia and in other countries. Reports of B. anthracis strains resistant to antibiotics are rare; however, some studies have shown that long-term treatment of B. anthracis *in vitro* with increasing amounts of antibiotics results in strains with higher MICs for various antibiotics, including fluoroquinolones (32–36). Therefore, it is important to prepare for and suppress the emergence of antibiotic-resistant strains by having an alternative choice of antibiotics, although bacitracin treatment might be limited to cutaneous anthrax because of its nephrotoxic effect (37).

In Bacillus, two main gene clusters conferring bacitracin resistance are known: one of these clusters is present in species of the B. cereus group, including B. anthracis, which includes the genes *bacRS* and *bcrAB*, and the other cluster is present in other Bacillus species, such as B. subtilis, which contains *bceRSAB* (38, 39). Both gene clusters consist of two-component system (*bacRS* and *bceRS*) and ABC transporter (*bcrAB* and *bceAB*) genes. The cluster of *bacRS* and *bcrAB* usually possesses another gene with the function of the undecaprenyl-diphosphatase gene, *uppP* or *bcrC*, immediately adjacent to the four genes (Fig. 6). Genes with sequence similarity to *uppP* or *bcrC* are also found in the genome of species with *bceRSAB* but are usually not adjacent to the cluster. UppP and BcrC have the same activity as undecaprenyl-diphosphatase, but belong to a different protein superfamily (25, 40). It is difficult to determine how the two genes *uppP* and *bcrC* were swapped in the immediate vicinity of the *bacRS* and *bcrAB* cluster during the evolution of Bacillus species (Fig. 6).

The activity and regulation of proteins encoded by the *bacRS*, *bcrAB*, and *uppP* cluster have been studied less than those encoded by the *bceRSAB* cluster, which is present in the model organism B. subtilis (38, 39, 41–45). Undecaprenyl-diphosphatase activity of UppP has been shown to confer resistance to bacitracin when the E. coli homolog of *uppP* was overexpressed in E. coli cells (46); however, this was not the case in B. anthracis (Fig. 4B). We observed full recovery of bacitracin resistance only when UppP was expressed together with BcrA and BcrB in the strain with DelGH. In the *bceRSAB* cluster, it is hypothesized that the ABC transporter consists of BceA and BceB and functions as a flippase to flip the undecaprenyl-pyrophosphate and bacitracin complex to the outer leaflet of the membrane, thus, promoting the reaction of membrane proteins that utilize bacitracin as a substrate (43). If the ABC transporter that consists of BcrA and BcrB also possesses such flippase activity, the ABC transporter may promote the activity of UppP, which is also a membrane protein (25), thus, leading to a complete recovery of bacitracin resistance. On the other hand, BacR, the response regulator of the *bacRS* two-component system, has been shown to act as a negative regulator of the *bcrA* gene in B. licheniformis (27). This is consistent with our results showing that genes of the two-component system are not required for the recovery of bacitracin resistance in strains carrying the DelGH deletion (Fig. 4).

Deletion of the bacitracin resistance gene cluster was identified in several B. anthracis strains and among species of the B. cereus group. This deletion was most likely caused by unequal crossing over between a pair of rRNA operons flanking the gene cluster. Genome rearrangement due to unequal crossing over between direct repeats is a well-studied phenomenon in bacteria (47). This results not only in gene deletion but also gene amplification and an increase in the expression of genes in the amplified region (47). The rRNA operon has been shown to cause amplification of the flanking region in Salmonella enterica serovar Typhimurium and E. coli (48, 49). Such genome amplification has been suggested as a mechanism to increase the antibiotic resistance of bacteria, thus, increasing their ability to survive under high antibiotic pressure (50). For example, amplification of antibiotic resistance genes due to flanking insertion sequences approximately 1 kb in size has increased the resistance of E. coli to specific drugs (51–54). Decreased antibiotic pressure results in the reduction of copy numbers of the amplified region, again by unequal crossing over, to revert the copy number before amplification, thus maximizing the fitness of bacteria. The bacitracin resistance gene cluster in species of the B. cereus group may also have experienced both deletion and amplification to flexibly control the level of bacitracin resistance to survive in the environment with bacitracin secreted by different Bacillus species (25), although further experiments, such as culturing of B. anthracis strains *in vitro* under gradually increasing concentrations of bacitracin, are required to confirm the plasticity and contribution of the gene cluster.

In conclusion, our sequencing analysis of Zambian isolates of B. anthracis revealed three groups with different SNPs and large genomic deletions affecting bacitracin resistance. The pair of rRNA operons flanking the bacitracin resistance gene cluster are an ideal target for unequal crossing over, which not only leads to the deletion of the whole region but also has the potential for amplification of the region, to possibly enhance bacitracin resistance. Although further experimental confirmation is required, our data suggest that this gene composition contributes to the evolution of B. anthracis strains by controlling the bacitracin resistance phenotype for adaptation.

## MATERIALS AND METHODS

### *B. anthracis* natural isolates.

B. anthracis strains were isolated from various hosts from different locations in Zambia in different years (Table 1). All of the Zambian isolates were handled at the BSL3 facility at the Hokudai Center for Zoonosis Control in Zambia. Samples from hippopotamuses (Hippopotamus amphibius), African elephants (Loxodonta africana), and cattle (Bos taurus) were collected from tissues and fluid of carcasses. Soil samples (approximately 25 mg) were collected from a depth of 15 cm. Isolation of B. anthracis strains from samples was performed as described previously (55). Briefly, 1 g of a specimen was suspended in 10 ml of sterilized saline and incubated at 75°C for 20 min to enrich for spores, followed by spreading the samples on brain heart infusion (BHI) agar plates with 10% (vol/vol) sheep blood agar. Colonies with a “medusa head” were identified as B. anthracis. These were inoculated in LB medium and incubated 37°C overnight on a shaker. The resulting cultures were stored at −80°C as glycerol stocks. For genomic DNA extraction, glycerol stocks were inoculated and streaked on BHI agar plates with 10% sheep blood and incubated at 37°C for 16 h. Colonies were harvested and used for genomic DNA extraction with the QIAamp PowerFecal DNA kit (Qiagen, Hilden, Germany).

A B. anthracis strain isolated from a human case of anthrax was provided by the School of Veterinary Medicine, University of Zambia, Zambia.

### Sequencing and genome analysis of Zambian strains.

For whole-genome sequencing, 10 ng of genomic DNA was used to prepare genomic sequencing libraries using the Nextera DNA library preparation kit (Illumina, San Diego, CA) and sequenced using the Illumina MiSeq platform (Illumina, San Diego, CA). Paired-end reads with a read length of 300 bp were obtained. These short reads were used for SNP detection, reconstruction of a consensus sequence, and detection of genomic deletion by mapping to the reference genome (chromosome, pXO1, and pXO2) of the B. anthracis Ames ancestor (RefSeq accession no. NC_007530.2, NC_007322.2, and NC_007323.3, respectively) using Snippy v3.2 (https://github.com/tseemann/snippy), which maps reads using bwa mem (56) and performs variant calling using Freebayes (57) with default parameters.

To obtain long reads from strain CZC5, 5 μg of genomic DNA of CZC5 was used to construct a sequencing library with the SMRTbell Template Prep kit (Pacific Biosciences, Menlo Park, CA) and sequenced using the PacBio RS platform (Pacific Biosciences, Menlo Park, CA) with a 360-min movie time. This generated sequence reads of approximately 130 Mbp, with an average read length of 5,732 bp. *De novo* assembly was also conducted for CZC5 on the SMRT portal using the pipeline script RS_HGAP_Assembly. The assembled sequences were corrected with short reads, produced by the Illumina MiSeq platform as described above, by mapping the short reads to the assembled sequences using bwa mem with the default parameter, followed by using Pilon (58) with “–changes –fix snps,indels,gaps” options. This procedure resulted in changes of 19 single-nucleotide indels and one SNP.

For visualization of DelGH, long reads were mapped to the reference genome of the B. anthracis Ames ancestor and CZC5 using minimap2 v2.5 (59) with default parameters, followed by visualization with IGV v2.3.97 (60).

### Bacitracin disk test.

Glycerol stock of each B. anthracis strain was inoculated on a BHI plate with 10% (vol/vol) sheep blood and incubated at 37°C for 16 h. Bacterial colonies were harvested, resuspended in phosphate-buffered saline, and diluted to an optical density at 600 nm (OD_600_) of approximately 0.2, followed by plating of 300 μl on blood agar plates. A bacitracin disk (Sigma-Aldrich, St. Louis, MO; catalog no. 08382-50DISCS-F [0.04 U/disk]) was placed at the center of each plate and incubated at 37°C for 16 h. CFU were measured for the resuspended and diluted samples of each strain, and samples from each strain were confirmed to have less than a 10-fold difference in CFU.

### Strain construction.

Strains were constructed to measure the MIC values (Table 2) using the markerless allelic exchange strategy (61). To construct the strain with DelGH in B. anthracis 34F2 (62), genomic sequences (500 bp) flanking the *bcrB* gene were amplified by PCR using genomic DNA of 34F2 as a template and inserted in pRP1028 together with the recognition site of I-SceI using Gibson assembly (63). The constructed plasmid, pYF684, was transformed into E. coli SM10 cells (64) and introduced into the 34F2 strain by conjugation. The strain with the plasmid integrated into its genomic DNA was selected and conjugated with pRP1099 harboring the gene expressing I-SceI from E. coli S17.1 (64) to induce double-strand breaks (DSBs) in the genomic DNA of the B. anthracis intermediate strain. The DSBs promoted homologous recombination between rRNA operons G and H flanking the entire bacitracin resistance gene cluster rather than the introduced flanking sequence of the *bcrB* gene, which resulted in the 34F2 derivative strain with DelGH, BYF10028. Strains with the *bcrB* gene or other genes of the bacitracin resistance gene cluster deleted could not be constructed, probably because of much higher probability for homologous recombination between 4.7-kb rRNA operons than between 500-bp flanking sequence of the target gene. Introduction of DelGH in BYF10028 was confirmed by sequencing on the MinION platform using flowcell R9.5 and the Rapid barcode sequencing kit SQK-RBK001 (Oxford Nanopore Technologies, Oxford Science Park, United Kingdom). Reads were mapped onto the reference genome sequence of the B. anthracis Ames ancestor strain and CZC5 strain using minimap2 v2.5 (59) with default parameters (see Fig. S6 in the supplemental material).

10.1128/mSystems.00182-18.6 FIG S6 Mapping of long reads of BYF10028. (A) Long reads of BYF10028 mapped to the reference genome sequence of the Ames ancestor. (B) Long reads of BYF10028 mapped to the genome sequence of CZC5. Arrows of different colors indicate protein coding regions (with protein products in parentheses) or rRNA as follows: green, rRNA operons; blue, *uppP* (undecaprenyl-diphosphatase); pink, *bcrB* (ABC transporter permease); red, *bcrA* (ABC transporter ATP-binding protein); yellow, *bacS* (sensor histidine kinase); orange, *bacR* (DNA-binding response regulator); white, other genes. Download FIG S6, PDF file, 1.6 MB. Copyright © 2018 Furuta et al. 2018 Furuta et al. This content is distributed under the terms of the Creative Commons Attribution 4.0 International license.

For the complementation of bacitracin resistance genes, genes were cloned under the control of their native promoter in pRP1099 with substituting genes encoding I-SceI and AmCyan. The resulting plasmids, pYF794 to 803, were transformed into E. coli S17.1 cells and introduced into BYF10028 by conjugation. Attempts to introduce complementation plasmid with four genes or more into B. anthracis cells were unsuccessful.

### Measurement of MIC for bacitracin.

Glycerol stocks of strains were inoculated on BHI agar plates with or without 20 μg/ml kanamycin and incubated at 37°C overnight. A single colony was isolated, inoculated in BHI with or without 20 μg/ml kanamycin, and incubated at 37°C on a shaker overnight. Ten microliters of overnight cultures was added to 980 μl of BHI and then added with 10 μl of a 2-fold serial dilution series of bacitracin solution (Sigma-Aldrich, St. Louis, MO; catalog no. B0125, lot no. 115M4104V, with a potency of 77,120 U/g) to have cultures in final bacitracin concentrations of 0, 4, 8, 16, 32, 64, 128, 256, and 512 μg/ml. Cultures were incubated at 37°C on a shaker overnight, and the OD was measured at 595 nm using Multiskan FC (Thermo Fisher Scientific, Waltham, MA). The lowest concentration of bacitracin with an OD of <0.1 was determined as the MIC.

### Comparison of whole-genome sequences of non-Zambian strains.

Genome sequences of non-Zambian B. anthracis strains registered as a complete assembly in RefSeq as of 18 July 2018 were used for the construction of a phylogenetic tree (Table S2). Genome sequences of the strains obtained in artificial evolution experiments were not used in this analysis to focus only on natural isolates (65).

Reconstructed consensus sequences of Zambian strains and whole-genome sequences of non-Zambian B. anthracis strains were aligned and subjected to phylogenetic analysis by Parsnp included in the Harvest suite (66). Whole-genome sequences were compared using Mauve (67, 68) to detect genomic deletions between rRNA operons. Genome maps were plotted by genoPlotR (69). Short sequence reads of strains PAK-1 (SRR2155549), BA1015 (SRR2175366), SVA11 (SRR974942), and BA1035 (SRR2174564) were obtained from the DNA Data Bank of Japan Sequence Read Archive (DRA) database and used to confirm the large genomic deletion by mapping.

### Detection of bacitracin resistance genes in *Bacillus* species.

Genome sequences of strains of Bacillus species that are registered with complete genome sequences were downloaded from the RefSeq database as of 18 July 2018 (Table S3). Genome sequences were searched for bacitracin resistance genes with tblastn (70) using amino acid sequences of the following accession numbers as queries: BcrC, NP_391534.1; UppP, WP_001280040.1; BcrB, WP_000247643.1; BcrA, WP_000074565.1; BacS, WP_000686989.1; and BacR, WP_000651978.1. The top-most hits according to amino acid percentage of identity with an alignment length covering >80% of the query length and with >40% sequence similarity were assumed as homologs. Strains with four or five of the query genes in tandem, allowing insertion of up to three genes, were determined to harbor the bacitracin resistance gene cluster. A phylogenetic tree was constructed using 16S rRNA sequence of each species in MEGA7 (71) by the neighbor-joining method with the Kimura two-parameter model (72).

### Accession number(s).

DNA sequences generated in this study were deposited in the DRA database under accession no. DRR118317 to DRR118319 and DRR125653 to DRR125657 for reads of Zambian isolates using the Illumina MiSeq platform (Illumina, San Diego, CA), DRR118320 for the CZC5 strain using the PacBio RS platform (Pacific Biosciences, Menlo Park, CA), and DRR147392 and DRR147393 for the 34F2 and BYF10028 strains, respectively, using the MinION platform (Oxford Nanopore Technologies, Oxford Science Park, United Kingdom). The complete whole-genome sequence of CZC5 was deposited in GenBank under the following accession numbers: chromosome, AP018443; pXO1, AP018444; and pXO2, AP018445.
