# Supplementary material for: Loss of Bacitracin Resistance Due to a Large Genomic Deletion among Bacillus anthracis Strains
Source: mSystems. 2018 Oct 30;3(5):e00182-18. doi: 10.1128/mSystems.00182-18 (PMC6208641; doi:10.1128/mSystems.00182-18)
Supplement: TABLE S1 [file sys005182281st1.pdf]

Table S1. SNPs in *B. anthracis* Zambian isolates.

Reference ID	Chromosome/plasmid	Position	Reference	NL2016_1	NL2016_2	NL2016_3	CZC5	Han19	Mongu1	Mongu37	Human29	Mox4	Lzprevious	LZ2013T	LZ88	LZ3	LZ131001	Locus tag[a]	Gene	Product	Effect
NC_007322	pXO1	1405	T	T	T	T	T	T	C	C	C	C	C	C	C	C	C	GBAA_RS28465	-	hypothetical protein	missense_variant c.361A>G p.Lys121Glu
NC_007322	pXO1	7452	C	T	T	T	T	T	T	T	T	T	T	T	T	T	T	GBAA_RS28500	ltrA	group II intron reverse transcriptase/maturase	synonymous_variant c.1035G>A p.Ser345Ser
NC_007322	pXO1	7968	A	G	G	G	G	G	A	A	A	A	A	A	A	A	A	GBAA_RS28500	ltrA	group II intron reverse transcriptase/maturase	synonymous_variant c.519T>C p.Leu173Leu
NC_007322	pXO1	17892	A	G	G	G	G	G	G	G	G	G	G	G	G	G	G	GBAA_RS28530	-	hypothetical protein	synonymous_variant c.1245T>C p.Asp415Asp
NC_007322	pXO1	33638	T	C	C	C	C	C	C	C	C	C	C	C	C	C	C	IGR	-	-	-
NC_007322	pXO1	34071	C	C	C	C	C	C	C	C	C	C	T	C	C	C	C	GBAA_RS28590	-	nucleotidyltransferase domain-containing protein	missense_variant c.143G>A p.Gly48Asp
NC_007322	pXO1	40791	T	C	C	C	C	C	C	C	C	C	C	C	C	C	C	GBAA_RS28635	-	nucleotidyltransferase	missense_variant c.548A>G p.Gln183Arg
NC_007322	pXO1	41635	C	C	C	C	C	C	T	T	T	T	T	T	T	T	T	GBAA_RS28640	-	TetR/AcrR family transcriptional regulator	missense_variant c.304G>A p.Asp102Asn
NC_007322	pXO1	48621	G	A	A	A	A	A	G	A	A	A	A	A	G	A	G	IGR	-	-	-
NC_007322	pXO1	48648	G	C	C	C	C	C	G	C	C	C	C	C	G	C	G	IGR	-	-	-
NC_007322	pXO1	48649	A	C	C	C	C	C	A	C	C	C	C	C	A	C	A	IGR	-	-	-
NC_007322	pXO1	48650	G	C	C	C	C	C	G	C	C	C	C	C	G	C	G	IGR	-	-	-
NC_007322	pXO1	48652	G	A	A	A	A	A	G	A	A	A	A	A	G	A	G	IGR	-	-	-
NC_007322	pXO1	48665	G	C	C	C	C	C	C	C	C	C	C	C	C	C	C	IGR	-	-	-
NC_007322	pXO1	48753	C	T	T	T	T	T	C	C	C	C	C	C	C	C	C	GBAA_RS28675	-	IS4 family transposase	synonymous_variant c.1411_1416delCTAAGGinsTTGAGA p.473
NC_007322	pXO1	48756	T	C	C	C	C	C	T	T	T	T	T	T	T	T	T	GBAA_RS28675	-	IS4 family transposase	synonymous_variant c.1411_1416delCTAAGGinsTTGAGA p.473
NC_007322	pXO1	48758	G	A	A	A	A	A	G	G	G	G	G	G	G	G	G	GBAA_RS28675	-	IS4 family transposase	synonymous_variant c.1411_1416delCTAAGGinsTTGAGA p.473
NC_007322	pXO1	48769	T	T	T	T	T	T	T	C	T	T	T	T	T	T	T	GBAA_RS28675	-	IS4 family transposase	missense_variant c.1400A>G p.Glu467Gly
NC_007322	pXO1	48775	G	A	A	A	A	A	G	G	G	G	G	G	G	G	G	GBAA_RS28675	-	IS4 family transposase	missense_variant c.1394C>T p.Ala465Val
NC_007322	pXO1	48797	T	C	C	C	C	C	T	T	T	T	T	T	T	T	T	GBAA_RS28675	-	IS4 family transposase	missense_variant c.1368_1372delAACTAinsGACAG p.Ile458Val
NC_007322	pXO1	48798	A	T	T	T	T	T	A	A	A	A	A	A	A	A	A	GBAA_RS28675	-	IS4 family transposase	missense_variant c.1368_1372delAACTAinsGACAG p.Ile458Val
NC_007322	pXO1	48801	T	C	C	C	C	C	T	T	T	T	T	T	T	T	T	GBAA_RS28675	-	IS4 family transposase	missense_variant c.1368_1372delAACTAinsGACAG p.Ile458Val
NC_007322	pXO1	48825	T	C	C	C	C	C	T	T	T	T	T	T	T	T	T	GBAA_RS28675	-	IS4 family transposase	missense_variant c.1343_1344delAAinsGG p.Gln448Arg
NC_007322	pXO1	48826	T	C	C	C	C	C	T	T	T	T	T	T	T	T	T	GBAA_RS28675	-	IS4 family transposase	missense_variant c.1343_1344delAAinsGG p.Gln448Arg
NC_007322	pXO1	48858	A	G	G	G	G	G	A	A	A	A	A	A	A	A	A	GBAA_RS28675	-	IS4 family transposase	synonymous_variant c.1311T>C p.Ile437Ile
NC_007322	pXO1	49038	G	G	G	G	G	G	A	A	A	A	A	A	A	A	A	GBAA_RS28675	-	IS4 family transposase	synonymous_variant c.1131C>T p.Tyr377Tyr
NC_007322	pXO1	61403	C	T	T	T	T	T	T	T	T	T	T	T	T	T	T	IGR	-	-	-
NC_007322	pXO1	65158	T	T	T	T	T	T	T	T	T	C	C	C	C	C	T	GBAA_RS28745	-	hypothetical protein	synonymous_variant c.96T>C p.Ser32Ser
NC_007322	pXO1	65304	G	G	G	G	G	G	A	A	A	A	A	A	A	A	A	GBAA_RS28745	-	hypothetical protein	missense_variant c.242G>A p.Arg81His
NC_007322	pXO1	74635	T	C	C	C	C	C	C	C	C	C	C	C	C	C	C	GBAA_RS28790	-	hypothetical protein	missense_variant c.517T>C p.Ser173Pro
NC_007322	pXO1	77518	A	G	G	G	G	G	G	G	G	G	G	G	G	G	G	GBAA_RS28805	-	hypothetical protein	missense_variant c.130A>G p.Ser44Gly
NC_007322	pXO1	83185	A	A	A	A	A	A	A	G	A	A	A	A	A	A	A	IGR	-	-	-
NC_007322	pXO1	87910	C	T	T	T	T	T	T	T	T	T	T	T	T	T	T	GBAA_RS28865	-	hypothetical protein	synonymous_variant c.300C>T p.Gly100Gly
NC_007322	pXO1	90887	A	G	G	G	G	G	G	G	G	G	G	G	G	G	G	GBAA_RS28880	-	DUF87 domain-containing protein	missense_variant c.1523A>G p.Asp508Gly
NC_007322	pXO1	99355	T	C	C	C	C	C	C	C	C	C	C	C	C	C	C	GBAA_RS28915	-	hypothetical protein	missense_variant c.188T>C p.Met63Thr
NC_007322	pXO1	104414	G	A	A	A	A	A	A	A	A	A	A	A	A	A	A	GBAA_RS28940	-	hypothetical protein	missense_variant c.268C>T p.Leu90Phe
NC_007322	pXO1	104458	C	C	C	C	C	C	T	T	T	T	T	T	T	T	T	GBAA_RS28940	-	hypothetical protein	missense_variant c.224G>A p.Ser75Asn
NC_007322	pXO1	105630	A	A	A	A	A	A	G	G	G	G	G	G	G	G	G	IGR	-	-	-
NC_007322	pXO1	106456	T	C	C	C	C	C	C	C	C	C	C	C	C	C	C	IGR	-	-	-
NC_007322	pXO1	110821	T	G	G	G	G	G	T	T	T	T	T	T	T	T	T	IGR	-	-	-
NC_007322	pXO1	117192	T	C	C	C	C	C	C	C	C	C	C	C	C	C	C	IGR	-	-	-
NC_007322	pXO1	119265	A	G	G	G	G	G	A	A	A	A	A	A	A	A	A	GBAA_RS29020	-	hypothetical protein	synonymous_variant c.84T>C p.Asn28Asn
NC_007322	pXO1	123207	C	T	T	T	T	T	T	T	T	T	T	T	T	T	T	GBAA_RS29035	-	calmodulin-sensitive adenylate cyclase	synonymous_variant c.600C>T p.Ser200Ser
NC_007322	pXO1	124736	A	C	C	C	C	C	C	C	C	C	C	C	C	C	C	GBAA_RS29035	-	calmodulin-sensitive adenylate cyclase	missense_variant c.2129A>C p.His710Pro
NC_007322	pXO1	129231	C	T	T	T	T	T	C	C	C	C	C	C	C	C	C	IGR	-	-	-
NC_007322	pXO1	139613	C	C	C	C	C	C	T	T	T	T	T	T	T	T	T	GBAA_RS29095	-	germination protein XA	synonymous_variant c.1338C>T p.Leu446Leu
NC_007322	pXO1	141921	T	G	G	G	G	T	G	G	G	G	G	G	G	G	G	IGR	-	-	-
NC_007322	pXO1	141922	T	A	A	A	A	T	A	A	A	A	A	A	A	A	A	IGR	-	-	-
NC_007322	pXO1	141923	T	A	A	A	A	T	A	A	A	A	A	A	A	A	A	IGR	-	-	-
NC_007322	pXO1	141924	C	A	A	A	A	C	A	A	A	A	A	A	A	A	A	IGR	-	-	-
NC_007322	pXO1	143973	C	T	T	T	T	T	T	T	T	T	T	T	T	T	T	GBAA_RS29110	-	protective antigen	synonymous_variant c.195C>T p.Thr65Thr
NC_007322	pXO1	145577	C	T	T	T	T	T	T	T	T	T	T	T	T	T	T	GBAA_RS29110	-	protective antigen	missense_variant c.1799C>T p.Ala600Val
NC_007322	pXO1	146834	T	C	C	C	C	C	T	T	T	T	T	T	T	T	T	IGR	-	-	-
NC_007322	pXO1	147905	T	C	C	C	C	C	C	C	C	C	C	C	C	C	C	IGR	-	-	-
NC_007322	pXO1	157598	A	G	G	G	G	G	G	G	G	G	G	G	G	G	G	IGR	-	-	-
NC_007322	pXO1	164239	T	C	C	C	C	C	C	C	C	C	C	C	C	C	C	GBAA_RS29220	-	IS3 family transposase	stop_lost c.871T>C p.Ter291Glnext*?
NC_007322	pXO1	171972	C	T	T	T	T	T	T	T	T	T	T	T	T	T	T	GBAA_RS29255	-	hypothetical protein	missense_variant c.97G>A p.Glu33Lys
NC_007322	pXO1	180878	G	A	A	A	A	A	A	A	A	A	A	A	A	A	A	GBAA_RS31350	-	transposase	missense_variant c.473C>T p.Ser158Phe
NC_007322	pXO1	180979	A	A	A	A	A	A	G	G	G	G	G	G	G	G	G	GBAA_RS31350	-	transposase	synonymous_variant c.372T>C p.His124His
NC_007323	pXO2	1502	G	G	G	G	G	G	A	A	A	A	A	A	A	A	A	IGR	-	-	-
NC_007323	pXO2	8639	C	T	T	T	T	T	T	T	T	T	T	T	T	T	T	GBAA_RS28035	-	hypothetical protein	synonymous_variant c.2430G>A p.Val810Val
NC_007323	pXO2	8654	T	T	T	T	T	T	C	C	C	C	C	C	C	C	C	GBAA_RS28035	-	hypothetical protein	synonymous_variant c.2415A>G p.Ala805Ala
NC_007323	pXO2	9106	T	G	G	G	G	G	G	G	G	G	G	G	G	G	G	GBAA_RS28035	-	hypothetical protein	missense_variant c.1963A>C p.Lys655Gln
NC_007323	pXO2	12266	A	C	C	C	C	C	A	A	A	A	A	A	A	A	A	GBAA_RS28040	-	conjugal transfer protein	stop_gained c.1458T>G p.Tyr486*
NC_007323	pXO2	20966	G	G	G	G	G	G	A	A	A	A	A	A	A	A	A	GBAA_RS31140	-	hypothetical protein	missense_variant c.1423C>T p.Pro475Ser
NC_007323	pXO2	22995	C	T	T	T	T	T	T	T	T	T	T	T	T	T	T	GBAA_RS28090	-	hypothetical protein	missense_variant c.2141G>A p.Arg714Lys
NC_007323	pXO2	29385	A	A	A	A	A	A	T	T	T	T	T	T	T	T	T	GBAA_RS28130	-	hypothetical protein	missense_variant c.38T>A p.Ile13Asn
NC_007323	pXO2	30655	G	G	G	G	G	G	T	T	T	T	T	T	T	T	T	IGR	-	-	-
NC_007323	pXO2	37496	T	C	C	C	C	C	C	C	C	C	C	C	C	C	C	GBAA_RS28165	-	amidase	missense_variant c.80T>C p.Leu27Ser
NC_007323	pXO2	37950	C	T	T	T	T	T	T	T	T	T	T	T	T	T	T	GBAA_RS28165	-	amidase	synonymous_variant c.534C>T p.Gly178Gly
NC_007323	pXO2	38347	G	A	A	A	A	A	A	A	A	A	A	A	A	A	A	GBAA_RS28165	-	amidase	missense_variant c.931G>A p.Gly311Ser
NC_007323	pXO2	41262	A	C	C	C	C	C	A	A	A	A	A	A	A	A	A	GBAA_RS31170	-	hypothetical protein	missense_variant c.148A>C p.Thr50Pro
NC_007323	pXO2	44294	C	A	A	A	A	C	C	C	C	C	C	C	C	C	C	GBAA_RS28205	-	TetR family transcriptional regulator	missense_variant c.316C>A p.Leu106Ile
NC_007323	pXO2	46530	C	A	C	C	C	C	C	C	C	C	C	C	C	C	C	IGR	-	-	-
NC_007323	pXO2	46834	G	G	G	G	G	G	A	A	A	A	A	A	A	A	A	GBAA_RS28215	-	undecaprenyl-diphosphatase	missense_variant c.146C>T p.Pro49Leu
NC_007323	pXO2	47129	T	T	T	T	T	T	C	C	C	C	C	C	C	C	C	GBAA_RS31175	-	UDP-diphosphatase	synonymous_variant c.15A>G p.Gln5Gln
NC_007323	pXO2	57031	C	C	C	C	C	C	T	T	T	T	T	T	T	T	T	IGR	-	-	-
NC_007323	pXO2	57326	A	G	G	G	G	G	G	G	G	G	G	G	G	G	G	GBAA_RS31180	-	hypothetical protein	synonymous_variant c.168T>C p.Ile56Ile
NC_007323	pXO2	61771	T	C	C	C	C	C	C	C	C	C	C	C	C	C	C	GBAA_RS28280	-	hypothetical protein	missense_variant c.452A>G p.Tyr151Cys
NC_007323	pXO2	64708	T	C	C	C	C	C	T	T	T	T	T	T	T	T	T	IGR	-	-	-
NC_007323	pXO2	66865	C	A	A	A	A	A	A	A	A	A	A	A	A	A	A	IGR	-	-	-
NC_007323	pXO2	72925	T	G	G	G	G	G	G	G	G	G	G	G	G	G	G	IGR	-	-	-
NC_007323	pXO2	73543	A	A	A	A	A	A	A	A	G	G	G	G	G	G	G	GBAA_RS28340	-	peptide ABC transporter substrate-binding protein	missense_variant c.1039T>C p.Trp347Arg
NC_007323	pXO2	80793	G	A	A	A	A	A	A	A	A	A	A	A	A	A	A	GBAA_RS28380	-	hypothetical protein	synonymous_variant c.237C>T p.Ser79Ser
NC_007323	pXO2	81642	T	T	T	T	T	T	C	C	C	C	C	C	C	C	C	IGR	-	-	-
NC_007530	Chromosome	7764	T	T	T	T	T	T	C	C	C	C	C	C	C	C	C	GBAA_RS00040	-	DNA gyrase subunit A	synonymous_variant c.1170T>C p.His390His
NC_007530	Chromosome	17294	T	C	C	C	C	C	C	C	C	C	C	C	C	C	C	GBAA_RS00080	-	D-alanyl-D-alanine carboxypeptidase	missense_variant c.259T>C p.Phe87Leu
NC_007530	Chromosome	28847	A	G	G	G	G	G	A	A	A	A	A	A	A	A	A	GBAA_RS00155	-	pro-sigmaK processing inhibitor BofA	synonymous_variant c.234A>G p.Ala78Ala
NC_007530	Chromosome	38629	G	G	G	G	G	G	A	A	A	A	A	A	A	A	A	GBAA_RS00210	-	DNA replication initiation control protein YabA	missense_variant c.175G>A p.Glu59Lys
NC_007530	Chromosome	45072	A	A	A	A	G	A	A	A	A	A	A	A	A	A	A	GBAA_RS00245	rnmV	ribonuclease M5	synonymous_variant c.345A>G p.Glu115Glu
NC_007530	Chromosome	48627	T	C	C	C	C	C	C	C	C	C	C	C	C	C	C	GBAA_RS00270	-	4-(cytidine 5'-diphospho)-2-C-methyl-D-erythritol kinase	missense_variant c.536T>C p.Phe179Ser
NC_007530	Chromosome	65952	C	T	T	T	T	T	C	C	C	C	C	C	C	C	C	GBAA_RS00365	spoIIE	stage II sporulation protein E	synonymous_variant c.1065C>T p.Ser355Ser
NC_007530	Chromosome	72369	A	G	G	G	G	G	A	A	A	A	A	A	A	A	A	GBAA_RS00385	-	type III pantothenate kinase	missense_variant c.641A>G p.Glu214Gly
NC_007530	Chromosome	81435	C	C	C	C	C	C	C	C	T	T	T	T	T	T	T	GBAA_RS00440	lysS	lysine--tRNA ligase	synonymous_variant c.885C>T p.Ile295Ile
NC_007530	Chromosome	88559	T	C	C	C	C	C	C	C	C	C	C	C	C	C	C	GBAA_RS00465	-	hypothetical protein	synonymous_variant c.510T>C p.Asn170Asn
NC_007530	Chromosome	93736	C	T	T	T	T	T	T	T	T	T	T	T	T	T	T	GBAA_RS00485	-	dna integrity scanning protein disa	synonymous_variant c.135C>T p.Asn45Asn
NC_007530	Chromosome	97988	A	G	G	G	G	G	G	G	G	G	G	G	G	G	G	GBAA_RS00505	-	glutamate--tRNA ligase	synonymous_variant c.666A>G p.Thr222Thr
NC_007530	Chromosome	115639	T	T	T	T	T	T	C	C	C	C	C	C	C	C	C	IGR	-	-	-
NC_007530	Chromosome	120599	C	C	C	C	C	C	T	T	T	T	T	T	T	T	T	IGR	-	-	-
NC_007530	Chromosome	125858	G	G	G	G	G	G	A	A	A	A	A	A	A	A	A	GBAA_RS00665	-	30S ribosomal protein S17	missense_variant c.116G>A p.Arg39His
NC_007530	Chromosome	129487	C	C	C	C	C	C	T	T	T	T	T	T	T	T	T	GBAA_RS00710	-	50S ribosomal protein L30	missense_variant c.5C>T p.Ala2Val
NC_007530	Chromosome	145252	A	A	A	A	A	A	G	G	G	G	G	G	G	G	G	GBAA_RS00825	-	hypothetical protein	missense_variant c.208A>G p.Thr70Ala
NC_007530	Chromosome	163027	A	G	G	G	G	G	G	G	G	G	G	G	G	G	G	GBAA_RS29375	-	hypothetical protein	missense_variant c.508A>G p.Thr170Ala
NC_007530	Chromosome	169558	G	G	G	G	G	G	A	A	A	A	A	A	A	A	A	GBAA_RS00960	-	DUF4256 domain-containing protein	missense_variant c.478G>A p.Asp160Asn
NC_007530	Chromosome	175992	C	T	T	T	T	T	T	T	T	T	T	T	T	T	T	GBAA_RS01000	-	methionine import ATP-binding protein MetN 1	missense_variant c.258G>A p.Met86Ile
NC_007530	Chromosome	182106	C	T	T	T	T	T	T	T	T	T	T	T	T	T	T	GBAA_RS01030	-	DUF3981 domain-containing protein	missense_variant c.778C>T p.Leu260Phe
NC_007530	Chromosome	194935	A	T	T	T	T	T	T	T	T	T	T	T	T	T	T	IGR	-	-	-
NC_007530	Chromosome	200666	C	C	C	C	C	C	T	T	T	T	T	T	T	T	T	GBAA_RS01125	-	glucose transporter GlcU	missense_variant c.70G>A p.Gly24Ser
NC_007530	Chromosome	200731	C	T	T	T	T	T	T	T	T	T	T	T	T	T	T	GBAA_RS01125	-	glucose transporter GlcU	missense_variant c.5G>A p.Gly2Asp
NC_007530	Chromosome	210058	C	C	C	C	C	C	C	T	C	C	C	C	C	C	C	GBAA_RS01195	-	YitT family protein	missense_variant c.736C>T p.Leu246Phe
NC_007530	Chromosome	213430	C	C	C	C	C	C	G	G	G	G	G	G	G	G	G	GBAA_RS01215	-	DUF2334 domain-containing protein	missense_variant c.484G>C p.Ala162Pro
NC_007530	Chromosome	225023	G	T	T	T	T	T	G	G	G	G	G	G	G	G	G	GBAA_RS01280	-	HAD family phosphatase	missense_variant c.782C>A p.Thr261Asn
NC_007530	Chromosome	229509	C	C	C	C	C	C	T	T	T	T	T	T	T	T	T	GBAA_RS01310	-	homogentisate 1,2-dioxygenase	missense_variant c.577C>T p.Pro193Ser
NC_007530	Chromosome	231495	A	G	G	G	G	G	G	G	G	G	G	G	G	G	G	GBAA_RS01315	-	MFS transporter	missense_variant c.1157A>G p.His386Arg
NC_007530	Chromosome	239100	T	T	T	T	T	T	C	C	C	C	C	C	C	C	C	GBAA_RS01355	-	outer membrane lipoprotein carrier protein LolA	missense_variant c.524T>C p.Val175Ala
NC_007530	Chromosome	243867	C	T	T	T	T	T	T	T	T	T	T	T	T	T	T	GBAA_RS01375	-	RNA-binding transcriptional accessory protein	missense_variant c.1976C>T p.Ala659Val
NC_007530	Chromosome	265308	C	T	T	T	T	T	C	C	C	C	C	C	C	C	C	GBAA_RS01535	-	sensor histidine kinase	missense_variant c.323C>T p.Ser108Phe
NC_007530	Chromosome	284672	T	C	T	T	T	T	T	T	T	T	T	T	T	T	T	GBAA_RS01595	-	23S ribosomal RNA	intragenic_variant n.284672T>C
NC_007530	Chromosome	304816	A	G	G	G	G	G	G	G	G	G	G	G	G	G	G	GBAA_RS01685	-	phosphoribosylformylglycinamidine cyclo-ligase	missense_variant c.893A>G p.Asn298Ser
NC_007530	Chromosome	323707	T	C	C	C	C	C	C	C	C	C	C	C	C	C	C	GBAA_RS01775	-	HXXEE domain-containing protein	missense_variant c.316A>G p.Thr106Ala
NC_007530	Chromosome	326350	T	G	G	G	G	G	G	G	G	G	G	G	G	G	G	GBAA_RS01795	-	hypothetical protein	stop_lost c.115T>G p.Ter39Glyext*?
NC_007530	Chromosome	333685	T	C	C	C	C	C	T	T	T	T	T	T	T	T	T	IGR	-	-	-
NC_007530	Chromosome	337241	T	C	C	C	C	C	C	C	C	C	C	C	C	C	C	GBAA_RS01845	-	aminopeptidase	missense_variant c.922A>G p.Lys308Glu
NC_007530	Chromosome	346609	C	C	C	C	C	C	G	G	G	G	G	G	G	G	G	IGR	-	-	-
NC_007530	Chromosome	352728	T	A	A	A	A	A	A	A	A	A	A	A	A	A	A	GBAA_RS01910	-	amidohydrolase	missense_variant c.293T>A p.Met98Lys
NC_007530	Chromosome	353084	A	A	A	A	A	A	G	G	G	G	G	G	G	G	G	GBAA_RS01910	-	amidohydrolase	missense_variant c.649A>G p.Met217Val
NC_007530	Chromosome	359536	G	A	A	A	A	A	A	A	A	A	A	A	A	A	A	GBAA_RS01940	-	fuculose phosphate aldolase	missense_variant c.146G>A p.Cys49Tyr
NC_007530	Chromosome	364452	A	A	A	A	A	A	G	G	G	G	G	G	G	G	G	GBAA_RS01960	-	Ferredoxin--NADP reductase 1	missense_variant c.1037A>G p.Gln346Arg
NC_007530	Chromosome	364527	T	G	G	G	G	G	G	G	G	G	G	G	G	G	G	GBAA_RS01965	-	hypothetical protein	missense_variant c.23T>G p.Val8Gly
NC_007530	Chromosome	377412	A	A	A	A	A	A	G	G	G	G	G	G	G	G	G	GBAA_RS02045	-	acyl-CoA desaturase	missense_variant c.551A>G p.Asn184Ser
NC_007530	Chromosome	384004	G	A	A	A	A	A	A	A	A	A	A	A	A	A	A	IGR	-	-	-
NC_007530	Chromosome	387637	G	A	A	A	A	A	A	A	A	A	A	A	A	A	A	GBAA_RS02090	-	endonuclease	missense_variant c.103G>A p.Val35Ile
NC_007530	Chromosome	388477	A	A	A	A	A	A	G	G	G	G	G	G	G	G	G	IGR	-	-	-
NC_007530	Chromosome	404435	G	C	C	C	C	C	C	C	C	C	C	C	C	C	C	IGR	-	-	-
NC_007530	Chromosome	405303	C	T	T	T	T	T	T	T	T	T	T	T	T	T	T	GBAA_RS02150	-	chitinase	missense_variant c.1346G>A p.Gly449Asp
NC_007530	Chromosome	410758	A	G	G	G	G	G	A	A	A	A	A	A	A	A	A	GBAA_RS02185	-	hypothetical protein	synonymous_variant c.255A>G p.Arg85Arg
NC_007530	Chromosome	422826	G	A	A	A	A	A	A	A	A	A	A	A	A	A	A	GBAA_RS02245	-	toxic anion resistance protein	synonymous_variant c.435G>A p.Ser145Ser
NC_007530	Chromosome	423635	T	C	C	C	C	C	C	C	C	C	C	C	C	C	C	GBAA_RS02250	-	cation-translocating P-type ATPase	missense_variant c.2542A>G p.Ile848Val
NC_007530	Chromosome	430502	C	C	C	C	C	C	T	T	T	T	T	T	T	T	T	GBAA_RS02275	-	heavy metal translocating P-type ATPase	missense_variant c.284G>A p.Gly95Asp
NC_007530	Chromosome	433277	G	T	T	T	T	T	T	T	T	T	T	T	T	T	T	GBAA_RS02290	lepB	signal peptidase I	missense_variant c.239G>T p.Gly80Val
NC_007530	Chromosome	441647	G	C	C	C	C	C	C	C	C	C	C	C	C	C	C	GBAA_RS02330	yfkAB	radical SAM/CxCxxxxC motif protein YfkAB	missense_variant c.335C>G p.Pro112Arg
NC_007530	Chromosome	451584	C	T	T	T	T	T	T	T	T	T	T	T	T	T	T	IGR	-	-	-
NC_007530	Chromosome	456825	A	G	G	G	G	G	A	A	A	A	A	A	A	A	A	GBAA_RS02445	-	nucleotide pyrophosphohydrolase	missense_variant c.29A>G p.Glu10Gly
NC_007530	Chromosome	477179	A	T	T	T	T	T	T	T	T	T	T	T	T	T	T	GBAA_RS02610	-	SGNH/GDSL hydrolase family protein	missense_variant c.549A>T p.Lys183Asn
NC_007530	Chromosome	487308	T	T	T	T	T	T	A	A	A	A	A	A	A	A	A	IGR	-	-	-
NC_007530	Chromosome	503707	T	C	C	C	C	C	T	T	T	T	T	T	T	T	T	GBAA_RS02755	pflB	formate C-acetyltransferase	missense_variant c.107T>C p.Val36Ala
NC_007530	Chromosome	515111	G	A	A	A	A	A	A	A	A	A	A	A	A	A	A	GBAA_RS02820	mutY	A/G-specific adenine glycosylase	synonymous_variant c.129G>A p.Ser43Ser
NC_007530	Chromosome	519537	C	T	T	T	T	T	T	T	T	T	T	T	T	T	T	GBAA_RS02845	-	ABC transporter ATP-binding protein	missense_variant c.1519C>T p.Pro507Ser
NC_007530	Chromosome	524729	C	C	C	C	C	C	T	T	T	T	T	T	T	T	T	GBAA_RS02855	-	glutamate synthase	missense_variant c.850G>A p.Val284Ile
NC_007530	Chromosome	551400	T	C	C	C	C	C	C	C	C	C	C	C	C	C	C	GBAA_RS03055	-	sporulation protein YhbH	synonymous_variant c.507T>C p.Gly169Gly
NC_007530	Chromosome	565044	C	C	C	C	C	C	T	T	T	T	T	T	T	T	T	GBAA_RS03090	-	methyl-accepting chemotaxis protein	missense_variant c.1136C>T p.Ala379Val
NC_007530	Chromosome	571123	C	T	T	T	T	T	T	T	T	T	T	T	T	T	T	IGR	-	-	-
NC_007530	Chromosome	571883	T	G	G	G	G	G	T	T	T	T	T	T	T	T	T	GBAA_RS03120	-	hypothetical protein	missense_variant c.644T>G p.Ile215Ser
NC_007530	Chromosome	580182	T	C	C	C	C	C	C	C	C	C	C	C	C	C	C	GBAA_RS03160	-	sensor histidine kinase	missense_variant c.889T>C p.Tyr297His
NC_007530	Chromosome	595491	T	C	C	C	C	C	C	C	C	C	C	C	C	C	C	GBAA_RS03225	-	DNA-binding response regulator	missense_variant c.469A>G p.Arg157Gly
NC_007530	Chromosome	610254	G	A	A	A	A	A	A	A	A	A	A	A	A	A	A	IGR	-	-	-
NC_007530	Chromosome	641697	C	T	T	T	T	T	C	C	C	C	C	C	C	C	C	GBAA_RS29515	-	hypothetical protein	synonymous_variant c.357C>T p.Tyr119Tyr
NC_007530	Chromosome	644625	T	C	C	C	C	C	C	C	C	C	C	C	C	C	C	GBAA_RS03430	-	arginine:ornithine antiporter	missense_variant c.944A>G p.Asp315Gly
NC_007530	Chromosome	645461	A	A	A	A	A	A	T	T	T	T	T	T	T	T	T	GBAA_RS03430	-	arginine:ornithine antiporter	synonymous_variant c.108T>A p.Gly36Gly
NC_007530	Chromosome	648730	C	C	C	C	C	C	T	T	T	T	T	T	T	T	T	GBAA_RS03445	treC	alpha,alpha-phosphotrehalase	missense_variant c.422C>T p.Ala141Val
NC_007530	Chromosome	665390	C	C	C	C	C	C	T	T	T	T	T	T	T	T	T	GBAA_RS03525	-	YafY family transcriptional regulator	missense_variant c.398G>A p.Arg133Lys
NC_007530	Chromosome	666458	A	A	A	A	A	A	G	G	G	G	G	G	G	G	G	GBAA_RS03535	-	sensor histidine kinase	missense_variant c.1363T>C p.Ser455Pro
NC_007530	Chromosome	668516	C	C	C	C	C	C	T	T	T	T	T	T	T	T	T	IGR	-	-	-
NC_007530	Chromosome	672382	G	G	G	G	G	G	A	A	A	A	A	A	A	A	A	IGR	-	-	-
NC_007530	Chromosome	675837	A	A	A	A	A	A	G	G	G	G	G	G	G	G	G	GBAA_RS03575	-	ABC transporter permease	synonymous_variant c.321T>C p.Ile107Ile
NC_007530	Chromosome	680253	A	A	A	A	A	A	G	G	G	G	G	G	G	G	G	GBAA_RS03595	-	MarR family transcriptional regulator	synonymous_variant c.243A>G p.Val81Val
NC_007530	Chromosome	692932	C	T	T	T	T	T	T	T	T	T	T	T	T	T	T	GBAA_RS03655	-	MFS transporter	synonymous_variant c.219G>A p.Gly73Gly
NC_007530	Chromosome	701044	T	C	C	C	C	C	C	C	C	C	C	C	C	C	C	GBAA_RS03695	-	hypothetical protein	missense_variant c.61T>C p.Cys21Arg
NC_007530	Chromosome	702761	G	C	C	C	C	C	C	C	C	C	C	C	C	C	C	GBAA_RS03705	-	methyl-accepting chemotaxis protein	missense_variant c.374C>G p.Ala125Gly
NC_007530	Chromosome	703190	A	G	G	G	G	G	G	G	G	G	G	G	G	G	G	IGR	-	-	-
NC_007530	Chromosome	718595	A	A	A	A	A	A	G	G	G	G	G	G	G	G	G	GBAA_RS03780	-	sodium:proton antiporter	missense_variant c.518T>C p.Met173Thr
NC_007530	Chromosome	719485	G	A	A	A	A	A	A	A	A	A	A	A	A	A	A	GBAA_RS03785	qoxD	cytochrome aa3 quinol oxidase subunit IV	missense_variant c.161C>T p.Ala54Val
NC_007530	Chromosome	732173	A	T	T	T	T	T	T	T	T	T	T	T	T	T	T	GBAA_RS03850	-	ferrous iron transporter B	missense_variant c.622T>A p.Leu208Met
NC_007530	Chromosome	732244	A	A	A	A	A	A	A	A	G	G	G	G	G	G	G	GBAA_RS03850	-	ferrous iron transporter B	missense_variant c.551T>C p.Met184Thr
NC_007530	Chromosome	740411	C	C	C	C	C	C	T	T	T	T	T	T	T	T	T	GBAA_RS03885	-	AcrB/AcrD/AcrF family protein	synonymous_variant c.432G>A p.Lys144Lys
NC_007530	Chromosome	746784	G	G	G	G	G	G	G	G	G	G	G	G	G	T	G	GBAA_RS03925	-	tRNA-Gly	intragenic_variant n.746784G>T

NC_007530	Chromosome	749442 G	G	G	G	G	G	A	A	A	A	A	A	A	A	A	GBAA_RS04005	-	hypothetical protein	synonymous_variant c.492G>A p.Lys164Lys
NC_007530	Chromosome	765499 A	C	C	C	C	C	C	C	C	C	C	C	C	C	C	GBAA_RS04100	kdpB	potassium-transporting ATPase subunit B	synonymous_variant c.1968A>C p.Ile656Ile
NC_007530	Chromosome	768418 C	T	T	T	T	T	C	C	C	C	C	C	C	C	C	GBAA_RS04115	-	MFS transporter	missense_variant c.223G>A p.Val75Ile
NC_007530	Chromosome	778110 G	T	T	T	T	T	T	T	T	T	T	T	T	T	T	IGR	-	-	-
NC_007530	Chromosome	778444 A	G	G	G	G	G	A	A	A	A	A	A	A	A	A	GBAA_RS04170	-	LacI family transcriptional regulator	synonymous_variant c.696T>C p.Asn232Asn
NC_007530	Chromosome	782434 C	C	C	C	C	C	T	T	T	T	T	T	T	T	T	GBAA_RS04200	-	spore gernimation protein GerB	missense_variant c.157G>A p.Ala53Thr
NC_007530	Chromosome	783005 A	T	T	T	T	T	A	A	A	A	A	A	A	A	A	GBAA_RS04205	-	Ger(x)C family spore germination protein	synonymous_variant c.681T>A p.Arg227Arg
NC_007530	Chromosome	788107 A	A	A	A	A	A	G	G	G	G	G	G	G	G	G	GBAA_RS04230	-	stage V sporulation protein R	missense_variant c.1292A>G p.Tyr431Cys
NC_007530	Chromosome	789298 G	A	A	A	A	A	A	A	A	A	A	A	A	A	A	GBAA_RS04235	-	alkaline ceramidase	synonymous_variant c.957G>A p.Leu319Leu
NC_007530	Chromosome	791840 G	C	C	C	C	C	C	C	C	C	C	C	C	C	C	GBAA_RS04255	cdr	CoA-disulfide reductase	synonymous_variant c.36G>C p.Ala12Ala
NC_007530	Chromosome	796747 G	G	G	G	G	G	A	A	A	A	A	A	A	A	A	IGR	-	-	-
NC_007530	Chromosome	796802 A	A	A	A	A	A	G	G	G	G	G	G	G	G	G	GBAA_RS04290	-	hypothetical protein	synonymous_variant c.42A>G p.Gly14Gly
NC_007530	Chromosome	802560 A	T	T	T	T	T	T	T	T	T	T	T	T	T	T	GBAA_RS04315	-	sodium:phosphate symporter	synonymous_variant c.297T>A p.Thr99Thr
NC_007530	Chromosome	807975 A	G	G	G	G	G	G	G	G	G	G	G	G	G	G	GBAA_RS04335	-	PTS lactose/cellobiose transporter subunit IIA	missense_variant c.58A>G p.Ile20Val
NC_007530	Chromosome	816468 C	C	C	C	C	C	T	T	T	T	T	T	T	T	T	GBAA_RS04380	-	TIGR02206 family membrane protein	missense_variant c.35C>T p.Pro12Leu
NC_007530	Chromosome	821008 G	A	A	A	A	A	A	A	A	A	A	A	A	A	A	GBAA_RS04400	-	hypothetical protein	synonymous_variant c.270C>T p.Ala90Ala
NC_007530	Chromosome	821712 A	A	A	A	A	A	G	G	G	G	G	G	G	G	G	GBAA_RS04410	-	hypothetical protein	synonymous_variant c.66T>C p.Gly22Gly
NC_007530	Chromosome	822097 A	G	G	G	G	G	A	A	A	A	A	A	A	A	A	GBAA_RS04415	-	lactoylglutathione lyase	missense_variant c.226A>G p.Ile76Val
NC_007530	Chromosome	826692 A	G	G	G	G	G	G	G	G	G	G	G	G	G	G	IGR	-	-	-
NC_007530	Chromosome	832812 C	C	C	C	C	C	T	T	T	T	T	T	T	T	T	GBAA_RS04480	-	hypothetical protein	synonymous_variant c.145C>T p.Leu49Leu
NC_007530	Chromosome	835926 C	T	T	T	T	T	C	C	C	C	C	C	C	C	C	GBAA_RS04495	-	permease	synonymous_variant c.987C>T p.Leu329Leu
NC_007530	Chromosome	842022 A	A	A	A	A	A	A	G	A	A	A	A	A	A	A	GBAA_RS04540	-	QacE family quaternary ammonium compound efflux SMR transporter	missense_variant c.64A>G p.Met22Val
NC_007530	Chromosome	846134 A	G	G	G	G	G	A	A	A	A	A	A	A	A	A	GBAA_RS04570	-	SgrR family transcriptional regulator	missense_variant c.1721T>C p.Ile574Thr
NC_007530	Chromosome	848995 G	T	T	T	T	T	T	T	T	T	T	T	T	T	T	GBAA_RS04575	-	MFS transporter	missense_variant c.1036G>T p.Asp346Tyr
NC_007530	Chromosome	850519 T	C	C	C	C	C	C	C	C	C	C	C	C	C	C	GBAA_RS04590	-	catalase HPII	missense_variant c.1708A>G p.Asn570Asp
NC_007530	Chromosome	851884 C	T	T	T	T	T	T	T	T	T	T	T	T	T	T	GBAA_RS04590	-	catalase HPII	missense_variant c.343G>A p.Ala115Thr
NC_007530	Chromosome	861557 A	C	C	C	C	C	A	A	A	A	A	A	A	A	A	GBAA_RS04630	-	ABC transporter ATP-binding protein	synonymous_variant c.18A>C p.Arg6Arg
NC_007530	Chromosome	867050 A	C	C	C	C	C	C	C	C	C	C	C	C	C	C	GBAA_RS04660	-	hypothetical protein	missense_variant c.1345T>G p.Tyr449Asp
NC_007530	Chromosome	880346 G	G	G	G	G	G	A	A	A	A	A	A	A	A	A	GBAA_RS04715	-	hypothetical protein	synonymous_variant c.2496G>A p.Pro832Pro
NC_007530	Chromosome	880994 C	T	T	T	T	T	T	T	T	T	T	T	T	T	T	GBAA_RS29550	-	N-acetylmuramoyl-L-alanine amidase	missense_variant c.871G>A p.Val291Met
NC_007530	Chromosome	882646 G	A	A	A	A	A	G	G	G	G	G	G	G	G	G	IGR	-	-	-
NC_007530	Chromosome	883821 T	G	G	G	G	G	T	T	T	T	T	T	T	T	T	GBAA_RS04725	-	coproporphyrinogen III oxidase	missense_variant c.1014T>G p.Ile338Met
NC_007530	Chromosome	886888 T	G	G	G	G	G	T	T	T	T	T	T	T	T	T	GBAA_RS04740	-	SulP family inorganic anion transporter	synonymous_variant c.33T>G p.Ala11Ala
NC_007530	Chromosome	889159 A	A	A	A	A	A	G	G	G	G	G	G	G	G	G	IGR	-	-	-
NC_007530	Chromosome	889508 A	A	A	A	A	A	A	G	A	A	A	A	A	A	A	GBAA_RS04750	-	hypothetical protein	synonymous_variant c.219A>G p.Ala73Ala
NC_007530	Chromosome	889545 A	G	G	G	G	G	G	G	G	G	G	G	G	G	G	GBAA_RS04750	-	hypothetical protein	missense_variant c.256A>G p.Lys86Glu
NC_007530	Chromosome	900902 T	C	C	C	C	C	T	T	T	T	T	T	T	T	T	GBAA_RS04780	-	S-layer protein EA1	synonymous_variant c.988T>C p.Leu330Leu
NC_007530	Chromosome	911260 C	T	T	T	T	T	C	C	C	C	C	C	C	C	C	GBAA_RS04825	-	hypothetical protein	missense_variant c.79G>A p.Ala27Thr
NC_007530	Chromosome	912520 G	A	A	A	A	A	A	A	A	A	A	A	A	A	A	IGR	-	-	-
NC_007530	Chromosome	915310 G	A	A	A	A	A	G	G	G	G	G	G	G	G	G	GBAA_RS04840	-	RNA methyltransferase	missense_variant c.835G>A p.Val279Ile
NC_007530	Chromosome	916216 C	C	C	C	C	C	T	T	T	T	T	T	T	T	T	GBAA_RS04850	-	proline racemase	missense_variant c.205C>T p.Pro69Ser
NC_007530	Chromosome	932591 T	C	C	C	C	C	T	T	T	T	T	T	T	T	T	IGR	-	-	-
NC_007530	Chromosome	947760 T	C	C	C	C	C	C	C	C	C	C	C	C	C	C	GBAA_RS05010	-	DUF4064 domain-containing protein	synonymous_variant c.138T>C p.Gly46Gly
NC_007530	Chromosome	949149 C	C	C	C	C	C	C	T	C	C	C	C	C	C	C	IGR	-	-	-
NC_007530	Chromosome	953671 A	A	A	A	A	A	G	G	G	G	G	G	G	G	G	GBAA_RS05045	-	hypothetical protein	synonymous_variant c.201T>C p.Ile67Ile
NC_007530	Chromosome	956449 T	C	C	C	C	C	C	C	C	C	C	C	C	C	C	GBAA_RS05065	-	carboxylic ester hydrolase	synonymous_variant c.303A>G p.Leu101Leu
NC_007530	Chromosome	958994 G	G	G	G	G	G	A	A	A	A	A	A	A	A	A	GBAA_RS05085	-	MxaA domain-containing protein	synonymous_variant c.1683C>T p.Phe561Phe
NC_007530	Chromosome	976554 A	A	A	A	A	A	A	A	G	G	G	G	G	G	G	IGR	-	-	-
NC_007530	Chromosome	983452 A	G	G	G	G	G	A	A	A	A	A	A	A	A	A	GBAA_RS05215	-	lipoprotein	missense_variant c.85T>C p.Ser29Pro
NC_007530	Chromosome	984681 C	C	C	C	C	C	C	C	T	T	T	T	T	T	T	GBAA_RS05230	-	GlsB/YeaQ/YmgE family stress response membrane protein	synonymous_variant c.216C>T p.Ile72Ile
NC_007530	Chromosome	1005708 C	T	T	T	T	T	T	T	T	T	T	T	T	T	T	GBAA_RS05360	-	MFS transporter	missense_variant c.206C>T p.Ala69Val
NC_007530	Chromosome	1014487 G	A	A	A	A	A	A	A	A	A	A	A	A	A	A	IGR	-	-	-
NC_007530	Chromosome	1016668 T	C	C	C	C	C	C	C	C	C	C	C	C	C	C	GBAA_RS05410	glpK	glycerol kinase	missense_variant c.1160T>C p.Ile387Thr
NC_007530	Chromosome	1019638 T	T	T	T	T	T	C	C	C	C	C	C	C	C	C	GBAA_RS05425	-	DUF2283 domain-containing protein	missense_variant c.14T>C p.Ile5Thr
NC_007530	Chromosome	1045124 G	A	A	A	A	A	G	G	G	G	G	G	G	G	G	GBAA_RS05575	-	MerR family transcriptional regulator	missense_variant c.395G>A p.Arg132His
NC_007530	Chromosome	1050290 G	A	A	A	A	A	A	A	A	A	A	A	A	A	A	GBAA_RS05600	-	penicillin-binding protein	missense_variant c.1414G>A p.Val472Ile
NC_007530	Chromosome	1051536 G	A	A	A	A	A	G	G	G	G	G	G	G	G	G	GBAA_RS05605	-	uroporphyrinogen decarboxylase	synonymous_variant c.363G>A p.Pro121Pro
NC_007530	Chromosome	1051640 G	A	A	A	A	A	A	A	A	A	A	A	A	A	A	GBAA_RS05605	-	uroporphyrinogen decarboxylase	missense_variant c.467G>A p.Gly156Asp
NC_007530	Chromosome	1068697 G	G	G	G	G	G	A	A	A	A	A	A	A	A	A	GBAA_RS05680	-	LacI family transcriptional regulator	missense_variant c.649G>A p.Ala217Thr
NC_007530	Chromosome	1074185 T	C	C	C	C	C	C	C	C	C	C	C	C	C	C	GBAA_RS05715	-	hypothetical protein	missense_variant c.212T>C p.Leu71Pro
NC_007530	Chromosome	1082792 T	C	C	C	C	C	C	C	C	C	C	C	C	C	C	GBAA_RS05730	-	ribonuclease E inhibitor RraB	missense_variant c.254T>C p.Ile85Thr
NC_007530	Chromosome	1086203 C	T	T	T	T	T	T	T	T	T	T	T	T	T	T	GBAA_RS05765	-	hypothetical protein	missense_variant c.143G>A p.Gly48Asp
NC_007530	Chromosome	1089080 T	C	C	C	C	C	C	C	C	C	C	C	C	C	C	GBAA_RS05785	-	HD domain-containing protein	missense_variant c.929T>C p.Val310Ala
NC_007530	Chromosome	1099933 C	T	T	T	T	T	T	T	T	T	T	T	T	T	T	GBAA_RS05850	-	methyl-accepting chemotaxis protein	stop_gained c.1105C>T p.Gln369*
NC_007530	Chromosome	1101562 T	C	C	C	C	C	C	C	C	C	C	C	C	C	C	GBAA_RS05855	-	EamA/RhaT family transporter	missense_variant c.794T>C p.Leu265Pro
NC_007530	Chromosome	1102136 A	G	G	G	G	G	G	G	G	G	G	G	G	G	G	GBAA_RS05860	-	S-layer protein	missense_variant c.382A>G p.Ile128Val
NC_007530	Chromosome	1114756 C	C	C	C	C	C	T	T	T	T	T	T	T	T	T	GBAA_RS05920	addB	ATP-dependent helicase/deoxyribonuclease subunit B	missense_variant c.2618C>T p.Pro873Leu
NC_007530	Chromosome	1120096 C	T	T	T	T	T	T	T	T	T	T	T	T	T	T	GBAA_RS05940	-	spore germination protein GerPE	missense_variant c.263G>A p.Ser88Asn
NC_007530	Chromosome	1125455 G	A	A	A	A	A	A	A	A	A	A	A	A	A	A	GBAA_RS05990	-	DUF2777 domain-containing protein	synonymous_variant c.411C>T p.Cys137Cys
NC_007530	Chromosome	1128059 C	A	A	A	A	A	A	A	A	A	A	A	A	A	A	IGR	-	-	-
NC_007530	Chromosome	1130230 C	C	C	C	C	C	C	C	T	T	T	T	T	T	T	GBAA_RS06005	-	catalase	missense_variant c.1072C>T p.Pro358Ser
NC_007530	Chromosome	1131765 C	T	T	T	T	T	T	T	T	T	T	T	T	T	T	GBAA_RS06010	-	ammonia channel protein	missense_variant c.368G>A p.Arg123Gln
NC_007530	Chromosome	1131906 T	C	C	C	C	C	C	C	C	C	C	C	C	C	C	GBAA_RS06010	-	ammonia channel protein	stop_lost c.227A>G p.Ter76Trpext*?
NC_007530	Chromosome	1133580 C	C	C	C	C	C	T	T	T	T	T	T	T	T	T	GBAA_RS06020	-	alpha-amylase	synonymous_variant c.1236C>T p.Ser412Ser
NC_007530	Chromosome	1134120 C	T	T	T	T	T	T	T	T	T	T	T	T	T	T	GBAA_RS06025	-	XRE family transcriptional regulator	missense_variant c.224C>T p.Thr75Ile
NC_007530	Chromosome	1134135 G	T	T	T	T	T	G	G	G	G	G	G	G	G	G	GBAA_RS06025	-	XRE family transcriptional regulator	missense_variant c.239G>T p.Cys80Phe
NC_007530	Chromosome	1142308 G	A	A	A	A	A	A	A	A	A	A	A	A	A	A	GBAA_RS06085	clpB	chaperone protein ClpB	synonymous_variant c.384G>A p.Leu128Leu
NC_007530	Chromosome	1143094 G	A	A	A	A	A	G	G	G	G	G	G	G	G	G	GBAA_RS06085	clpB	chaperone protein ClpB	synonymous_variant c.1170G>A p.Pro390Pro
NC_007530	Chromosome	1146622 T	C	C	C	C	C	C	C	C	C	C	C	C	C	C	GBAA_RS06105	-	competence protein ComG	missense_variant c.35T>C p.Val12Ala
NC_007530	Chromosome	1149985 C	T	T	T	T	T	C	C	C	C	C	C	C	C	C	GBAA_RS06120	-	hypothetical protein	synonymous_variant c.522C>T p.Asn174Asn
NC_007530	Chromosome	1154308 A	A	A	A	A	A	G	G	G	G	G	G	G	G	G	GBAA_RS06140	-	peptide ABC transporter substrate-binding protein	synonymous_variant c.801A>G p.Ala267Ala
NC_007530	Chromosome	1165573 C	C	C	C	C	C	T	T	T	T	T	T	T	T	T	GBAA_RS06195	-	adaptor protein MecA	missense_variant c.529C>T p.His177Tyr
NC_007530	Chromosome	1174146 A	A	A	A	A	A	G	G	G	G	G	G	G	G	G	GBAA_RS06240	-	GTP pyrophosphokinase	synonymous_variant c.489A>G p.Gly163Gly
NC_007530	Chromosome	1174440 T	C	C	C	C	C	T	T	T	T	T	T	T	T	T	GBAA_RS06245	-	NAD(+) kinase	synonymous_variant c.126T>C p.Val42Val
NC_007530	Chromosome	1181950 A	G	G	G	G	G	G	G	G	G	G	G	G	G	G	GBAA_RS06280	-	macrocin O-methyltransferase	missense_variant c.448T>C p.Ser150Pro
NC_007530	Chromosome	1183147 C	C	C	C	T	T	C	T	C	T	C	C	C	T	C	GBAA_RS06285	-	hypothetical protein	synonymous_variant c.519_522delGCCGinsACCA p.175
NC_007530	Chromosome	1183150 C	C	C	C	T	T	C	T	C	T	C	C	C	T	C	GBAA_RS06285	-	hypothetical protein	synonymous_variant c.519_522delGCCGinsACCA p.175
NC_007530	Chromosome	1183465 C	T	T	T	T	T	T	T	T	T	T	T	T	C	T	GBAA_RS06285	-	hypothetical protein	synonymous_variant c.204G>A p.Gly68Gly
NC_007530	Chromosome	1200881 C	T	T	T	T	T	T	T	T	T	T	T	T	T	T	GBAA_RS06400	putP	sodium/proline symporter PutP	synonymous_variant c.1410G>A p.Leu470Leu
NC_007530	Chromosome	1211363 C	T	T	T	T	T	C	C	C	C	C	C	C	C	C	GBAA_RS06450	-	L-lactate permease	stop_gained c.1273C>T p.Gln425*
NC_007530	Chromosome	1214045 C	T	T	T	T	T	C	C	C	C	C	C	C	C	C	GBAA_RS06465	-	zinc ribbon domain-containing protein	synonymous_variant c.636C>T p.Val212Val
NC_007530	Chromosome	1214691 C	C	C	C	C	C	T	T	T	T	T	T	T	T	T	GBAA_RS06470	-	hypothetical protein	synonymous_variant c.285G>A p.Ala95Ala
NC_007530	Chromosome	1218569 C	T	T	T	T	T	T	T	T	T	T	T	T	T	T	GBAA_RS06490	-	NADH oxidase	synonymous_variant c.660G>A p.Val220Val
NC_007530	Chromosome	1219826 C	T	T	T	T	T	C	C	C	C	C	C	C	C	C	GBAA_RS06495	-	SagD family biosynthesis docking scaffold protein	missense_variant c.1369G>A p.Ala457Thr
NC_007530	Chromosome	1222395 C	C	C	C	C	C	T	T	T	T	T	T	T	T	T	GBAA_RS06500	-	putative thiazole-containing bacteriocin maturation protein	missense_variant c.716G>A p.Cys239Tyr
NC_007530	Chromosome	1223198 C	T	T	T	T	T	T	T	T	T	T	T	T	T	T	IGR	-	-	-
NC_007530	Chromosome	1224912 T	C	C	C	C	C	C	C	C	C	C	C	C	C	C	GBAA_RS06510	-	2-oxoglutarate dehydrogenase subunit E1	synonymous_variant c.2583A>G p.Glu861Glu
NC_007530	Chromosome	1228266 T	C	C	C	C	C	C	C	C	C	C	C	C	C	C	IGR	-	-	-
NC_007530	Chromosome	1240556 C	T	T	T	T	T	C	C	C	C	C	C	C	C	C	GBAA_RS06620	-	peptidase M6	missense_variant c.116C>T p.Ala39Val
NC_007530	Chromosome	1246856 C	T	T	T	T	T	C	C	C	C	C	C	C	C	C	GBAA_RS06640	-	spermidine/putrescine ABC transporter permease	missense_variant c.416C>T p.Ser139Leu
NC_007530	Chromosome	1252476 T	C	C	C	C	C	C	C	C	C	C	C	C	C	C	GBAA_RS06665	-	pseudouridine synthase	synonymous_variant c.534T>C p.Gly178Gly
NC_007530	Chromosome	1254326 T	C	C	C	C	C	C	C	C	C	C	C	C	C	C	GBAA_RS06675	-	serine protease	missense_variant c.106A>G p.Ile36Val
NC_007530	Chromosome	1285586 C	T	T	T	T	T	T	T	T	T	T	T	T	T	T	GBAA_RS06835	-	saccharopine dehydrogenase	stop_gained c.838C>T p.Gln280*
NC_007530	Chromosome	1290377 T	C	C	C	C	C	C	C	C	C	C	C	C	C	C	IGR	-	-	-
NC_007530	Chromosome	1297094 C	C	C	C	C	C	T	T	T	T	T	T	T	T	T	GBAA_RS06890	-	DinB family protein	synonymous_variant c.381C>T p.Ser127Ser
NC_007530	Chromosome	1301762 T	C	C	C	C	C	C	C	C	C	C	C	C	C	C	IGR	-	-	-
NC_007530	Chromosome	1303210 G	A	A	A	A	A	A	A	A	A	A	A	A	A	A	GBAA_RS06940	-	N-acetyltransferase	synonymous_variant c.162C>T p.Tyr54Tyr
NC_007530	Chromosome	1313016 T	C	C	C	C	C	C	C	C	C	C	C	C	C	C	IGR	-	-	-
NC_007530	Chromosome	1328446 C	C	C	C	C	C	C	T	C	C	C	C	C	C	C	GBAA_RS29730	-	hypothetical protein	synonymous_variant c.66C>T p.Asn22Asn
NC_007530	Chromosome	1331056 C	C	C	C	C	C	T	C	C	C	C	C	C	C	C	GBAA_RS07130	-	thioredoxin family protein	synonymous_variant c.318G>A p.Ala106Ala
NC_007530	Chromosome	1332255 A	T	T	T	T	T	T	T	T	T	T	T	T	T	T	GBAA_RS07140	-	MarR family transcriptional regulator	missense_variant c.198A>T p.Lys66Asn
NC_007530	Chromosome	1352945 G	A	A	A	A	A	A	A	A	A	A	A	A	A	A	GBAA_RS07255	hisH	imidazole glycerol phosphate synthase subunit HisH	synonymous_variant c.591G>A p.Gly197Gly
NC_007530	Chromosome	1361414 A	G	G	G	G	G	A	A	A	A	A	A	A	A	A	GBAA_RS07310	lysA	diaminopimelate decarboxylase	missense_variant c.67A>G p.Thr23Ala
NC_007530	Chromosome	1361756 G	A	A	A	A	A	G	G	G	G	G	G	G	G	G	GBAA_RS07310	lysA	diaminopimelate decarboxylase	missense_variant c.409G>A p.Glu137Lys
NC_007530	Chromosome	1362990 G	A	A	A	A	A	A	A	A	A	A	A	A	A	A	GBAA_RS07315	-	DUF2292 domain-containing protein	synonymous_variant c.30G>A p.Val10Val
NC_007530	Chromosome	1373031 G	A	A	A	A	A	G	G	G	G	G	G	G	G	G	IGR	-	-	-
NC_007530	Chromosome	1373220 A	A	A	A	A	A	G	G	G	G	G	G	G	G	G	IGR	-	-	-
NC_007530	Chromosome	1373534 A	G	G	G	G	G	G	G	G	G	G	G	G	G	G	GBAA_RS07375	-	glutamate/aspartate:proton symporter GltP	synonymous_variant c.39A>G p.Gly13Gly
NC_007530	Chromosome	1399271 C	T	T	T	T	T	T	T	T	T	T	T	T	T	T	GBAA_RS07515	-	DUF3924 domain-containing protein	synonymous_variant c.186G>A p.Glu62Glu
NC_007530	Chromosome	1404196 G	A	A	A	A	A	A	A	A	A	A	A	A	A	A	GBAA_RS07535	-	transposase	missense_variant c.947G>A p.Cys316Tyr
NC_007530	Chromosome	1411015 A	G	G	G	G	G	G	G	G	G	G	G	G	G	G	GBAA_RS07565	-	D-alanyl-D-alanine carboxypeptidase	missense_variant c.433A>G p.Arg145Gly
NC_007530	Chromosome	1414756 A	A	A	A	A	A	G	G	G	G	G	G	G	G	G	GBAA_RS07590	-	cytochrome c biogenesis protein	missense_variant c.178A>G p.Ser60Gly
NC_007530	Chromosome	1423420 A	G	G	G	G	G	G	G	G	G	G	G	G	G	G	GBAA_RS07625	-	riboflavin transporter FmnP	synonymous_variant c.432A>G p.Pro144Pro
NC_007530	Chromosome	1427847 G	C	C	C	C	C	C	C	C	C	C	C	C	C	C	GBAA_RS07650	-	peptidase M23	missense_variant c.446G>C p.Arg149Pro
NC_007530	Chromosome	1431379 G	G	G	G	G	G	A	A	A	A	A	A	A	A	A	GBAA_RS07670	-	Glu/Leu/Phe/Val dehydrogenase	missense_variant c.827G>A p.Arg276His
NC_007530	Chromosome	1431962 A	G	G	G	G	G	G	G	G	G	G	G	G	G	G	GBAA_RS07675	-	hypothetical protein	synonymous_variant c.45A>G p.Lys15Lys
NC_007530	Chromosome	1442759 C	C	C	C	C	C	T	T	T	T	T	T	T	T	T	GBAA_RS07745	-	glycerol-3-phosphate dehydrogenase (NAD(P)(+))	missense_variant c.188C>T p.Ser63Phe
NC_007530	Chromosome	1446160 C	T	T	T	T	T	C	C	C	C	C	C	C	C	C	GBAA_RS07765	spoIVA	stage IV sporulation protein A	synonymous_variant c.708C>T p.Phe236Phe
NC_007530	Chromosome	1448642 T	G	G	G	G	G	G	G	G	G	G	G	G	G	G	GBAA_RS07780	-	heptaprenyl diphosphate synthase subunit I	missense_variant c.190T>G p.Ser64Ala
NC_007530	Chromosome	1449693 T	C	C	C	C	C	C	C	C	C	C	C	C	C	C	GBAA_RS07785	-	demethylmenaquinone methyltransferase	synonymous_variant c.507T>C p.Tyr169Tyr
NC_007530	Chromosome	1451547 A	G	G	G	G	G	G	G	G	G	G	G	G	G	G	IGR	-	-	-
NC_007530	Chromosome	1462427 C	C	C	C	C	C	C	T	C	C	C	C	C	C	C	GBAA_RS07855	ypjB	sporulation protein YpjB	missense_variant c.716C>T p.Thr239Ile
NC_007530	Chromosome	1465199 A	G	G	G	G	G	G	G	G	G	G	G	G	G	G	GBAA_RS07875	-	hypothetical protein	synonymous_variant c.144A>G p.Thr48Thr
NC_007530	Chromosome	1479737 C	T	T	T	T	T	T	T	T	T	T	T	T	T	T	GBAA_RS07960	-	DNA replication protein DnaD	missense_variant c.685C>T p.Leu229Phe
NC_007530	Chromosome	1487756 G	G	G	G	G	G	A	A	A	A	A	A	A	A	A	GBAA_RS08010	-	spore coat protein D	synonymous_variant c.138G>A p.Thr46Thr
NC_007530	Chromosome	1493280 C	T	T	T	T	T	T	T	T	T	T	T	T	T	T	IGR	-	-	-
NC_007530	Chromosome	1512696 G	C	C	C	C	C	G	G	G	G	G	G	G	G	G	GBAA_RS08125	-	5'-3' exonuclease	missense_variant c.809G>C p.Arg270Pro
NC_007530	Chromosome	1515437 A	G	G	G	G	G	A	A	A	A	A	A	A	A	A	GBAA_RS08140	-	capsule biosynthesis protein CapK	missense_variant c.656A>G p.His219Arg
NC_007530	Chromosome	1523784 T	T	T	T	T	T	A	A	A	A	A	A	A	A	A	GBAA_RS08180	-	cell surface protein	missense_variant c.14330A>T p.Asn4777Ile
NC_007530	Chromosome	1527320 C	T	T	T	T	T	C	C	C	C	C	C	C	C	C	GBAA_RS08180	-	cell surface protein	synonymous_variant c.10794G>A p.Leu3598Leu
NC_007530	Chromosome	1531733 G	A	A	A	A	A	A	A	A	A	A	A	A	A	A	GBAA_RS08180	-	cell surface protein	synonymous_variant c.6381C>T p.Ser2127Ser
NC_007530	Chromosome	1532674 C	C	C	C	C	C	T	T	T	T	T	T	T	T	T	GBAA_RS08180	-	cell surface protein	missense_variant c.5440G>A p.Ala1814Thr
NC_007530	Chromosome	1534187 C	T	T	T	T	T	C	C	C	C	C	C	C	C	C	GBAA_RS08180	-	cell surface protein	synonymous_variant c.3927G>A p.Thr1309Thr
NC_007530	Chromosome	1536404 A	G	G	G	G	G	A	A	A	A	A	A	A	A	A	GBAA_RS08180	-	cell surface protein	synonymous_variant c.1710T>C p.Val570Val
NC_007530	Chromosome	1542573 G	A	A	A	A	A	A	A	A	A	A	A	A	A	A	GBAA_RS08215	-	QueT transporter family protein	initiator_codon_variant c.1G>A p.Val1?
NC_007530	Chromosome	1550746 A	G	G	G	G	G	G	G	G	G	G	G	G	G	G	GBAA_RS08270	-	aspartate aminotransferase family protein	missense_variant c.1008A>G p.Ile336Met
NC_007530	Chromosome	1560779 T	C	C	C	C	C	C	C	C	C	C	C	C	C	C	GBAA_RS08325	-	sulfite exporter TauE/SafE family protein	missense_variant c.710A>G p.Asn237Ser
NC_007530	Chromosome	1570190 G	A	A	A	A	A	A	A	A	A	A	A	A	A	A	GBAA_RS08385	-	chemotaxis protein CheA	missense_variant c.505G>A p.Glu169Lys
NC_007530	Chromosome	1571826 A	A	A	A	A	A	G	G	G	G	G	G	G	G	G	GBAA_RS08390	-	flagellar motor switch protein FliN	missense_variant c.561A>G p.Ile187Met
NC_007530	Chromosome	1572965 T	C	C	C	C	C	C	C	C	C	C	C	C	C	C	GBAA_RS08395	-	hypothetical protein	synonymous_variant c.30T>C p.Ser10Ser
NC_007530	Chromosome	1584331 C	T	T	T	T	T	T	T	T	T	T	T	T	T	T	GBAA_RS08470	-	flagellar M-ring protein FliF	missense_variant c.911C>T p.Ala304Val
NC_007530	Chromosome	1587896 A	A	A	A	A	A	G	G	G	G	G	G	G	G	G	GBAA_RS08490	fliI	flagellum-specific ATP synthase	synonymous_variant c.1218A>G p.Val406Val
NC_007530	Chromosome	1588831 G	A	A	A	A	A	G	G	G	G	G	G	G	G	G	GBAA_RS08505	-	flagellar hook-length control protein FliK	missense_variant c.34G>A p.Glu12Lys
NC_007530	Chromosome	1601152 A	A	A	A	A	A	G	G	G	G	G	G	G	G	G	GBAA_RS29825	-	IS3 family transposase	missense_variant c.877A>G p.Thr293Ala
NC_007530	Chromosome	1613208 C	C	C	C	C	C	C	C	C	C	C	T	C	C	C	GBAA_RS08640	-	flagellar type III secretion system protein FlhB	synonymous_variant c.466C>T p.Leu156Leu
NC_007530	Chromosome	1616347 T	C	C	C	C	C	C	C	C	C	C	C	C	C	C	GBAA_RS08650	flhF	flagellar biosynthesis protein FlhF	stop_lost c.454T>C p.Ter152Glnext*?
NC_007530	Chromosome	1627523 T	C	C	C	C	C	C	C	C	C	C	C	C	C	C	GBAA_RS08730	-	LysR family transcriptional regulator	missense_variant c.424A>G p.Asn142Asp
NC_007530	Chromosome	1631941 G	T	T	T	T	T	T	T	T	T	T	T	T	T	T	GBAA_RS08750	-	MBL fold metallo-hydrolase	missense_variant c.207G>T p.Glu69Asp
NC_007530	Chromosome	1632687 G	A	A	A	A	A	A	A	A	A	A	A	A	A	A	GBAA_RS08750	-	MBL fold metallo-hydrolase	missense_variant c.953G>A p.Cys318Tyr
NC_007530	Chromosome	1632989 A	A	A	A	A	A	G	G	G	G	G	G	G	G	G	GBAA_RS08750	-	MBL fold metallo-hydrolase	missense_variant c.1255A>G p.Thr419Ala
NC_007530	Chromosome	1643200 G	A	A	A	A	A	A	A	A	A	A	A	A	A	A	IGR	-	-	-
NC_007530	Chromosome	1647389 T	A	A	A	A	A	A	A	A	A	A	A	A	A	A	IGR	-	-	-
NC_007530	Chromosome	1661500 T	C	C	C	C	C	T	T	T	T	T	T	T	T	T	GBAA_RS08915	-	DUF3942 domain-containing protein	missense_variant c.11T>C p.Val4Ala
NC_007530	Chromosome	1680542 A	G	G	G	G	G	A	A	A	A	A	A	A	A	A	GBAA_RS09020	-	UMP kinase	missense_variant c.587T>C p.Val196Ala
NC_007530	Chromosome	1689676 C	T	T	T	T	T	C	C	C	C	C	C	C	C	C	GBAA_RS09050	-	helicase	stop_gained c.1234C>T p.Gln412*
NC_007530	Chromosome	1696384 A	A	A	A	A	A	G	G	G	G	G	G	G	G	G	GBAA_RS09085	-	aspartate kinase	synonymous_variant c.186T>C p.Ser62Ser
NC_007530	Chromosome	1699244 G	A	A	A	A	A	A	A	A	A	A	A	A	A	A	GBAA_RS09105	-	membrane protein	missense_variant c.580G>A p.Gly194Arg
NC_007530	Chromosome	1712299 C	T	T	T	T	T	C	C	C	C	C	C	C	C	C	IGR	-	-	-
NC_007530	Chromosome	1717874 T	C	C	C	C	C	C	C	C	C	C	C	C	C	C	GBAA_RS09190	-	GNAT family acetyltransferase	missense_variant c.71T>C p.Val24Ala
NC_007530	Chromosome	1717887 A	A	A	A	A	A	G	G	G	G	G	G	G	G	G	GBAA_RS09190	-	GNAT family acetyltransferase	synonymous_variant c.84A>G p.Pro28Pro
NC_007530	Chromosome	1721395 G	G	G	G	G	G	A	A	A	A	A	A	A	A	A	IGR	-	-	-
NC_007530	Chromosome	1721396 G	G	G	G	G	G	T	T	T	T	T	T	T	T	T	IGR	-	-	-
NC_007530	Chromosome	1724259 C	C	C	C	C	C	T	T	T	T	T	T	T	T	T	GBAA_RS09215	-	hypothetical protein	synonymous_variant c.195C>T p.Ser65Ser
NC_007530	Chromosome	1724992 A	G	G	G	G	G	A	A	A	A	A	A	A	A	A	GBAA_RS09220	-	DUF421 domain-containing protein	missense_variant c.484A>G p.Ser162Gly
NC_007530	Chromosome	1728049 G	A	A	A	A	A	A	A	A	A	A	A	A	A	A	GBAA_RS09240	-	hypothetical protein	missense_variant c.217G>A p.Ala73Thr
NC_007530	Chromosome	1739713 C	T	T	T	T	T	C	C	C	C	C	C	C	C	C	IGR	-	-	-
NC_007530	Chromosome	1742426 A	A	A	A	A	A	G	G	G	G	G	G	G	G	G	GBAA_RS09310	-	MFS transporter	synonymous_variant c.921A>G p.Leu307Leu
NC_007530	Chromosome	1756925 C	C	C	C	C	C	T	T	T	T	T	T	T	T	T	GBAA_RS09375	-	class I SAM-dependent methyltransferase	missense_variant c.1606C>T p.Arg536Cys

NC_007530	Chromosome	1760042	A	T	T	T	T	T	A	A	A	A	A	A	A	A	A	GBAA_RS09390	-	DNA-binding response regulator	missense_variant c.466A>T p.Thr156Ser
NC_007530	Chromosome	1768483	T	T	T	T	T	T	A	A	A	A	A	A	A	A	A	IGR	-	-	-
NC_007530	Chromosome	1781256	C	C	C	C	C	C	T	T	T	T	T	T	T	T	T	IGR	-	-	-
NC_007530	Chromosome	1793139	G	A	A	A	A	A	A	A	A	A	A	A	A	A	A	IGR	-	-	-
NC_007530	Chromosome	1797708	T	C	C	C	C	C	C	C	C	C	C	C	C	C	C	GBAA_RS09545	brnQ	branched-chain amino acid transport system II carrier protein	missense_variant c.910T>C p.Tyr304His
NC_007530	Chromosome	1798031	C	T	T	T	T	T	C	C	C	C	C	C	C	C	C	GBAA_RS09545	brnQ	branched-chain amino acid transport system II carrier protein	synonymous_variant c.1233C>T p.Cys411Cys
NC_007530	Chromosome	1798769	G	T	T	T	T	T	T	T	T	T	T	T	T	T	T	IGR	-	-	-
NC_007530	Chromosome	1804099	A	G	G	G	G	G	A	A	A	A	A	A	A	A	A	GBAA_RS09575	-	LysR family transcriptional regulator	synonymous_variant c.633A>G p.Pro211Pro
NC_007530	Chromosome	1805178	A	A	A	A	A	A	G	G	G	G	G	G	G	G	G	GBAA_RS09585	-	DsbA family oxidoreductase	synonymous_variant c.219A>G p.Gly73Gly
NC_007530	Chromosome	1814541	C	T	T	T	T	T	T	T	T	T	T	T	T	T	T	IGR	-	-	-
NC_007530	Chromosome	1816061	T	T	T	T	T	T	G	G	G	G	G	G	G	G	G	GBAA_RS09635	menE	o-succinylbenzoate--CoA ligase	synonymous_variant c.876T>G p.Gly292Gly
NC_007530	Chromosome	1819760	A	A	A	A	A	A	C	C	C	C	C	C	C	C	C	GBAA_RS09655	-	ABC transporter ATP-binding protein	missense_variant c.180A>C p.Glu60Asp
NC_007530	Chromosome	1823153	G	C	C	C	C	C	C	C	C	C	C	C	C	C	C	GBAA_RS09675	-	LysR family transcriptional regulator	missense_variant c.616C>G p.His206Asp
NC_007530	Chromosome	1832719	T	C	C	C	C	C	C	C	C	C	C	C	C	C	C	GBAA_RS09720	cydC	thiol reductant ABC exporter subunit CydC	synonymous_variant c.1071T>C p.Asp357Asp
NC_007530	Chromosome	1833130	G	T	T	T	T	T	T	T	T	T	T	T	T	T	T	GBAA_RS09720	cydC	thiol reductant ABC exporter subunit CydC	missense_variant c.1482G>T p.Met494Ile
NC_007530	Chromosome	1835115	G	A	A	A	A	A	A	A	A	A	A	A	A	A	A	GBAA_RS09735	-	LD-carboxypeptidase	synonymous_variant c.396C>T p.Gly132Gly
NC_007530	Chromosome	1836607	C	T	T	T	T	T	T	T	T	T	T	T	T	T	T	IGR	-	-	-
NC_007530	Chromosome	1841835	T	C	C	C	C	C	C	C	C	C	C	C	C	C	C	GBAA_RS09770	-	ABC transporter ATP-binding protein	synonymous_variant c.498T>C p.Ser166Ser
NC_007530	Chromosome	1855937	C	T	T	T	T	T	T	T	T	T	T	T	T	T	T	GBAA_RS09845	-	lipoprotein	missense_variant c.1394C>T p.Thr465Ile
NC_007530	Chromosome	1864565	T	C	C	C	C	C	T	T	T	T	T	T	T	T	T	GBAA_RS09885	-	N-acetyltransferase	stop_lost&splice_region_variant c.523T>C p.Ter175Glnext*?
NC_007530	Chromosome	1876427	C	T	T	T	T	T	C	C	C	C	C	C	C	C	C	GBAA_RS09950	-	DUF1963 domain-containing protein	missense_variant c.209C>T p.Ala70Val
NC_007530	Chromosome	1878452	T	G	G	G	G	G	G	G	G	G	G	G	G	G	G	GBAA_RS09960	-	DUF4083 domain-containing protein	missense_variant c.72T>G p.Phe24Leu
NC_007530	Chromosome	1894109	C	C	C	C	C	C	T	C	C	C	C	C	C	C	C	GBAA_RS10050	-	DNA starvation/stationary phase protection protein	missense_variant c.205C>T p.Pro69Ser
NC_007530	Chromosome	1894860	C	T	T	T	T	T	C	C	C	C	C	C	C	C	C	GBAA_RS10055	-	DUF3939 domain-containing protein	missense_variant c.406C>T p.His136Tyr
NC_007530	Chromosome	1903537	C	C	C	C	C	C	C	C	C	C	C	C	C	A	C	IGR	-	-	-
NC_007530	Chromosome	1912074	C	C	C	C	C	C	T	T	T	T	T	T	T	T	T	GBAA_RS10165	-	oxidoreductase	missense_variant c.539C>T p.Ala180Val
NC_007530	Chromosome	1912852	A	C	C	C	C	C	C	C	C	C	C	C	C	C	C	GBAA_RS10170	-	NADPH dehydrogenase	missense_variant c.411T>G p.His137Gln
NC_007530	Chromosome	1914878	C	T	T	T	T	T	T	T	T	T	T	T	T	T	T	IGR	-	-	-
NC_007530	Chromosome	1926029	T	T	T	T	T	T	G	G	G	G	G	G	G	G	G	GBAA_RS10240	-	TIGR03943 family protein	synonymous_variant c.765T>G p.Thr255Thr
NC_007530	Chromosome	1938186	C	C	C	C	C	C	T	T	T	T	T	T	T	T	T	GBAA_RS10315	-	stage II sporulation protein P	missense_variant c.1112C>T p.Ala371Val
NC_007530	Chromosome	1938332	T	T	T	T	T	T	C	C	C	C	C	C	C	C	C	IGR	-	-	-
NC_007530	Chromosome	1939286	C	A	A	A	A	A	C	C	C	C	C	C	C	C	C	GBAA_RS10325	-	D-alanyl-D-alanine carboxypeptidase	missense_variant c.325C>A p.Leu109Ile
NC_007530	Chromosome	1948662	G	A	A	A	A	A	A	A	A	A	A	A	A	A	A	GBAA_RS10375	-	class I SAM-dependent methyltransferase	missense_variant c.140C>T p.Ser47Leu
NC_007530	Chromosome	1949099	A	G	G	G	G	G	G	G	G	G	G	G	G	G	G	GBAA_RS10380	-	N-acetyltransferase	missense_variant c.583T>C p.Tyr195His
NC_007530	Chromosome	1950257	T	C	C	C	C	C	C	C	C	C	C	C	C	C	C	GBAA_RS10385	-	nitrate reductase	synonymous_variant c.327A>G p.Thr109Thr
NC_007530	Chromosome	1953540	A	C	C	C	C	C	A	A	A	A	A	A	A	A	A	GBAA_RS10400	-	PLP-dependent aminotransferase family protein	stop_gained c.1100T>G p.Leu367*
NC_007530	Chromosome	1954568	G	T	T	T	T	T	T	T	T	T	T	T	T	T	T	GBAA_RS10400	-	PLP-dependent aminotransferase family protein	missense_variant c.72C>A p.Asn24Lys
NC_007530	Chromosome	1972618	A	G	G	G	G	G	G	G	G	G	G	G	G	G	G	GBAA_RS10545	-	DUF3600 domain-containing protein	missense_variant c.271A>G p.Met91Val
NC_007530	Chromosome	1977071	T	T	T	T	T	T	C	C	C	C	C	C	C	C	C	GBAA_RS10570	-	protease	missense_variant c.833A>G p.Lys278Arg
NC_007530	Chromosome	1982730	G	C	C	C	C	C	C	C	C	C	C	C	C	C	C	GBAA_RS10595	-	nitrate reductase subunit alpha	missense_variant c.925G>C p.Val309Leu
NC_007530	Chromosome	1985008	G	G	G	G	G	G	A	A	A	A	A	A	A	A	A	GBAA_RS10595	-	nitrate reductase subunit alpha	missense_variant c.3203G>A p.Arg1068Gln
NC_007530	Chromosome	1991620	G	A	A	A	A	A	G	G	G	G	G	G	G	G	G	IGR	-	-	-
NC_007530	Chromosome	1995029	C	T	T	T	T	T	C	C	C	C	C	C	C	C	C	GBAA_RS10645	-	molybdopterin molybdenumtransferase MoeA	missense_variant c.1160C>T p.Ala387Val
NC_007530	Chromosome	2014888	C	T	T	T	T	T	T	T	T	T	T	T	T	T	T	GBAA_RS10760	-	hypothetical protein	missense_variant c.221G>A p.Ser74Asn
NC_007530	Chromosome	2026733	T	C	C	C	C	C	C	C	C	C	C	C	C	C	C	GBAA_RS10830	-	DUF2085 domain-containing protein	missense_variant c.242T>C p.Ile81Thr
NC_007530	Chromosome	2029380	G	A	A	A	A	A	A	A	A	A	A	A	A	A	A	GBAA_RS29995	-	hypothetical protein	synonymous_variant c.927G>A p.Pro309Pro
NC_007530	Chromosome	2032776	G	A	A	A	A	A	A	A	A	A	A	A	A	A	A	IGR	-	-	-
NC_007530	Chromosome	2033484	C	G	G	G	G	G	C	C	C	C	C	C	C	C	C	GBAA_RS10850	-	isoleucine--tRNA ligase	missense_variant c.436C>G p.Pro146Ala
NC_007530	Chromosome	2042095	G	T	T	T	T	T	T	T	T	T	T	T	T	T	T	GBAA_RS10880	-	hypothetical protein	missense_variant c.210G>T p.Met70Ile
NC_007530	Chromosome	2045712	A	C	C	C	C	C	C	C	C	C	C	C	C	C	C	GBAA_RS10890	essC	type VII secretion protein EssC	synonymous_variant c.2652A>C p.Gly884Gly
NC_007530	Chromosome	2059040	A	G	G	G	G	G	G	G	G	G	G	G	G	G	G	GBAA_RS10955	-	SMI1/KNR4 family protein	missense_variant c.493A>G p.Ile165Val
NC_007530	Chromosome	2064548	C	T	T	T	T	T	C	C	C	C	C	C	C	C	C	GBAA_RS10990	-	sodium:solute symporter	synonymous_variant c.616C>T p.Leu206Leu
NC_007530	Chromosome	2068677	C	C	C	C	C	C	T	T	T	T	T	T	T	T	T	GBAA_RS11010	-	hypothetical protein	missense_variant c.53C>T p.Ala18Val
NC_007530	Chromosome	2071032	C	T	T	T	T	T	C	C	C	C	C	C	C	C	C	GBAA_RS11020	-	alpha/beta hydrolase	missense_variant c.149C>T p.Ala50Val
NC_007530	Chromosome	2097700	T	T	T	T	T	T	A	A	A	A	A	A	A	A	A	GBAA_RS11165	-	membrane protein	missense_variant c.258A>T p.Lys86Asn
NC_007530	Chromosome	2099715	G	G	G	G	G	G	A	A	A	A	A	A	A	A	A	GBAA_RS11180	asnB	asparagine synthetase B	missense_variant c.115G>A p.Gly39Arg
NC_007530	Chromosome	2101633	A	G	G	G	G	G	G	G	G	G	G	G	G	G	G	GBAA_RS11185	-	GNAT family N-acetyltransferase	missense_variant c.38A>G p.Asn13Ser
NC_007530	Chromosome	2103788	C	C	C	C	C	C	T	T	T	T	T	T	T	T	T	IGR	-	-	-
NC_007530	Chromosome	2109160	T	G	G	G	G	G	G	G	G	G	G	G	G	G	G	GBAA_RS11240	-	mechanosensitive ion channel family protein	missense_variant c.504T>G p.Asn168Lys
NC_007530	Chromosome	2113297	T	T	T	T	T	T	C	C	C	C	C	C	C	C	C	GBAA_RS11265	-	alcohol dehydrogenase AdhP	synonymous_variant c.1011T>C p.Phe337Phe
NC_007530	Chromosome	2113910	C	T	T	T	T	T	T	T	T	T	T	T	T	T	T	GBAA_RS11270	-	peptide ABC transporter permease	synonymous_variant c.615G>A p.Leu205Leu
NC_007530	Chromosome	2115451	G	A	A	A	A	A	A	A	A	A	A	A	A	A	A	IGR	-	-	-
NC_007530	Chromosome	2116163	C	T	T	T	T	T	C	C	C	C	C	C	C	C	C	GBAA_RS11280	-	D-alanyl-D-alanine carboxypeptidase	missense_variant c.122C>T p.Ser41Leu
NC_007530	Chromosome	2122348	T	A	A	A	A	A	A	A	A	A	A	A	A	A	A	GBAA_RS11315	-	NAD(P)H-dependent oxidoreductase	missense_variant c.6T>A p.Asn2Lys
NC_007530	Chromosome	2125406	T	G	G	G	G	G	T	T	T	T	T	T	T	T	T	GBAA_RS11330	-	glycine/betaine ABC transporter ATP-binding protein	synonymous_variant c.573T>G p.Val191Val
NC_007530	Chromosome	2127289	T	T	T	T	T	T	T	C	T	T	T	T	T	T	T	GBAA_RS11335	-	ABC transporter permease	missense_variant c.1505T>C p.Leu502Pro
NC_007530	Chromosome	2156613	G	T	T	T	T	T	G	G	G	G	G	G	G	G	G	GBAA_RS11495	-	WXG100 family type VII secretion target	missense_variant c.893C>A p.Thr298Lys
NC_007530	Chromosome	2167531	A	A	A	A	A	A	A	A	G	G	G	G	G	G	G	GBAA_RS11565	-	hypothetical protein	missense_variant c.76A>G p.Thr26Ala
NC_007530	Chromosome	2178917	G	A	A	A	A	A	A	A	A	A	A	A	A	A	A	GBAA_RS11640	-	penicillin-binding protein 1A	missense_variant c.1363G>A p.Ala455Thr
NC_007530	Chromosome	2185024	G	A	A	A	A	A	A	A	A	A	A	A	A	A	A	GBAA_RS11660	prpD	2-methylcitrate dehydratase	missense_variant c.1322G>A p.Ser441Asn
NC_007530	Chromosome	2189971	C	C	C	C	C	C	T	T	T	T	T	T	T	T	T	GBAA_RS11680	mmsA	methylmalonate-semialdehyde dehydrogenase (CoA acylating)	synonymous_variant c.1098C>T p.Tyr366Tyr
NC_007530	Chromosome	2193023	G	T	T	T	T	T	T	T	T	T	T	T	T	T	T	IGR	-	-	-
NC_007530	Chromosome	2197835	C	T	T	T	T	T	T	T	T	T	T	T	T	T	T	IGR	-	-	-
NC_007530	Chromosome	2200153	G	A	A	A	A	A	A	A	A	A	A	A	A	A	A	GBAA_RS11735	-	hypothetical protein	missense_variant c.37G>A p.Val13Ile
NC_007530	Chromosome	2200548	T	T	T	T	T	T	A	T	T	T	T	T	T	T	T	GBAA_RS11740	-	MFS transporter	missense_variant c.283T>A p.Ser95Thr
NC_007530	Chromosome	2204132	G	G	G	G	G	G	A	A	A	A	A	A	A	A	A	GBAA_RS11755	-	(2,3-dihydroxybenzoyl)adenylate synthase	stop_gained c.66G>A p.Trp22*
NC_007530	Chromosome	2216166	A	G	G	G	G	G	A	A	A	A	A	A	A	A	A	GBAA_RS11780	-	4'-phosphopantetheinyl transferase	missense_variant c.679A>G p.Ile227Val
NC_007530	Chromosome	2219730	A	T	T	T	T	T	A	A	A	A	A	A	A	A	A	GBAA_RS11805	-	peptidase S8	synonymous_variant c.189T>A p.Thr63Thr
NC_007530	Chromosome	2241703	T	T	T	T	T	T	C	C	C	C	C	C	C	C	C	GBAA_RS11915	-	hypothetical protein	missense_variant c.185A>G p.Tyr62Cys
NC_007530	Chromosome	2244381	G	G	G	G	G	G	A	A	A	A	A	A	A	A	A	IGR	-	-	-
NC_007530	Chromosome	2265748	T	T	T	T	T	T	C	C	C	C	C	C	C	C	C	GBAA_RS12075	-	LysR family transcriptional regulator	synonymous_variant c.747A>G p.Glu249Glu
NC_007530	Chromosome	2267616	G	G	G	G	G	G	G	A	G	G	G	G	G	G	G	GBAA_RS12080	-	aspartate-semialdehyde dehydrogenase	synonymous_variant c.993G>A p.Ala331Ala
NC_007530	Chromosome	2271933	C	T	T	T	T	T	T	T	T	T	T	T	T	T	T	GBAA_RS12105	-	GNAT family N-acetyltransferase	missense_variant c.565C>T p.Leu189Phe
NC_007530	Chromosome	2290789	T	T	T	T	T	T	T	T	C	T	T	T	T	T	C	IGR	-	-	-
NC_007530	Chromosome	2295848	G	A	A	A	A	A	A	A	A	A	A	A	A	A	A	GBAA_RS12230	-	acetyltransferase	synonymous_variant c.207G>A p.Arg69Arg
NC_007530	Chromosome	2299647	A	G	G	G	G	G	G	G	G	G	G	G	G	G	G	IGR	-	-	-
NC_007530	Chromosome	2311534	G	G	G	G	G	G	A	A	A	A	A	A	A	A	A	GBAA_RS12305	-	VOC family protein	missense_variant c.52G>A p.Ala18Thr
NC_007530	Chromosome	2313643	A	G	G	G	G	G	G	G	G	G	G	G	G	G	G	GBAA_RS12315	-	indolepyruvate decarboxylase	missense_variant c.1181T>C p.Ile394Thr
NC_007530	Chromosome	2318414	C	T	T	T	T	T	T	T	T	T	T	T	T	T	T	GBAA_RS12350	-	hypothetical protein	missense_variant c.191G>A p.Ser64Asn
NC_007530	Chromosome	2341327	C	T	T	T	T	T	T	T	T	T	T	T	T	T	T	GBAA_RS12445	-	5-deoxy-glucuronate isomerase	synonymous_variant c.273C>T p.Thr91Thr
NC_007530	Chromosome	2363020	A	C	C	C	C	C	C	C	C	C	C	C	C	C	C	GBAA_RS12545	-	NAD(P)/FAD-dependent oxidoreductase	missense_variant c.479A>C p.Gln160Pro
NC_007530	Chromosome	2371243	T	C	C	C	C	C	C	C	C	C	C	C	C	C	C	GBAA_RS12590	-	acyl-CoA dehydrogenase	missense_variant c.746T>C p.Val249Ala
NC_007530	Chromosome	2373661	G	A	A	A	A	A	A	A	A	A	A	A	A	A	A	GBAA_RS12605	-	hydroxymethylglutaryl-CoA lyase	synonymous_variant c.402G>A p.Glu134Glu
NC_007530	Chromosome	2390924	G	A	A	A	A	A	G	G	G	G	G	G	G	G	G	GBAA_RS12695	-	peptidase M4	missense_variant c.2009G>A p.Ser670Asn
NC_007530	Chromosome	2417068	G	A	A	A	A	A	G	G	G	G	G	G	G	G	G	GBAA_RS12835	-	acetamidase	missense_variant c.19G>A p.Glu7Lys
NC_007530	Chromosome	2422827	A	A	A	A	A	A	G	G	G	G	G	G	G	G	G	GBAA_RS30180	-	ABC transporter ATP-binding protein	synonymous_variant c.834A>G p.Ala278Ala
NC_007530	Chromosome	2423657	A	G	G	G	G	G	G	G	G	G	G	G	G	G	G	IGR	-	-	-
NC_007530	Chromosome	2424305	T	C	C	C	C	C	C	C	C	C	C	C	C	C	C	GBAA_RS12865	-	hypothetical protein	missense_variant c.443T>C p.Val148Ala
NC_007530	Chromosome	2431111	T	T	T	T	T	T	T	A	T	T	T	T	T	T	T	GBAA_RS12895	-	homoserine dehydrogenase	synonymous_variant c.1002T>A p.Ile334Ile
NC_007530	Chromosome	2433911	A	A	A	A	A	A	G	G	G	G	G	G	G	G	G	IGR	-	-	-
NC_007530	Chromosome	2435752	T	C	C	C	C	C	C	C	C	C	C	C	C	C	C	GBAA_RS30190	-	hypothetical protein	initiator_codon_variant c.3A>G p.Ile1?
NC_007530	Chromosome	2443215	T	C	C	C	C	C	C	C	C	C	C	C	C	C	C	IGR	-	-	-
NC_007530	Chromosome	2452536	T	C	C	C	C	C	C	C	C	C	C	C	C	C	C	IGR	-	-	-
NC_007530	Chromosome	2476676	C	T	T	T	T	T	T	T	T	T	T	T	T	T	T	GBAA_RS13125	-	YafY family transcriptional regulator	missense_variant c.74C>T p.Thr25Ile
NC_007530	Chromosome	2485974	G	G	G	G	G	G	A	A	A	A	A	A	A	A	A	IGR	-	-	-
NC_007530	Chromosome	2489368	G	A	A	A	A	A	A	A	A	A	A	A	A	A	A	GBAA_RS13185	-	DUF4269 domain-containing protein	missense_variant c.472G>A p.Val158Ile
NC_007530	Chromosome	2495392	G	A	A	A	A	A	A	A	A	A	A	A	A	A	A	GBAA_RS13220	-	penicillin-binding protein	synonymous_variant c.72G>A p.Ala24Ala
NC_007530	Chromosome	2497724	T	G	G	G	G	G	G	G	G	G	G	G	G	G	G	GBAA_RS13230	-	MFS transporter	missense_variant c.928T>G p.Phe310Val
NC_007530	Chromosome	2519082	T	A	A	A	A	A	A	A	A	A	A	A	A	A	A	GBAA_RS13370	-	hypothetical protein	missense_variant c.221A>T p.Asn74Ile
NC_007530	Chromosome	2528887	T	A	A	A	A	A	A	A	A	A	A	A	A	A	A	IGR	-	-	-
NC_007530	Chromosome	2530689	T	T	T	T	T	T	C	C	C	C	C	C	C	C	C	GBAA_RS13440	-	lipase	missense_variant c.481T>C p.Trp161Arg
NC_007530	Chromosome	2532434	C	C	C	C	C	C	T	T	T	T	T	T	T	T	T	GBAA_RS13445	-	2,4-diaminobutyrate decarboxylase	synonymous_variant c.309G>A p.Gly103Gly
NC_007530	Chromosome	2538740	A	G	G	G	G	G	G	G	G	G	G	G	G	G	G	GBAA_RS13450	-	non-ribosomal peptide synthetase	missense_variant c.689T>C p.Val230Ala
NC_007530	Chromosome	2546224	C	T	T	T	T	T	T	T	T	T	T	T	T	T	T	IGR	-	-	-
NC_007530	Chromosome	2547446	C	C	C	C	C	C	T	T	T	T	T	T	T	T	T	GBAA_RS13470	-	membrane protein	missense_variant c.19G>A p.Gly7Arg
NC_007530	Chromosome	2550959	T	C	C	C	C	C	T	T	T	T	T	T	T	T	T	GBAA_RS13490	-	hypothetical protein	synonymous_variant c.564A>G p.Gln188Gln
NC_007530	Chromosome	2559878	C	T	T	T	T	T	C	C	C	C	C	C	C	C	C	GBAA_RS13540	-	lipoprotein	synonymous_variant c.339G>A p.Val113Val
NC_007530	Chromosome	2561497	T	C	C	C	C	C	C	C	C	C	C	C	C	C	C	GBAA_RS13550	ypeB	germination protein YpeB	missense_variant c.796A>G p.Lys266Glu
NC_007530	Chromosome	2564504	T	T	T	T	T	T	C	C	C	C	C	C	C	C	C	GBAA_RS13565	-	6-aminohexanoate-dimer hydrolase	missense_variant c.74A>G p.Glu25Gly
NC_007530	Chromosome	2564877	G	A	A	A	A	A	A	A	A	A	A	A	A	A	A	GBAA_RS13570	kynA	tryptophan 2,3-dioxygenase	synonymous_variant c.657C>T p.Leu219Leu
NC_007530	Chromosome	2569602	T	T	T	T	T	T	C	C	C	C	C	C	C	C	C	GBAA_RS13595	-	hypothetical protein	synonymous_variant c.777T>C p.Ser259Ser
NC_007530	Chromosome	2580218	G	A	A	A	A	A	A	A	A	A	A	A	A	A	A	GBAA_RS13670	lpdA	dihydrolipoyl dehydrogenase	synonymous_variant c.231C>T p.Ile77Ile
NC_007530	Chromosome	2601593	T	T	T	T	T	T	C	C	C	C	C	C	C	C	C	GBAA_RS13820	-	S-layer protein	synonymous_variant c.555T>C p.Arg185Arg
NC_007530	Chromosome	2610245	C	C	C	C	C	C	T	T	T	T	T	T	T	T	T	IGR	-	-	-
NC_007530	Chromosome	2611560	C	T	T	T	T	T	T	T	T	T	T	T	T	T	T	GBAA_RS13880	-	class I SAM-dependent methyltransferase	synonymous_variant c.411G>A p.Ala137Ala
NC_007530	Chromosome	2614351	A	G	G	G	G	G	G	G	G	G	G	G	G	G	G	GBAA_RS13890	recQ	DNA helicase RecQ	missense_variant c.331T>C p.Phe111Leu
NC_007530	Chromosome	2615217	A	A	A	A	A	A	G	G	G	G	G	G	G	G	G	IGR	-	-	-
NC_007530	Chromosome	2634495	C	C	C	C	C	C	T	T	T	T	T	T	T	T	T	GBAA_RS14000	-	sarcosine oxidase subunit alpha	synonymous_variant c.213C>T p.Arg71Arg
NC_007530	Chromosome	2634964	A	G	G	G	G	G	G	G	G	G	G	G	G	G	G	GBAA_RS14005	-	sarcosine oxidase subunit alpha	missense_variant c.353A>G p.Asp118Gly
NC_007530	Chromosome	2635073	A	A	A	A	A	A	G	G	G	G	G	G	G	G	G	GBAA_RS14005	-	sarcosine oxidase subunit alpha	synonymous_variant c.462A>G p.Ser154Ser
NC_007530	Chromosome	2637807	G	G	G	G	G	G	A	A	A	A	A	A	A	A	A	IGR	-	-	-
NC_007530	Chromosome	2643851	C	C	C	C	C	C	A	C	C	C	C	C	C	C	C	GBAA_RS14055	-	peptidase P60	missense_variant c.296G>T p.Gly99Val
NC_007530	Chromosome	2656190	T	C	C	C	C	C	C	C	C	C	C	C	C	C	C	GBAA_RS14130	-	haloacid dehalogenase	missense_variant c.1352A>G p.Glu451Gly
NC_007530	Chromosome	2664066	A	A	A	A	A	A	C	C	C	C	C	C	C	C	C	GBAA_RS14165	-	hypothetical protein	missense_variant c.125A>C p.Asp42Ala
NC_007530	Chromosome	2664662	G	A	A	A	A	A	A	A	A	A	A	A	A	A	A	GBAA_RS14170	-	MFS transporter	synonymous_variant c.1191C>T p.Phe397Phe
NC_007530	Chromosome	2664845	G	A	A	A	A	A	A	A	A	A	A	A	A	A	A	GBAA_RS14170	-	MFS transporter	synonymous_variant c.1008C>T p.Val336Val
NC_007530	Chromosome	2668610	T	A	A	A	A	A	T	T	T	T	T	T	T	T	T	GBAA_RS14190	-	SgrR family transcriptional regulator	missense_variant c.1238A>T p.Glu413Val
NC_007530	Chromosome	2668966	C	T	T	T	T	T	T	T	T	T	T	T	T	T	T	GBAA_RS14190	-	SgrR family transcriptional regulator	synonymous_variant c.882G>A p.Ala294Ala
NC_007530	Chromosome	2669955	C	C	C	C	C	C	T	T	T	T	T	T	T	T	T	GBAA_RS14195	-	MFS transporter	missense_variant c.7C>T p.His3Tyr
NC_007530	Chromosome	2679609	G	G	G	G	G	G	A	A	A	A	A	A	A	A	A	GBAA_RS30295	-	hypothetical protein	missense_variant c.53G>A p.Gly18Glu
NC_007530	Chromosome	2695371	C	T	T	T	T	T	T	T	T	T	T	T	T	T	T	GBAA_RS14385	-	hypothetical protein	synonymous_variant c.336G>A p.Glu112Glu
NC_007530	Chromosome	2698633	T	C	C	C	C	C	C	C	C	C	C	C	C	C	C	GBAA_RS14405	-	hypothetical protein	missense_variant c.20A>G p.Gln7Arg
NC_007530	Chromosome	2706197	C	A	A	A	A	A	A	A	A	A	A	A	A	A	A	GBAA_RS14460	-	N-acetyltransferase	missense_variant c.310G>T p.Asp104Tyr
NC_007530	Chromosome	2706276	C	A	A	A	A	A	A	A	A	A	A	A	A	A	A	GBAA_RS14460	-	N-acetyltransferase	missense_variant c.231G>T p.Glu77Asp
NC_007530	Chromosome	2711219	A	G	G	G	G	G	G	G	G	G	G	G	G	G	G	IGR	-	-	-
NC_007530	Chromosome	2731760	G	G	G	G	G	G	A	A	A	A	A	A	A	A	A	GBAA_RS14560	-	DUF4020 domain-containing protein	missense_variant c.106C>T p.Leu36Phe
NC_007530	Chromosome	2740303	G	A	A	A	A	A	G	G	G	G	G	G	G	G	G	GBAA_RS14615	-	DUF2332 domain-containing protein	missense_variant c.821C>T p.Thr274Ile
NC_007530	Chromosome	2749671	A	G	G	G	G	G	G	G	G	G	G	G	G	G	G	GBAA_RS14650	-	autoinducer-2 kinase	missense_variant c.1042A>G p.Ser348Gly
NC_007530	Chromosome	2754227	T	C	C	C	C	C	T	T	T	T	T	T	T	T	T	GBAA_RS14685	-	MFS transporter	missense_variant c.1208A>G p.Glu403Gly
NC_007530	Chromosome	2754925	A	C	C	C	C	C	C	C	C	C	C	C	C	C	C	GBAA_RS14685	-	MFS transporter	synonymous_variant c.510T>G p.Leu170Leu
NC_007530	Chromosome	2779596	G	A	A	A	A	A	A	A	A	A	A	A	A	A	A	IGR	-	-	-
NC_007530	Chromosome	2794498	T	C	C	C	C	C	T	T	T	T	T	T	T	T	T	IGR	-	-	-
NC_007530	Chromosome	2803221	A	G	G	G	G	G	G	G	G	G	G	G	G	G	G	GBAA_RS14915	-	MoxR family ATPase	missense_variant c.544T>C p.Trp182Arg
NC_007530	Chromosome	2804918	C	C	C	C	C	C	T	T	T	T	T	T	T	T	T	GBAA_RS14930	-	MarR family transcriptional regulator	missense_variant c.340G>A p.Ala114Thr
NC_007530	Chromosome	2809650	T	T	T	T	T	T	C	C	C	C	C	C	C	C	C	GBAA_RS14955	-	ABC-2 transporter permease	missense_variant c.301A>G p.Met101Val
NC_007530	Chromosome	2825693	G	A	A	A	A	A	G	G	G	G	G	G	G	G	G	GBAA_RS15045	-	sensor histidine kinase	synonymous_variant c.39C>T p.Ser13Ser
NC_007530	Chromosome	2833618	G	A	A	A	A	A	A	A	A	A	A	A	A	A	A	IGR	-	-	-
NC_007530	Chromosome	2849931	C	T	T	T	T	T	T	T	T	T	T	T	T	T	T	GBAA_RS15190	-	allophanate hydrolase	missense_variant c.698G>A p.Gly233Glu
NC_007530	Chromosome	2861361	A	A	A	A	A	A	G	G	G	G	G	G	G	G	G	GBAA_RS15250	-	histidine transporter	missense_variant c.203T>C p.Leu68Pro
NC_007530	Chromosome	2867041	G	A	A	A	A	A	G	G	G	G	G	G	G	G	G	GBAA_RS15280	-	pyruvate, water dikinase	stop_gained c.1423C>T p.Gln475*
NC_007530	Chromosome	2868649	T	G	G	G	G	G	G	G	G	G	G	G	G	G	G	GBAA_RS30375	-	hypothetical protein	missense_variant c.151A>C p.Thr51Pro
NC_007530	Chromosome	2895903	G	G	G	G	G	G	G	G	A	A	A	A	A	A	A	GBAA_RS15425	-	spore gernimation protein	missense_variant c.14C>T p.Ser5Phe
NC_007530	Chromosome	2900498	C	C	C	C	T	C	C	C	C	C	C	C	C	C	C	IGR	-	-	-
NC_007530	Chromosome	2901180	T	C	C	C	C	C	C	C	C	C	C	C	C	C	C	GBAA_RS15445	glsA	glutaminase 2	missense_variant c.492A>G p.Ile164Met
NC_007530	Chromosome	2922836	T	T	T	T	T	T	C	C	C	C	C	C	C	C	C	GBAA_RS15535	-	hypothetical protein	missense_variant c.25A>G p.Thr9Ala
NC_007530	Chromosome	2930049	G	G	G	G	G	G	A	A	A	A	A	A	A	A	A	GBAA_RS15570	-	hypothetical protein	missense_variant c.323C>T p.Ser108Phe
NC_007530	Chromosome	2932222	C	C	C	C	C	C	T	T	T	T	T	T	T	T	T	IGR	-	-	-
NC_007530	Chromosome	2941844	A	G	G	G	G	G	G	G	G	G	G	G	G	G	G	GBAA_RS15625	-	lipoprotein	missense_variant c.391A>G p.Ser131Gly
NC_007530	Chromosome	2941866	C	T	T	T	T	T	T	T	T	T	T	T	T	T	T	GBAA_RS15625	-	lipoprotein	missense_variant c.413C>T p.Ala138Val
NC_007530	Chromosome	2942510	T	G	G	G	G	G	G	G	G	G	G	G	G	G	G	GBAA_RS15630	-	GNAT family N-acetyltransferase	missense_variant c.363A>C p.Arg121Ser
NC_007530	Chromosome	2948557	G	A	A	A	A	A	G	G	G	G	G	G	G	G	G	GBAA_RS15665	-	molecular chaperone HscC	missense_variant c.878C>T p.Pro293Leu
NC_007530	Chromosome	2950393	G	G	G	G	G	G	T	T	T	T	T	T	T	T	T	IGR	-	-	-
NC_007530	Chromosome	2955691	G	A	A	A	A	A	A	A	A	A	A	A	A	A	A	GBAA_RS15690	-	disulfide oxidoreductase	synonymous_variant c.21C>T p.Thr7Thr
NC_007530	Chromosome	2961771	G	A	A	A	A	A	A	A	A	A	A	A	A	A	A	GBAA_RS15730	-	penicillin-binding protein	synonymous_variant c.573C>T p.Gly191Gly
NC_007530	Chromosome	2971794	G	G	G	G	G	G	A	A	A	A	A	A	A	A	A	GBAA_RS15770	-	hypothetical protein	missense_variant c.437G>A p.Gly146Glu
NC_007530	Chromosome	2981913	A	G	G	G	G	G	A	A	A	A	A	A	A	A	A	GBAA_RS15835	-	metallophosphoesterase	synonymous_variant c.996T>C p.Ser332Ser
NC_007530	Chromosome	2982212	T	T	T	T	T	T	C	C	C	C	C	C	C	C	C	GBAA_RS15835	-	metallophosphoesterase	missense_variant c.697A>G p.Ile233Val
NC_007530	Chromosome	2994787	G	A	A	A	A	A	A	A	A	A	A	A	A	A	A	GBAA_RS15895	-	cell wall anchor	missense_variant c.146G>A p.Gly49Glu

NC_007530	Chromosome	2998142	A	A	A	A	A	A	G	G	G	G	G	G	G	G	G	GBAA_RS15910	-	transcriptional regulator	missense_variant c.398A>G p.Asp133Gly
NC_007530	Chromosome	3005779	T	T	T	T	T	T	C	C	C	C	C	C	C	C	C	IGR	-	-	-
NC_007530	Chromosome	3006617	G	A	A	A	A	A	A	A	A	A	A	A	A	A	A	GBAA_RS15960	-	MFS transporter	missense_variant c.662C>T p.Ala221Val
NC_007530	Chromosome	3006769	A	A	A	A	A	A	G	G	G	G	G	G	G	G	G	GBAA_RS15960	-	MFS transporter	synonymous_variant c.510T>C p.Ser170Ser
NC_007530	Chromosome	3017461	C	T	T	T	T	T	T	T	T	T	T	T	T	T	T	GBAA_RS16020	-	gfo/Idh/MocA family oxidoreductase	missense_variant c.407G>A p.Gly136Glu
NC_007530	Chromosome	3024598	C	A	A	A	A	A	C	C	C	C	C	C	C	C	C	GBAA_RS16050	-	hypothetical protein	missense_variant c.250G>T p.Ala84Ser
NC_007530	Chromosome	3025707	C	T	T	T	T	T	T	T	T	T	T	T	T	T	T	IGR	-	-	-
NC_007530	Chromosome	3029321	T	T	T	T	T	T	T	T	C	C	C	C	C	C	C	GBAA_RS16075	-	methyl-accepting chemotaxis protein	missense_variant c.482A>G p.Gln161Arg
NC_007530	Chromosome	3029956	C	T	T	T	T	T	T	T	T	T	T	T	T	T	T	IGR	-	-	-
NC_007530	Chromosome	3033346	G	A	A	A	A	A	A	A	A	A	A	A	A	A	A	GBAA_RS16090	-	MATE family efflux transporter	missense_variant c.1003C>T p.Arg335Cys
NC_007530	Chromosome	3034654	A	G	G	G	G	G	A	A	A	A	A	A	A	A	A	IGR	-	-	-
NC_007530	Chromosome	3050364	T	T	T	T	T	T	T	T	C	C	C	C	C	C	C	GBAA_RS16150	hypF	carbamoyltransferase HypF	synonymous_variant c.951A>G p.Thr317Thr
NC_007530	Chromosome	3050423	A	A	A	A	A	A	A	G	A	A	A	A	A	A	A	GBAA_RS16150	hypF	carbamoyltransferase HypF	missense_variant c.892T>C p.Tyr298His
NC_007530	Chromosome	3059588	G	A	A	A	A	A	A	A	A	A	A	A	A	A	A	GBAA_RS16190	-	3-phosphoserine/phosphohydroxythreonine transaminase	missense_variant c.850C>T p.Leu284Phe
NC_007530	Chromosome	3082555	G	G	G	G	G	G	A	A	A	A	A	A	A	A	A	GBAA_RS16315	-	membrane protein	missense_variant c.215C>T p.Thr72Ile
NC_007530	Chromosome	3086718	A	C	C	C	C	C	C	C	C	C	C	C	C	C	C	GBAA_RS16350	-	thiol-activated cytolysin	missense_variant c.1247T>G p.Val416Gly
NC_007530	Chromosome	3107432	A	G	G	G	G	G	G	G	G	G	G	G	G	G	G	GBAA_RS16455	-	Cys-tRNA(Pro) deacylase	synonymous_variant c.99T>C p.Gly33Gly
NC_007530	Chromosome	3111955	G	A	A	A	A	A	A	A	A	A	A	A	A	A	A	GBAA_RS16485	-	DUF1292 domain-containing protein	synonymous_variant c.66G>A p.Val22Val
NC_007530	Chromosome	3126496	C	T	T	T	T	T	T	T	T	T	T	T	T	T	T	GBAA_RS16575	-	ROK family protein	missense_variant c.199G>A p.Ala67Thr
NC_007530	Chromosome	3126940	G	G	G	G	G	G	G	G	A	A	A	A	A	A	A	GBAA_RS16580	-	MFS transporter	synonymous_variant c.1047C>T p.Ile349Ile
NC_007530	Chromosome	3148331	T	C	C	C	C	C	C	C	C	C	C	C	C	C	C	GBAA_RS16700	-	hypothetical protein	missense_variant c.673A>G p.Met225Val
NC_007530	Chromosome	3154736	G	G	G	G	G	G	G	G	T	T	T	T	T	T	T	GBAA_RS16725	tkt	transketolase	missense_variant c.1444C>A p.His482Asn
NC_007530	Chromosome	3155665	T	G	G	G	G	G	G	G	G	G	G	G	G	G	G	GBAA_RS16725	tkt	transketolase	missense_variant c.515A>C p.Glu172Ala
NC_007530	Chromosome	3155979	C	T	T	T	T	T	T	T	T	T	T	T	T	T	T	GBAA_RS16725	tkt	transketolase	synonymous_variant c.201G>A p.Gly67Gly
NC_007530	Chromosome	3162871	C	T	T	T	T	T	T	T	T	T	T	T	T	T	T	GBAA_RS16765	-	NAD(P)-dependent oxidoreductase	missense_variant c.223G>A p.Asp75Asn
NC_007530	Chromosome	3163858	A	G	G	G	G	G	G	G	G	G	G	G	G	G	G	GBAA_RS16770	-	peptidase M4 family protein	synonymous_variant c.1521T>C p.Gly507Gly
NC_007530	Chromosome	3164962	A	G	G	G	G	G	A	A	A	A	A	A	A	A	A	GBAA_RS16770	-	peptidase M4 family protein	synonymous_variant c.417T>C p.Asp139Asp
NC_007530	Chromosome	3168196	C	A	A	A	A	A	A	A	A	A	A	A	A	A	A	GBAA_RS16780	-	nitroreductase	missense_variant c.509G>T p.Arg170Leu
NC_007530	Chromosome	3177729	C	C	C	C	C	C	A	A	A	A	A	A	A	A	A	IGR	-	-	-
NC_007530	Chromosome	3180799	G	A	A	A	A	A	A	A	A	A	A	A	A	A	A	GBAA_RS16855	-	GntR family transcriptional regulator	missense_variant c.689C>T p.Thr230Ile
NC_007530	Chromosome	3208592	C	C	C	C	C	C	C	T	C	C	C	C	C	C	C	GBAA_RS16995	-	DUF3963 domain-containing protein	missense_variant c.34G>A p.Val12Ile
NC_007530	Chromosome	3215548	A	G	G	G	G	G	G	G	G	G	G	G	G	G	G	GBAA_RS17040	-	hypothetical protein	missense_variant c.55A>G p.Ile19Val
NC_007530	Chromosome	3217305	T	A	A	A	A	A	A	A	A	A	A	A	A	A	A	GBAA_RS17045	-	BcII family subclass B1 metallo-beta-lactamase	missense_variant c.425A>T p.Glu142Val
NC_007530	Chromosome	3222772	C	C	C	C	C	C	T	T	T	T	T	T	T	T	T	IGR	-	-	-
NC_007530	Chromosome	3226751	G	A	A	A	A	A	G	G	G	G	G	G	G	G	G	GBAA_RS30545	-	zinc-binding dehydrogenase	synonymous_variant c.201G>A p.Ala67Ala
NC_007530	Chromosome	3227122	A	G	G	G	G	G	G	G	G	G	G	G	G	G	G	GBAA_RS30545	-	zinc-binding dehydrogenase	stop_lost&splice_region_variant c.572A>G p.Ter191Trpext*?
NC_007530	Chromosome	3229291	G	T	T	T	T	G	G	G	G	G	G	G	G	G	G	IGR	-	-	-
NC_007530	Chromosome	3232712	C	C	C	C	C	C	A	A	A	A	A	A	A	A	A	IGR	-	-	-
NC_007530	Chromosome	3233071	A	A	A	A	A	A	A	A	A	T	T	T	T	T	A	IGR	-	-	-
NC_007530	Chromosome	3233824	A	C	C	C	C	C	C	C	C	C	C	C	C	C	C	IGR	-	-	-
NC_007530	Chromosome	3235332	G	G	G	G	G	G	A	A	A	A	A	A	A	A	A	GBAA_RS17170	-	membrane protein	missense_variant c.13G>A p.Ala5Thr
NC_007530	Chromosome	3248540	A	G	G	G	G	G	G	G	G	G	G	G	G	G	G	IGR	-	-	-
NC_007530	Chromosome	3249415	T	T	T	T	T	T	G	G	G	G	G	G	G	G	G	GBAA_RS17245	-	LysR family transcriptional regulator	missense_variant c.262A>C p.Ile88Leu
NC_007530	Chromosome	3249692	T	C	C	C	C	C	C	C	C	C	C	C	C	C	C	IGR	-	-	-
NC_007530	Chromosome	3251114	G	A	A	A	A	A	A	A	A	A	A	A	A	A	A	GBAA_RS17255	-	histidine phosphatase family protein	missense_variant c.422C>T p.Pro141Leu
NC_007530	Chromosome	3253204	A	G	G	G	G	G	A	A	A	A	A	A	A	A	A	GBAA_RS17265	-	mannonate oxidoreductase	synonymous_variant c.927T>C p.Ser309Ser
NC_007530	Chromosome	3253514	G	A	A	A	A	A	A	A	A	A	A	A	A	A	A	GBAA_RS17265	-	mannonate oxidoreductase	missense_variant c.617C>T p.Ala206Val
NC_007530	Chromosome	3256688	T	C	C	C	C	C	C	C	C	C	C	C	C	C	C	GBAA_RS17275	-	alpha-amylase	synonymous_variant c.321A>G p.Thr107Thr
NC_007530	Chromosome	3256870	T	C	C	C	C	C	C	C	C	C	C	C	C	C	C	GBAA_RS17275	-	alpha-amylase	missense_variant c.139A>G p.Arg47Gly
NC_007530	Chromosome	3261725	G	A	A	A	A	A	A	A	A	A	A	A	A	A	A	GBAA_RS17300	-	MFS transporter	synonymous_variant c.1164C>T p.Ser388Ser
NC_007530	Chromosome	3265469	A	G	G	G	G	G	G	G	G	G	G	G	G	G	G	GBAA_RS17320	-	glycerophosphodiester phosphodiesterase	synonymous_variant c.654T>C p.Asn218Asn
NC_007530	Chromosome	3273849	C	T	T	T	T	T	C	C	C	C	C	C	C	C	C	IGR	-	-	-
NC_007530	Chromosome	3273856	T	C	C	C	C	C	T	T	T	T	T	T	T	T	T	IGR	-	-	-
NC_007530	Chromosome	3304654	A	A	A	A	A	A	G	G	G	G	G	G	G	G	G	GBAA_RS17520	-	hypothetical protein	synonymous_variant c.6069T>C p.Gly2023Gly
NC_007530	Chromosome	3306423	C	T	T	T	T	T	T	T	T	T	T	T	T	T	T	GBAA_RS17520	-	hypothetical protein	missense_variant c.4300G>A p.Ala1434Thr
NC_007530	Chromosome	3310574	G	A	A	A	A	A	A	A	A	A	A	A	A	A	A	GBAA_RS17520	-	hypothetical protein	missense_variant c.149C>T p.Ala50Val
NC_007530	Chromosome	3320108	G	A	A	A	A	A	A	A	A	A	A	A	A	A	A	GBAA_RS17565	-	3-oxoacyl-ACP reductase FabG	missense_variant c.572C>T p.Thr191Met
NC_007530	Chromosome	3328983	G	A	A	A	A	A	A	A	A	A	A	A	A	A	A	GBAA_RS17625	-	thiamine/molybdopterin biosynthesis protein MoeB	synonymous_variant c.783C>T p.Ala261Ala
NC_007530	Chromosome	3332283	G	A	A	A	A	A	G	G	G	G	G	G	G	G	G	GBAA_RS17640	-	sulfurtransferase FdhD	missense_variant c.311C>T p.Ser104Leu
NC_007530	Chromosome	3337953	G	A	A	A	A	A	A	A	A	A	A	A	A	A	A	GBAA_RS17665	-	spore germination protein	missense_variant c.1153G>A p.Val385Ile
NC_007530	Chromosome	3358576	A	A	A	A	A	A	A	C	A	A	A	A	A	A	A	IGR	-	-	-
NC_007530	Chromosome	3359543	G	G	G	G	G	G	T	T	T	T	T	T	T	T	T	GBAA_RS17755	-	ABC transporter permease	synonymous_variant c.115C>A p.Arg39Arg
NC_007530	Chromosome	3366432	T	C	C	C	C	C	T	T	T	T	T	T	T	T	T	GBAA_RS17785	-	DNA topoisomerase IV subunit B	synonymous_variant c.663A>G p.Thr221Thr
NC_007530	Chromosome	3366937	G	G	G	G	G	G	A	G	G	G	G	G	G	G	G	GBAA_RS17785	-	DNA topoisomerase IV subunit B	missense_variant c.158C>T p.Ala53Val
NC_007530	Chromosome	3375491	T	T	T	T	T	T	C	C	C	C	C	C	C	C	C	IGR	-	-	-
NC_007530	Chromosome	3376128	G	A	A	A	A	A	G	G	G	G	G	G	G	G	G	IGR	-	-	-
NC_007530	Chromosome	3380414	G	A	A	A	A	A	A	A	A	A	A	A	A	A	A	GBAA_RS17875	acnA	aconitate hydratase AcnA	synonymous_variant c.2511C>T p.Leu837Leu
NC_007530	Chromosome	3382079	C	T	T	T	T	T	T	T	T	T	T	T	T	T	T	GBAA_RS17875	acnA	aconitate hydratase AcnA	synonymous_variant c.846G>A p.Val282Val
NC_007530	Chromosome	3386925	A	G	G	G	G	G	G	G	G	G	G	G	G	G	G	GBAA_RS17915	-	alanyl-tRNA editing protein	missense_variant c.841A>G p.Lys281Glu
NC_007530	Chromosome	3395816	T	A	A	A	A	A	A	A	A	A	A	A	A	A	A	GBAA_RS17965	-	S-layer protein	missense_variant c.1498A>T p.Asn500Tyr
NC_007530	Chromosome	3398818	G	A	A	A	A	A	A	A	A	A	A	A	A	A	A	GBAA_RS17980	-	N-acetylmuramoyl-L-alanine amidase	missense_variant c.95_97delTACinsGAT p.ValArg32GlyTrp
NC_007530	Chromosome	3398820	A	C	C	C	C	C	C	C	C	C	C	C	C	C	C	GBAA_RS17980	-	N-acetylmuramoyl-L-alanine amidase	missense_variant c.95_97delTACinsGAT p.ValArg32GlyTrp
NC_007530	Chromosome	3428068	C	T	T	T	T	T	T	T	T	T	T	T	T	T	T	GBAA_RS18110	-	hypothetical protein	synonymous_variant c.5493C>T p.Thr1831Thr
NC_007530	Chromosome	3441042	C	C	C	C	C	C	C	C	T	T	T	T	T	T	T	GBAA_RS18180	-	ABC transporter ATP-binding protein	missense_variant c.29G>A p.Arg10Lys
NC_007530	Chromosome	3451711	T	T	T	T	T	T	C	C	C	C	C	C	C	C	C	GBAA_RS30620	-	bifunctional metallophosphatase/5'-nucleotidase	missense_variant c.484T>C p.Trp162Arg
NC_007530	Chromosome	3455152	A	G	G	G	G	G	G	G	G	G	G	G	G	G	G	GBAA_RS18260	-	GNAT family acetyltransferase	missense_variant c.296T>C p.Val99Ala
NC_007530	Chromosome	3459304	T	T	T	T	T	T	C	C	C	C	C	C	C	C	C	IGR	-	-	-
NC_007530	Chromosome	3460779	T	C	C	C	C	C	C	C	C	C	C	C	C	C	C	GBAA_RS18300	-	hypothetical protein	missense_variant c.23A>G p.Asp8Gly
NC_007530	Chromosome	3486001	T	C	C	C	C	C	C	C	C	C	C	C	C	C	C	IGR	-	-	-
NC_007530	Chromosome	3520116	C	C	C	C	C	C	G	G	G	G	G	G	G	G	G	GBAA_RS18680	-	PTS fructose transporter subunit IIC	missense_variant c.980G>C p.Gly327Ala
NC_007530	Chromosome	3527275	G	G	G	G	G	G	A	A	A	A	A	A	A	A	A	IGR	-	-	-
NC_007530	Chromosome	3532585	T	C	C	C	C	C	C	C	C	C	C	C	C	C	C	GBAA_RS18740	-	copper-translocating P-type ATPase	missense_variant c.827A>G p.Asp276Gly
NC_007530	Chromosome	3553572	G	G	G	G	G	G	A	A	A	A	A	A	A	A	A	GBAA_RS18860	-	LrgB family protein	stop_gained c.217C>T p.Arg73*
NC_007530	Chromosome	3560815	G	A	A	A	A	A	A	A	A	A	A	A	A	A	A	IGR	-	-	-
NC_007530	Chromosome	3566634	G	G	G	G	G	G	G	G	A	A	A	A	A	A	A	GBAA_RS18915	-	GTP-binding protein	missense_variant c.2021C>T p.Pro674Leu
NC_007530	Chromosome	3568849	A	G	G	G	G	G	G	G	G	G	G	G	G	G	G	IGR	-	-	-
NC_007530	Chromosome	3574302	T	C	C	C	T	T	T	T	T	T	T	T	T	T	T	GBAA_RS18945	-	bacteriocin ABC transporter permease	synonymous_variant c.102A>G p.Leu34Leu
NC_007530	Chromosome	3591116	A	G	G	G	G	G	G	G	G	G	G	G	G	G	G	IGR	-	-	-
NC_007530	Chromosome	3600786	C	T	T	T	T	T	T	T	T	T	T	T	T	T	T	GBAA_RS19065	-	ABC transporter permease	missense_variant c.481G>A p.Ala161Thr
NC_007530	Chromosome	3603639	G	G	G	G	G	G	G	G	A	A	A	A	A	A	A	GBAA_RS19080	-	BMP family ABC transporter substrate-binding protein	missense_variant c.638C>T p.Ala213Val
NC_007530	Chromosome	3604359	A	A	A	A	A	A	G	G	G	G	G	G	G	G	G	IGR	-	-	-
NC_007530	Chromosome	3606981	C	C	C	C	C	C	T	C	C	C	C	C	C	C	C	GBAA_RS19090	-	DNA translocase FtsK	missense_variant c.1030G>A p.Glu344Lys
NC_007530	Chromosome	3621380	T	C	C	C	C	C	C	C	C	C	C	C	C	C	C	GBAA_RS19155	pnp	polyribonucleotide nucleotidyltransferase	missense_variant c.344A>G p.Asn115Ser
NC_007530	Chromosome	3633495	T	T	T	T	T	T	C	C	C	C	C	C	C	C	C	GBAA_RS19210	polC	PolC-type DNA polymerase III	synonymous_variant c.324A>G p.Ser108Ser
NC_007530	Chromosome	3635951	A	G	G	G	G	G	A	A	A	A	A	A	A	A	A	GBAA_RS19220	rseP	RIP metalloprotease RseP	synonymous_variant c.1065T>C p.Ser355Ser
NC_007530	Chromosome	3640398	A	G	G	G	G	G	G	G	G	G	G	G	G	G	G	GBAA_RS19240	-	ribosome-recycling factor	missense_variant c.23T>C p.Phe8Ser
NC_007530	Chromosome	3650442	T	T	T	T	T	T	T	T	T	T	T	T	C	T	T	GBAA_RS19285	-	DNA topoisomerase 1	missense_variant c.67A>G p.Lys23Glu
NC_007530	Chromosome	3653651	A	G	G	G	G	G	G	G	G	G	G	G	G	G	G	GBAA_RS19300	-	succinyl-CoA ligase subunit beta	synonymous_variant c.42T>C p.Ser14Ser
NC_007530	Chromosome	3657644	A	T	T	T	T	T	A	A	A	A	A	A	A	A	A	GBAA_RS19330	-	ribosome maturation factor RimM	synonymous_variant c.372T>A p.Val124Val
NC_007530	Chromosome	3667929	A	G	G	G	G	G	G	G	G	G	G	G	G	G	G	GBAA_RS19380	fabD	[acyl-carrier-protein] S-malonyltransferase	missense_variant c.154T>C p.Ser52Pro
NC_007530	Chromosome	3669027	T	C	C	C	C	C	T	T	T	T	T	T	T	T	T	GBAA_RS19385	-	phosphate acyltransferase	synonymous_variant c.63A>G p.Ala21Ala
NC_007530	Chromosome	3674768	C	C	C	C	C	C	T	T	T	T	T	T	T	T	T	IGR	-	-	-
NC_007530	Chromosome	3682161	C	C	C	C	C	C	T	T	T	T	T	T	T	T	T	GBAA_RS19450	-	16S rRNA (cytosine(967)-C(5))-methyltransferase RsmB	synonymous_variant c.537G>A p.Ala179Ala
NC_007530	Chromosome	3682373	G	G	G	G	G	G	A	A	A	A	A	A	A	A	A	GBAA_RS19450	-	16S rRNA (cytosine(967)-C(5))-methyltransferase RsmB	missense_variant c.325C>T p.His109Tyr
NC_007530	Chromosome	3684073	T	T	T	T	T	T	C	C	C	C	C	C	C	C	C	GBAA_RS19460	-	peptide deformylase	synonymous_variant c.60A>G p.Arg20Arg
NC_007530	Chromosome	3688083	C	T	T	T	T	T	T	T	T	T	T	T	T	T	T	GBAA_RS19475	-	DNA-directed RNA polymerase subunit omega	synonymous_variant c.54G>A p.Thr18Thr
NC_007530	Chromosome	3696168	C	T	T	T	T	T	T	T	T	T	T	T	T	T	T	GBAA_RS19510	-	membrane protein	missense_variant c.331G>A p.Ala111Thr
NC_007530	Chromosome	3697894	T	T	T	T	T	T	T	T	C	C	C	C	C	C	C	GBAA_RS19520	-	membrane protein	synonymous_variant c.849T>C p.Ser283Ser
NC_007530	Chromosome	3712048	T	C	C	C	C	C	C	C	C	C	C	C	C	C	C	GBAA_RS19585	-	RluA family pseudouridine synthase	missense_variant c.292A>G p.Ser98Gly
NC_007530	Chromosome	3714629	G	G	G	G	G	G	A	A	A	A	A	A	A	A	A	GBAA_RS19600	-	isoleucine--tRNA ligase	missense_variant c.1549C>T p.Arg517Cys
NC_007530	Chromosome	3726311	A	G	G	G	G	G	G	G	G	G	G	G	G	G	G	IGR	-	-	-
NC_007530	Chromosome	3729934	G	A	A	A	A	A	A	A	A	A	A	A	A	A	A	GBAA_RS19680	-	stage V sporulation protein E	missense_variant c.724C>T p.Leu242Phe
NC_007530	Chromosome	3740385	A	A	A	A	A	A	G	G	G	G	G	G	G	G	G	IGR	-	-	-
NC_007530	Chromosome	3755462	C	T	T	T	T	T	T	T	T	T	T	T	T	T	T	GBAA_RS19815	-	hypothetical protein	missense_variant c.1471G>A p.Val491Ile
NC_007530	Chromosome	3759429	G	A	A	A	A	A	A	A	A	A	A	A	A	A	A	GBAA_RS19830	-	phage tail tape measure protein	synonymous_variant c.4032C>T p.Leu1344Leu
NC_007530	Chromosome	3764315	A	T	T	T	T	T	A	A	A	A	A	A	A	A	A	GBAA_RS19845	-	tail protein	missense_variant c.358T>A p.Tyr120Asn
NC_007530	Chromosome	3778287	G	G	G	G	G	G	A	A	A	A	A	A	A	A	A	GBAA_RS30710	-	hypothetical protein	missense_variant c.37G>A p.Glu13Lys
NC_007530	Chromosome	3785996	A	G	G	G	G	G	G	G	G	G	G	G	G	G	G	GBAA_RS30720	-	hypothetical protein	missense_variant c.32T>C p.Ile11Thr
NC_007530	Chromosome	3788654	A	G	G	G	G	G	G	G	G	G	G	G	G	G	G	IGR	-	-	-
NC_007530	Chromosome	3797251	C	T	T	T	T	T	T	T	T	T	T	T	T	T	T	IGR	-	-	-
NC_007530	Chromosome	3798765	A	G	G	G	G	G	G	G	G	G	G	G	G	G	G	IGR	-	-	-
NC_007530	Chromosome	3811489	T	T	T	T	T	T	C	C	C	C	C	C	C	C	C	GBAA_RS20190	pyc	pyruvate carboxylase	synonymous_variant c.1065A>G p.Pro355Pro
NC_007530	Chromosome	3822521	G	A	A	A	A	A	A	A	A	A	A	A	A	A	A	GBAA_RS20260	-	hypothetical protein	missense_variant c.256C>T p.Leu86Phe
NC_007530	Chromosome	3833407	A	G	G	G	G	G	A	A	A	A	A	A	A	A	A	GBAA_RS20330	-	peptide deformylase	missense_variant c.212A>G p.Lys71Arg
NC_007530	Chromosome	3838778	G	A	A	A	A	A	G	G	G	G	G	G	G	G	G	IGR	-	-	-
NC_007530	Chromosome	3842864	G	A	A	A	A	A	A	A	A	A	A	A	A	A	A	GBAA_RS20385	-	hypothetical protein	missense_variant c.508C>T p.His170Tyr
NC_007530	Chromosome	3849500	G	G	G	G	G	G	A	A	A	A	A	A	A	A	A	GBAA_RS20430	-	polyphosphate kinase	missense_variant c.1754C>T p.Pro585Leu
NC_007530	Chromosome	3850098	T	T	T	T	T	T	C	C	C	C	C	C	C	C	C	GBAA_RS20430	-	polyphosphate kinase	missense_variant c.1156A>G p.Thr386Ala
NC_007530	Chromosome	3851344	C	T	T	T	T	T	T	T	T	T	T	T	T	T	T	IGR	-	-	-
NC_007530	Chromosome	3864276	C	T	T	T	T	T	T	T	T	T	T	T	T	T	T	GBAA_RS20505	-	sensor histidine kinase	missense_variant c.891G>A p.Met297Ile
NC_007530	Chromosome	3871950	G	G	G	G	G	G	A	A	A	A	A	A	A	A	A	IGR	-	-	-
NC_007530	Chromosome	3886059	C	C	C	C	C	C	T	T	T	T	T	T	T	T	T	IGR	-	-	-
NC_007530	Chromosome	3889072	T	C	C	C	C	C	C	C	C	C	C	C	C	C	C	GBAA_RS20645	mtnA	S-methyl-5-thioribose-1-phosphate isomerase	synonymous_variant c.384A>G p.Ala128Ala
NC_007530	Chromosome	3891372	G	G	G	G	G	G	A	A	A	A	A	A	A	A	A	GBAA_RS20655	-	carbon-nitrogen family hydrolase	missense_variant c.346C>T p.Leu116Phe
NC_007530	Chromosome	3891831	C	C	C	C	C	C	A	A	A	A	A	A	A	A	A	GBAA_RS20660	-	pyridoxal phosphate-dependent aminotransferase	missense_variant c.17C>A p.Pro6His
NC_007530	Chromosome	3896694	G	A	A	A	A	A	A	A	A	A	A	A	A	A	A	GBAA_RS20685	-	hypothetical protein	missense_variant c.133G>A p.Gly45Ser
NC_007530	Chromosome	3900972	G	G	G	G	G	G	A	A	A	A	A	A	A	A	A	IGR	-	-	-
NC_007530	Chromosome	3906109	G	A	A	A	A	A	A	A	A	A	A	A	A	A	A	IGR	-	-	-
NC_007530	Chromosome	3909956	T	T	T	T	T	T	T	T	C	C	C	C	C	C	C	GBAA_RS20755	nagA	N-acetylglucosamine-6-phosphate deacetylase	synonymous_variant c.975A>G p.Ser325Ser
NC_007530	Chromosome	3911649	A	A	A	A	A	A	A	A	A	A	A	A	A	G	A	GBAA_RS20760	-	MBL fold metallo-hydrolase	synonymous_variant c.393T>C p.Ser131Ser
NC_007530	Chromosome	3916961	A	G	G	G	G	G	G	G	G	G	G	G	G	G	G	GBAA_RS20810	-	Cof-type HAD-IIB family hydrolase	missense_variant c.680T>C p.Leu227Pro
NC_007530	Chromosome	3923507	A	G	G	G	G	G	G	G	G	G	G	G	G	G	G	GBAA_RS20855	-	sodium-dependent transporter	missense_variant c.908T>C p.Val303Ala
NC_007530	Chromosome	3939537	A	A	A	A	A	A	G	G	G	G	G	G	G	G	G	GBAA_RS20945	-	site-specific tyrosine recombinase XerD	missense_variant c.443T>C p.Val148Ala
NC_007530	Chromosome	3941817	T	C	C	C	C	C	C	C	C	C	C	C	C	C	C	GBAA_RS20965	-	N-acetyltransferase	synonymous_variant c.162T>C p.Ser54Ser
NC_007530	Chromosome	3947965	C	T	T	T	T	T	T	T	T	T	T	T	T	T	T	GBAA_RS21005	-	ribbon-helix-helix domain-containing protein	missense_variant c.211G>A p.Ala71Thr
NC_007530	Chromosome	3949024	A	A	A	A	A	A	A	A	T	T	T	T	T	T	T	GBAA_RS21010	-	alpha/beta hydrolase	missense_variant c.728A>T p.Asn243Ile
NC_007530	Chromosome	3949038	G	G	G	G	G	G	G	G	A	A	A	A	A	A	A	GBAA_RS21010	-	alpha/beta hydrolase	missense_variant c.742G>A p.Val248Ile
NC_007530	Chromosome	3951143	G	G	G	G	G	G	C	C	C	C	C	C	C	C	C	GBAA_RS21020	-	LacI family transcriptional regulator	missense_variant c.491C>G p.Ala164Gly
NC_007530	Chromosome	3960277	C	T	T	T	T	T	C	C	C	C	C	C	C	C	C	GBAA_RS21080	-	alpha/beta hydrolase	missense_variant c.661G>A p.Glu221Lys
NC_007530	Chromosome	3973911	A	G	G	G	G	G	G	G	G	G	G	G	G	G	G	GBAA_RS21145	argB	acetylglutamate kinase	missense_variant c.737T>C p.Met246Thr
NC_007530	Chromosome	3978791	T	C	C	C	C	C	T	T	T	T	T	T	T	T	T	GBAA_RS21170	-	hydrolase	missense_variant c.337A>G p.Asn113Asp
NC_007530	Chromosome	3981640	A	G	G	G	G	G	G	G	G	G	G	G	G	G	G	GBAA_RS21190	sdaAB	L-serine ammonia-lyase, iron-sulfur-dependent, subunit beta	synonymous_variant c.99T>C p.Cys33Cys
NC_007530	Chromosome	3985018	G	A	A	A	A	A	A	A	A	A	A	A	A	A	A	GBAA_RS21210	polYA	DNA polymerase IV	stop_gained c.289C>T p.Gln97*
NC_007530	Chromosome	3987434	A	G	G	G	G	G	G	G	G	G	G	G	G	G	G	GBAA_RS21225	-	N-acetyltransferase	missense_variant c.50T>C p.Leu17Ser
NC_007530	Chromosome	3992142	G	A	A	A	A	A	A	A	A	A	A	A	A	A	A	GBAA_RS30780	-	hypothetical protein	missense_variant c.55C>T p.Arg19Trp
NC_007530	Chromosome	4005648	G	A	A	A	A	A	A	A	A	A	A	A	A	A	A	GBAA_RS21330	-	sigma-54-dependent Fis family transcriptional regulator	missense_variant c.95C>T p.Thr32Ile
NC_007530	Chromosome	4032215	G	G	G	G	G	G	G	G	A	A	A	A	A	A	A	GBAA_RS21495	-	aminopeptidase P family protein	missense_variant c.416C>T p.Ala139Val
NC_007530	Chromosome	4032507	T	T	T	T	T	T	C	C	C	C	C	C	C	C	C	GBAA_RS21495	-	aminopeptidase P family protein	missense_variant c.124A>G p.Ile42Val
NC_007530	Chromosome	4032981	T	C	C	C	C	C	C	C	C	C	C	C	C	C	C	GBAA_RS21500	aroQ	3-dehydroquinate dehydratase	missense_variant c.95A>G p.Glu32Gly
NC_007530	Chromosome	4034659	C	C	C	C	C	C	T	T	T	T	T	T	T	T	T	GBAA_RS21510	-	DUF1385 domain-containing protein	missense_variant c.799C>T p.Arg267Cys
NC_007530	Chromosome	4036979	G	G	G	G	G	G	A	A	A	A	A	A	A	A	A	GBAA_RS21530	splB	spore photoproduct lyase	missense_variant c.595C>T p.Arg199Cys
NC_007530	Chromosome	4037455	A	G	G	G	G	G	G	G	G	G	G	G	G	G	G	GBAA_RS21530	splB	spore photoproduct lyase	missense_variant c.119T>C p.Phe40Ser
NC_007530	Chromosome	4042504	A	G	G	G	G	G	G	G	G	G	G	G	G	G	G	GBAA_RS21565	-	QacE family quaternary ammonium compound efflux SMR transporter	missense_variant c.82A>G p.Ser28Gly
NC_007530	Chromosome	4042912	C	T	T	T	T	T	T	T	T	T	T	T	T	T	T	GBAA_RS21570	-	DUF3975 domain-containing protein	missense_variant c.160C>T p.Arg54Trp
NC_007530	Chromosome	4047763	C	T	T	T	T	T	T	T	T	T	T	T	T	T	T	GBAA_RS21620	-	DUF3929 domain-containing protein	missense_variant c.112_113delAGinsCA p.Ser38His
NC_007530	Chromosome	4047764	T	G	G	G	G	G	G	G	G	G	G	G	G	G	G	GBAA_RS21620	-	DUF3929 domain-containing protein	missense_variant c.112_113delAGinsCA p.Ser38His
NC_007530	Chromosome	4098955	T	C	C	C	C	C	T	T	T	T	T	T	T	T	T	GBAA_RS21915	-	zinc ABC transporter ATP-binding protein	missense_variant c.647A>G p.Gln216Arg
NC_007530	Chromosome	4099784	C	G	G	G	G	G	C	C	C	C	C	C	C	C	C	IGR	-	-	-
NC_007530	Chromosome	4116426	A	G	G	G	G	G	A	A	A	A	A	A	A	A	A	GBAA_RS22015	-	diacylglycerol kinase	missense_variant c.182T>C p.Val61Ala
NC_007530	Chromosome	4126642	T	T	T	T	T	T	C	C	C	C	C	C	C	C	C	GBAA_RS22075	-	molecular chaperone DnaJ	missense_variant c.808A>G p.Ile270Val
NC_007530	Chromosome	4129809	G	A	A	A	A	A	A	A	A	A	A	A	A	A	A	GBAA_RS22085	-	nucleotide exchange factor GrpE	missense_variant c.274C>T p.Pro92Ser
NC_007530	Chromosome	4134667	T	C	C	C	C	C	C	C	C	C	C	C	C	C	C	GBAA_RS22105	-	elongation factor 4	missense_variant c.202A>G p.Ile68Val
NC_007530	Chromosome	4135260	A	G	G	G	G	G	G	G	G	G	G	G	G	G	G	GBAA_RS22110	-	DUF3679 domain-containing protein	missense_variant c.182T>C p.Val61Ala
NC_007530	Chromosome	4145299	C	T	T	T	T	T	T	T	T	T	T	T	T	T	T	GBAA_RS22170	-	nicotinate-nicotinamide nucleotide adenylyltransferase	missense_variant c.145G>A p.Asp49Asn
NC_007530	Chromosome	4146896	T	G	G	G	G	G	T	T	T	T	T	T	T	T	T	GBAA_RS22185	-	ribosome biogenesis GTPase YqeH	synonymous_variant c.933A>C p.Pro311Pro
NC_007530	Chromosome	4147376	C	T	T	T	T	T	T	T	T	T	T	T	T	T	T	GBAA_RS22185	-	ribosome biogenesis GTPase YqeH	synonymous_variant c.453G>A p.Ala151Ala
NC_007530	Chromosome	4149771	T	C	C	C	C	C	C	C	C	C	C	C	C	C	C	IGR	-	-	-
NC_007530	Chromosome	4158968	C	T	T	T	T	T	T	T	T	T	T	T	T	T	T	GBAA_RS22250	-	alpha/beta hydrolase	synonymous_variant c.618C>T p.Asp206Asp

NC_007530	Chromosome	4168478 G	A	A	A	A	A	A	A	A	A	A	A	A	A	A	GBAA_RS22290	-	phenylalanine 4-monooxygenase	synonymous_variant c.1578G>A p.Leu526Leu
NC_007530	Chromosome	4189384 A	G	G	G	G	G	G	G	G	G	G	G	G	G	G	GBAA_RS22415	-	collagenase-like protease	missense_variant c.71T>C p.Val24Ala
NC_007530	Chromosome	4198007 A	G	G	G	G	G	G	G	G	G	G	G	G	G	G	IGR	-	-	-
NC_007530	Chromosome	4209746 C	C	C	C	C	C	C	T	C	C	C	C	C	C	C	GBAA_RS22520	-	histidine--tRNA ligase	missense_variant c.1021G>A p.Ala341Thr
NC_007530	Chromosome	4221322 C	T	T	T	T	T	T	T	T	T	T	T	T	T	T	IGR	-	-	-
NC_007530	Chromosome	4221445 G	A	A	A	A	A	A	A	A	A	A	A	A	A	A	GBAA_RS22565	spoVB	stage V sporulation protein B	synonymous_variant c.69G>A p.Leu23Leu
NC_007530	Chromosome	4234105 A	G	G	G	G	G	G	G	G	G	G	G	G	G	G	GBAA_RS22645	-	peptidoglycan-binding protein LysM	synonymous_variant c.978T>C p.Asn326Asn
NC_007530	Chromosome	4234117 C	T	T	T	T	T	T	T	T	T	T	T	T	T	T	GBAA_RS22645	-	peptidoglycan-binding protein LysM	synonymous_variant c.966G>A p.Val322Val
NC_007530	Chromosome	4234333 C	C	C	C	C	C	T	T	T	T	T	T	T	T	T	GBAA_RS22645	-	peptidoglycan-binding protein LysM	synonymous_variant c.750G>A p.Gly250Gly
NC_007530	Chromosome	4234339 A	A	A	A	A	A	G	G	G	G	G	G	G	G	G	GBAA_RS22645	-	peptidoglycan-binding protein LysM	synonymous_variant c.744T>C p.Asn248Asn
NC_007530	Chromosome	4238099 C	T	T	T	T	T	T	T	T	T	T	T	T	T	T	GBAA_RS22660	-	L-aspartate oxidase	missense_variant c.878G>A p.Gly293Glu
NC_007530	Chromosome	4241491 A	G	G	G	G	G	G	G	G	G	G	G	G	G	G	GBAA_RS22675	-	MOSC domain-containing protein	missense_variant c.598A>G p.Thr200Ala
NC_007530	Chromosome	4242237 T	T	T	T	T	T	C	C	C	C	C	C	C	C	C	GBAA_RS22680	-	prephenate dehydratase	missense_variant c.238A>G p.Thr80Ala
NC_007530	Chromosome	4264640 A	G	G	G	G	G	A	A	A	A	A	A	A	A	A	GBAA_RS22800	-	valine--tRNA ligase	synonymous_variant c.1641T>C p.Asp547Asp
NC_007530	Chromosome	4268630 G	A	A	A	A	A	G	G	G	G	G	G	G	G	G	GBAA_RS22810	spoVID	stage VI sporulation protein D	missense_variant c.118C>T p.Leu40Phe
NC_007530	Chromosome	4277562 G	G	G	G	G	G	A	A	A	A	A	A	A	A	A	GBAA_RS22860	lon	endopeptidase La	synonymous_variant c.1722C>T p.Asn574Asn
NC_007530	Chromosome	4297311 T	T	T	T	T	T	C	C	C	C	C	C	C	C	C	GBAA_RS22970	-	xanthine permease	synonymous_variant c.963A>G p.Leu321Leu
NC_007530	Chromosome	4305347 G	G	G	G	G	G	A	A	A	A	A	A	A	A	A	GBAA_RS23000	-	ABC transporter permease	missense_variant c.674G>A p.Arg225Lys
NC_007530	Chromosome	4306492 A	G	G	G	G	G	G	G	G	G	G	G	G	G	G	GBAA_RS23005	-	ABC transporter ATP-binding protein	missense_variant c.884A>G p.Tyr295Cys
NC_007530	Chromosome	4308584 T	T	T	T	T	T	C	C	C	C	C	C	C	C	C	GBAA_RS23020	-	DNA-binding response regulator	missense_variant c.133T>C p.Phe45Leu
NC_007530	Chromosome	4312115 G	G	G	G	G	G	A	A	A	A	A	A	A	A	A	GBAA_RS23045	-	multidrug resistance efflux transporter family protein	missense_variant c.508G>A p.Val170Ile
NC_007530	Chromosome	4332170 T	T	T	T	T	T	C	C	C	C	C	C	C	C	C	IGR	-	-	-
NC_007530	Chromosome	4345784 C	T	T	T	T	T	T	T	T	T	T	T	T	T	T	GBAA_RS23225	-	sodium/hydrogen exchanger	synonymous_variant c.594G>A p.Val198Val
NC_007530	Chromosome	4346405 A	A	A	A	A	A	G	G	G	G	G	G	G	G	G	IGR	-	-	-
NC_007530	Chromosome	4348916 A	G	G	G	G	G	G	G	G	G	G	G	G	G	G	GBAA_RS23245	-	iron ABC transporter permease	missense_variant c.701T>C p.Val234Ala
NC_007530	Chromosome	4367488 A	A	A	A	A	A	A	A	G	G	G	G	G	G	G	GBAA_RS23330	-	phenylalanine--tRNA ligase subunit beta	missense_variant c.1396T>C p.Ser466Pro
NC_007530	Chromosome	4374460 T	C	C	C	C	C	T	T	T	T	T	T	T	T	T	GBAA_RS23370	-	hypothetical protein	synonymous_variant c.231T>C p.Ser77Ser
NC_007530	Chromosome	4378129 G	A	A	A	A	A	A	A	A	A	A	A	A	A	A	GBAA_RS23390	-	peptidase M28	missense_variant c.986C>T p.Ala329Val
NC_007530	Chromosome	4383167 C	C	C	C	C	C	T	C	C	C	C	C	C	C	C	GBAA_RS23415	-	threonine--tRNA ligase	missense_variant c.430G>A p.Val144Ile
NC_007530	Chromosome	4387106 G	G	G	G	G	G	A	A	A	A	A	A	A	A	A	GBAA_RS23430	-	Replication initiation and membrane attachment protein	missense_variant c.338C>T p.Pro113Leu
NC_007530	Chromosome	4389091 T	C	C	C	C	C	T	T	T	T	T	T	T	T	T	GBAA_RS23445	-	glyceraldehyde-3-phosphate dehydrogenase	splice_region_variant&stop_retained_variant c.1029A>G p.Ter343Ter
NC_007530	Chromosome	4398276 T	A	A	A	A	A	A	A	A	A	A	A	A	A	A	IGR	-	-	-
NC_007530	Chromosome	4404737 G	A	A	A	A	A	A	A	A	A	A	A	A	A	A	GBAA_RS23515	ytvI	sporulation integral membrane protein YtvI	missense_variant c.715G>A p.Val239Ile
NC_007530	Chromosome	4420186 G	T	T	T	T	T	T	T	T	T	T	T	T	T	T	GBAA_RS23590	-	thioesterase	missense_variant c.1120C>A p.Gln374Lys
NC_007530	Chromosome	4422198 A	T	T	T	T	T	A	A	A	A	A	A	A	A	A	GBAA_RS23600	-	metal-dependent hydrolase	missense_variant c.238T>A p.Trp80Arg
NC_007530	Chromosome	4422904 T	A	A	A	A	A	A	A	A	A	A	A	A	A	A	GBAA_RS23605	-	aminopeptidase P family protein	missense_variant c.320T>A p.Leu107His
NC_007530	Chromosome	4431774 G	A	A	A	A	A	A	A	A	A	A	A	A	A	A	GBAA_RS23675	-	universal stress protein	missense_variant c.145G>A p.Ala49Thr
NC_007530	Chromosome	4435406 A	A	A	A	A	A	G	G	G	G	G	G	G	G	G	GBAA_RS23700	-	argininosuccinate synthase	synonymous_variant c.783T>C p.Arg261Arg
NC_007530	Chromosome	4437487 T	A	A	A	A	A	T	T	T	T	T	T	T	T	T	IGR	-	-	-
NC_007530	Chromosome	4455720 A	G	G	G	G	G	A	A	A	A	A	A	A	A	A	IGR	-	-	-
NC_007530	Chromosome	4461029 T	C	C	C	C	C	T	T	T	T	T	T	T	T	T	GBAA_RS23840	-	Tyrosine--tRNA ligase 1	synonymous_variant c.1236A>G p.Lys412Lys
NC_007530	Chromosome	4478494 G	G	G	G	G	G	A	A	A	A	A	A	A	A	A	GBAA_RS23950	-	DUF948 domain-containing protein	missense_variant c.119C>T p.Thr40Met
NC_007530	Chromosome	4487165 T	C	C	C	C	C	C	C	C	C	C	C	C	C	C	GBAA_RS23990	-	DNA translocase FtsK	missense_variant c.1338_1342delAGAAAinsGGAAG p.Thr448Ala
NC_007530	Chromosome	4487169 T	C	C	C	C	C	C	C	C	C	C	C	C	C	C	GBAA_RS23990	-	DNA translocase FtsK	missense_variant c.1338_1342delAGAAAinsGGAAG p.Thr448Ala
NC_007530	Chromosome	4487358 C	C	C	C	C	C	T	T	T	T	T	T	T	T	T	GBAA_RS23990	-	DNA translocase FtsK	synonymous_variant c.1149G>A p.Ala383Ala
NC_007530	Chromosome	4487452 G	G	G	G	G	G	A	A	A	A	A	A	A	A	A	GBAA_RS23990	-	DNA translocase FtsK	missense_variant c.1055C>T p.Pro352Leu
NC_007530	Chromosome	4488304 T	T	T	T	T	T	T	C	T	T	T	T	T	T	T	GBAA_RS23990	-	DNA translocase FtsK	missense_variant c.203A>G p.Asp68Gly
NC_007530	Chromosome	4488395 G	A	A	A	A	A	A	A	A	A	A	A	A	A	A	GBAA_RS23990	-	DNA translocase FtsK	missense_variant c.112C>T p.His38Tyr
NC_007530	Chromosome	4489311 A	G	G	G	G	G	G	G	G	G	G	G	G	G	G	IGR	-	-	-
NC_007530	Chromosome	4489312 C	T	T	T	T	T	T	T	T	T	T	T	T	T	T	IGR	-	-	-
NC_007530	Chromosome	4489314 G	A	A	A	A	A	A	A	A	A	A	A	A	A	A	IGR	-	-	-
NC_007530	Chromosome	4489315 T	C	C	C	C	C	C	C	C	C	C	C	C	C	C	IGR	-	-	-
NC_007530	Chromosome	4489321 T	G	G	G	G	G	G	G	G	G	G	G	G	G	G	IGR	-	-	-
NC_007530	Chromosome	4493218 T	C	C	C	C	C	C	C	C	C	C	C	C	C	C	GBAA_RS24020	-	peptidase M28	synonymous_variant c.15A>G p.Thr5Thr
NC_007530	Chromosome	4495586 C	C	C	C	C	C	C	A	C	C	C	C	C	C	C	IGR	-	-	-
NC_007530	Chromosome	4499321 C	A	A	A	A	A	A	A	A	A	A	A	A	A	A	GBAA_RS24055	-	hypothetical protein	missense_variant c.4G>T p.Asp2Tyr
NC_007530	Chromosome	4502060 T	T	T	T	T	T	C	C	C	C	C	C	C	C	C	GBAA_RS24080	-	rRNA pseudouridine synthase	missense_variant c.710A>G p.Gln237Arg
NC_007530	Chromosome	4505929 T	T	T	T	T	T	C	C	C	C	C	C	C	C	C	GBAA_RS24090	-	NAD(P)/FAD-dependent oxidoreductase	synonymous_variant c.1212T>C p.Ala404Ala
NC_007530	Chromosome	4506294 G	G	G	G	G	G	A	A	A	A	A	A	A	A	A	GBAA_RS24095	-	MFS transporter	missense_variant c.265G>A p.Val89Ile
NC_007530	Chromosome	4509167 C	A	A	A	A	C	A	A	A	C	A	A	A	A	A	IGR	-	-	-
NC_007530	Chromosome	4509169 C	G	G	G	G	C	G	G	G	C	G	G	G	G	G	IGR	-	-	-
NC_007530	Chromosome	4509171 T	G	G	G	G	T	G	G	G	T	G	G	G	G	G	IGR	-	-	-
NC_007530	Chromosome	4514110 T	T	T	T	T	T	C	C	C	C	C	C	C	C	C	GBAA_RS24160	-	molybdopterin molybdenumtransferase MoeA	synonymous_variant c.948A>G p.Gly316Gly
NC_007530	Chromosome	4514967 T	C	C	C	C	C	T	T	T	T	T	T	T	T	T	GBAA_RS24160	-	molybdopterin molybdenumtransferase MoeA	missense_variant c.91A>G p.Ile31Val
NC_007530	Chromosome	4516991 A	G	G	G	G	G	G	G	G	G	G	G	G	G	G	GBAA_RS24175	moaA	GTP 3',8-cyclase MoaA	stop_lost c.700T>C p.Ter234Argext*?
NC_007530	Chromosome	4518916 A	A	A	A	A	A	G	G	G	A	G	G	G	G	A	GBAA_RS24180	-	collagen-like protein	synonymous_variant c.390T>C p.Ser130Ser
NC_007530	Chromosome	4519053 A	G	G	G	G	G	G	G	G	G	G	G	G	G	G	GBAA_RS24180	-	collagen-like protein	missense_variant c.253T>C p.Ser85Pro
NC_007530	Chromosome	4519433 C	A	A	A	A	A	A	A	A	A	A	A	A	A	A	IGR	-	-	-
NC_007530	Chromosome	4522673 A	A	G	G	A	A	G	A	A	A	A	A	G	A	A	GBAA_RS30900	-	spore germination protein	missense_variant c.546_547delAGinsGA p.Asp183Asn
NC_007530	Chromosome	4522674 G	G	A	A	G	G	A	G	G	G	G	G	A	G	G	GBAA_RS30900	-	spore germination protein	missense_variant c.546_547delAGinsGA p.Asp183Asn
NC_007530	Chromosome	4525890 C	C	C	C	C	C	T	C	C	C	C	C	C	C	C	GBAA_RS24220	-	Ger(x)C family spore germination protein	synonymous_variant c.432C>T p.Tyr144Tyr
NC_007530	Chromosome	4529914 G	A	A	A	A	A	G	G	G	G	G	G	G	G	G	GBAA_RS24245	-	leucine--tRNA ligase	synonymous_variant c.975C>T p.Tyr325Tyr
NC_007530	Chromosome	4549765 A	G	G	G	G	G	G	G	G	G	G	G	G	G	G	GBAA_RS24350	-	glycosyltransferase family 1 protein	missense_variant c.622A>G p.Thr208Ala
NC_007530	Chromosome	4550842 A	G	G	G	G	G	G	G	G	G	G	G	G	G	G	GBAA_RS24355	-	molybdenum cofactor guanylyltransferase	synonymous_variant c.330T>C p.Thr110Thr
NC_007530	Chromosome	4552389 T	C	C	C	C	C	T	T	T	T	T	T	T	T	T	GBAA_RS24375	-	S-adenosylmethionine synthase	missense_variant c.1132A>G p.Thr378Ala
NC_007530	Chromosome	4569923 C	C	C	C	C	C	T	T	T	T	T	T	T	T	T	IGR	-	-	-
NC_007530	Chromosome	4570031 C	A	C	A	C	C	C	C	A	C	A	C	C	A	A	IGR	-	-	-
NC_007530	Chromosome	4570263 G	G	G	G	G	G	A	A	A	A	A	A	A	A	A	GBAA_RS24470	-	hypothetical protein	missense_variant c.953C>T p.Ala318Val
NC_007530	Chromosome	4578076 G	A	A	A	A	A	A	A	A	A	A	A	A	A	A	GBAA_RS24525	cydB	cytochrome d ubiquinol oxidase subunit II	missense_variant c.400G>A p.Gly134Ser
NC_007530	Chromosome	4583724 C	T	T	T	T	T	C	C	C	C	C	C	C	C	C	GBAA_RS24555	-	DUF4247 domain-containing protein	synonymous_variant c.324G>A p.Glu108Glu
NC_007530	Chromosome	4587179 A	A	A	A	A	A	C	C	C	C	C	C	C	C	C	GBAA_RS24590	feoB	ferrous iron transport protein B	missense_variant c.1955T>G p.Ile652Ser
NC_007530	Chromosome	4595999 C	C	C	C	C	C	T	C	C	C	C	C	T	C	T	IGR	-	-	-
NC_007530	Chromosome	4596000 A	A	A	A	A	A	T	A	A	A	A	A	T	A	T	IGR	-	-	-
NC_007530	Chromosome	4609358 C	T	T	T	T	T	C	C	C	C	C	C	C	C	C	IGR	-	-	-
NC_007530	Chromosome	4615052 A	A	A	A	A	A	G	G	G	G	G	G	G	G	G	GBAA_RS24735	-	ABC transporter permease	missense_variant c.1010A>G p.Lys337Arg
NC_007530	Chromosome	4618170 A	G	G	G	G	G	G	G	G	G	G	G	G	G	G	GBAA_RS24750	-	GntR family transcriptional regulator	missense_variant c.362T>C p.Leu121Pro
NC_007530	Chromosome	4624259 T	C	C	C	C	C	C	C	C	C	C	C	C	C	C	IGR	-	-	-
NC_007530	Chromosome	4627533 G	A	A	A	A	A	A	A	A	A	A	A	A	A	A	GBAA_RS24800	menC	o-succinylbenzoate synthase	synonymous_variant c.831C>T p.Asn277Asn
NC_007530	Chromosome	4651352 C	T	T	T	T	T	C	C	C	C	C	C	C	C	C	GBAA_RS24960	-	tRNA-Pro	intragenic_variant n.4651352C>T
NC_007530	Chromosome	4651930 C	A	A	A	A	A	A	A	A	A	A	A	A	A	A	GBAA_RS24990	-	tRNA-Thr	intragenic_variant n.4651930C>A
NC_007530	Chromosome	4656143 G	G	G	G	G	G	G	A	A	G	G	A	G	A	A	GBAA_RS25010	-	16S ribosomal RNA	non_coding_transcript_variant
NC_007530	Chromosome	4667166 T	C	C	C	C	C	T	T	T	T	T	T	T	T	T	GBAA_RS25075	-	pyridoxal phosphate-dependent aminotransferase	missense_variant c.901T>C p.Tyr301His
NC_007530	Chromosome	4685383 C	T	T	T	T	T	T	T	T	T	T	T	T	T	T	IGR	-	-	-
NC_007530	Chromosome	4687868 T	C	C	C	C	C	C	C	C	C	C	C	C	C	C	GBAA_RS25185	-	cell surface protein	missense_variant c.5008A>G p.Lys1670Glu
NC_007530	Chromosome	4690591 T	T	T	T	T	T	C	C	C	C	C	C	C	C	C	GBAA_RS25185	-	cell surface protein	missense_variant c.2285A>G p.His762Arg
NC_007530	Chromosome	4707635 C	T	T	T	T	T	C	C	C	C	C	C	C	C	C	GBAA_RS25280	-	DUF4304 domain-containing protein	missense_variant c.353C>T p.Ala118Val
NC_007530	Chromosome	4731977 C	T	T	T	T	T	C	C	C	C	C	C	C	C	C	GBAA_RS25455	sufB	Fe-S cluster assembly protein SufB	missense_variant c.301G>A p.Ala101Thr
NC_007530	Chromosome	4733700 A	G	G	G	G	G	G	G	G	G	G	G	G	G	G	GBAA_RS25465	-	cysteine desulfurase	missense_variant c.268T>C p.Cys90Arg
NC_007530	Chromosome	4739014 C	T	T	T	T	T	T	T	T	T	T	T	T	T	T	GBAA_RS25495	-	methionine import ATP-binding protein MetN 3	synonymous_variant c.684G>A p.Val228Val
NC_007530	Chromosome	4746412 G	G	G	G	G	G	A	A	A	A	A	A	A	A	A	GBAA_RS25565	-	PAP2 family phosphoesterase	missense_variant c.586G>A p.Val196Ile
NC_007530	Chromosome	4761701 T	T	T	T	T	T	C	C	C	C	C	C	C	C	C	GBAA_RS25650	-	methyl-accepting chemotaxis protein	missense_variant c.1238A>G p.Gln413Arg
NC_007530	Chromosome	4770006 G	T	T	T	T	T	G	G	G	G	G	G	G	G	G	GBAA_RS25680	-	hypothetical protein	missense_variant c.333_334delGCinsAA p.Gln112Lys
NC_007530	Chromosome	4770007 C	T	T	T	T	T	C	C	C	C	C	C	C	C	C	GBAA_RS25680	-	hypothetical protein	missense_variant c.333_334delGCinsAA p.Gln112Lys
NC_007530	Chromosome	4779030 G	G	G	G	G	G	A	A	A	A	A	A	A	A	A	IGR	-	-	-
NC_007530	Chromosome	4784151 G	T	T	T	T	T	T	T	T	T	T	T	T	T	T	IGR	-	-	-
NC_007530	Chromosome	4795316 G	A	A	A	A	A	G	G	G	G	G	G	G	G	G	IGR	-	-	-
NC_007530	Chromosome	4795327 T	C	C	C	C	C	C	C	C	C	C	C	C	C	C	IGR	-	-	-
NC_007530	Chromosome	4799250 G	A	A	A	A	A	G	G	G	G	G	G	G	G	G	GBAA_RS25855	-	hypothetical protein	synonymous_variant c.843G>A p.Thr281Thr
NC_007530	Chromosome	4804982 T	T	T	T	T	T	C	C	C	C	C	C	C	C	C	GBAA_RS25875	-	alanine:cation symporter family protein	synonymous_variant c.849A>G p.Ala283Ala
NC_007530	Chromosome	4813403 G	A	A	A	A	A	G	G	G	G	G	G	G	G	G	GBAA_RS25900	-	MFS transporter	missense_variant c.1096C>T p.Leu366Phe
NC_007530	Chromosome	4831487 C	T	T	T	T	T	C	C	C	C	C	C	C	C	C	GBAA_RS25995	-	iron ABC transporter permease	missense_variant c.490G>A p.Val164Ile
NC_007530	Chromosome	4843773 T	A	A	A	A	A	A	A	A	A	A	A	A	A	A	GBAA_RS31050	-	hypothetical protein	missense_variant c.76A>T p.Ile26Leu
NC_007530	Chromosome	4843811 T	T	T	T	T	T	T	T	C	C	C	C	C	C	C	GBAA_RS31050	-	hypothetical protein	missense_variant c.38A>G p.Lys13Arg
NC_007530	Chromosome	4851662 C	T	T	T	T	T	T	T	T	T	T	T	T	T	T	GBAA_RS26125	-	HNH endonuclease	missense_variant c.110C>T p.Ala37Val
NC_007530	Chromosome	4861189 G	A	A	A	A	A	A	A	A	A	A	A	A	A	A	GBAA_RS26165	-	2,3-bisphosphoglycerate-independent phosphoglycerate mutase	synonymous_variant c.177C>T p.Gly59Gly
NC_007530	Chromosome	4861333 G	G	G	G	G	G	A	A	A	A	A	A	A	A	A	GBAA_RS26165	-	2,3-bisphosphoglycerate-independent phosphoglycerate mutase	synonymous_variant c.33C>T p.Asp11Asp
NC_007530	Chromosome	4863704 C	C	C	C	C	C	T	T	T	T	T	T	T	T	T	GBAA_RS26180	gap	aldehyde dehydrogenase	missense_variant c.775G>A p.Ala259Thr
NC_007530	Chromosome	4865603 C	T	T	T	T	T	T	T	T	T	T	T	T	T	T	IGR	-	-	-
NC_007530	Chromosome	4867375 G	A	A	A	A	A	A	A	A	A	A	A	A	A	A	IGR	-	-	-
NC_007530	Chromosome	4873774 G	G	G	G	G	G	A	A	A	A	A	A	A	A	A	GBAA_RS26250	whiA	sporulation regulator WhiA	synonymous_variant c.132C>T p.Ile44Ile
NC_007530	Chromosome	4880766 A	G	G	G	G	G	G	G	G	G	G	G	G	G	G	GBAA_RS26290	-	pyrophosphatase PpaX	missense_variant c.623T>C p.Leu208Pro
NC_007530	Chromosome	4883247 G	A	A	A	A	A	G	G	G	G	G	G	G	G	G	IGR	-	-	-
NC_007530	Chromosome	4885190 T	C	C	C	C	C	C	C	C	C	C	C	C	C	C	GBAA_RS26315	-	excinuclease ABC subunit UvrA	synonymous_variant c.2070A>G p.Gln690Gln
NC_007530	Chromosome	4885496 T	T	T	T	T	T	C	C	C	C	C	C	C	C	C	GBAA_RS26315	-	excinuclease ABC subunit UvrA	synonymous_variant c.1764A>G p.Ser588Ser
NC_007530	Chromosome	4890546 T	C	C	C	C	C	T	T	T	T	T	T	T	T	T	GBAA_RS26325	-	transposase	missense_variant c.14A>G p.Tyr5Cys
NC_007530	Chromosome	4907768 A	A	A	A	A	A	G	A	A	A	A	A	A	A	A	GBAA_RS26430	-	peptide chain release factor 2	synonymous_variant c.450T>C p.Gly150Gly
NC_007530	Chromosome	4920520 T	T	T	T	T	T	T	A	A	A	A	A	A	T	A	GBAA_RS26490	-	UDP-N-acetylglucosamine 2-epimerase (non-hydrolyzing)	synonymous_variant c.231_234delACGAinsGCGT p.79
NC_007530	Chromosome	4920646 C	C	C	C	C	C	T	T	T	T	T	T	T	T	T	GBAA_RS26490	-	UDP-N-acetylglucosamine 2-epimerase (non-hydrolyzing)	synonymous_variant c.108G>A p.Ser36Ser
NC_007530	Chromosome	4929312 T	G	G	G	G	G	G	G	G	G	G	G	G	G	G	GBAA_RS26520	-	hypothetical protein	missense_variant c.1013T>G p.Val338Gly
NC_007530	Chromosome	4931390 C	C	C	C	C	C	C	A	C	C	C	C	C	C	C	GBAA_RS26530	-	6-phospho-beta-glucosidase	synonymous_variant c.210G>T p.Thr70Thr
NC_007530	Chromosome	4936424 A	T	T	T	T	T	A	A	A	A	A	A	A	A	A	GBAA_RS26565	celB	PTS system, cellobiose-specific IIC component	missense_variant c.917T>A p.Val306Asp
NC_007530	Chromosome	4941461 G	A	A	A	A	A	A	A	A	A	A	A	A	A	A	GBAA_RS26590	-	YhgE/Pip domain-containing protein	missense_variant c.1409C>T p.Ala470Val
NC_007530	Chromosome	4945492 A	G	G	G	G	G	G	G	G	G	G	G	G	G	G	GBAA_RS26605	-	transposase	stop_lost&splice_region_variant c.931T>C p.Ter311Glnext*?
NC_007530	Chromosome	4968415 G	A	A	A	A	A	A	A	A	A	A	A	A	A	A	GBAA_RS26695	-	BCCT family transporter	missense_variant c.155C>T p.Ser52Phe
NC_007530	Chromosome	5003077 A	G	G	G	G	G	G	G	G	G	G	G	G	G	G	GBAA_RS26855	-	hypothetical protein	missense_variant c.932T>C p.Val311Ala
NC_007530	Chromosome	5012119 C	A	A	A	A	A	A	A	A	A	A	A	A	A	A	IGR	-	-	-
NC_007530	Chromosome	5015594 T	C	C	C	C	C	C	C	C	C	C	C	C	C	C	GBAA_RS26910	-	M23 family peptidase	synonymous_variant c.147A>G p.Pro49Pro
NC_007530	Chromosome	5020877 A	A	A	A	A	A	G	A	A	A	A	A	A	A	A	GBAA_RS26940	murA	UDP-N-acetylglucosamine 1-carboxyvinyltransferase	synonymous_variant c.420T>C p.Val140Val
NC_007530	Chromosome	5027151 A	A	A	A	A	A	C	C	C	C	C	C	C	C	C	GBAA_RS26965	-	NADH-quinone oxidoreductase subunit L	missense_variant c.418T>G p.Trp140Gly
NC_007530	Chromosome	5030010 C	T	T	T	T	T	C	C	C	C	C	C	C	C	C	GBAA_RS26990	-	NADH-quinone oxidoreductase subunit D	synonymous_variant c.963G>A p.Glu321Glu
NC_007530	Chromosome	5031640 C	C	C	C	C	C	G	G	G	G	G	G	G	G	G	GBAA_RS26995	-	NADH dehydrogenase subunit C	missense_variant c.290_291delCGinsTC p.Ala97Val
NC_007530	Chromosome	5031641 G	G	G	G	G	G	A	A	A	A	A	A	A	A	A	GBAA_RS26995	-	NADH dehydrogenase subunit C	missense_variant c.290_291delCGinsTC p.Ala97Val
NC_007530	Chromosome	5031664 C	C	C	C	C	C	T	T	T	T	T	T	T	T	T	GBAA_RS26995	-	NADH dehydrogenase subunit C	synonymous_variant c.267G>A p.Glu89Glu
NC_007530	Chromosome	5040523 C	T	T	T	T	T	T	T	T	T	T	T	T	T	T	GBAA_RS31095	-	hypothetical protein	missense_variant c.125G>A p.Gly42Glu
NC_007530	Chromosome	5042438 A	A	A	A	A	A	G	G	G	G	G	G	G	G	G	GBAA_RS27045	-	ATP synthase subunit delta	synonymous_variant c.282T>C p.Asn94Asn
NC_007530	Chromosome	5060677 G	G	G	G	G	G	A	A	A	A	A	A	A	A	A	IGR	-	-	-
NC_007530	Chromosome	5061046 T	T	T	T	T	T	G	G	G	G	G	G	G	G	G	GBAA_RS27170	glpX	fructose-bisphosphatase class II	missense_variant c.776A>C p.Asp259Ala
NC_007530	Chromosome	5063334 A	A	A	A	A	A	G	G	G	G	G	G	G	G	G	IGR	-	-	-
NC_007530	Chromosome	5069665 A	A	A	A	A	A	G	G	G	G	G	G	G	G	G	GBAA_RS27210	-	acyl-CoA dehydrogenase	missense_variant c.470T>C p.Ile157Thr
NC_007530	Chromosome	5072166 A	A	A	A	A	A	G	G	G	G	G	G	G	G	G	GBAA_RS27220	-	3-hydroxyacyl-CoA dehydrogenase	missense_variant c.46T>C p.Ser16Pro
NC_007530	Chromosome	5077771 G	A	A	A	A	A	A	A	A	A	A	A	A	A	A	GBAA_RS27240	uvsE	UV damage endonuclease UvsE	missense_variant c.599G>A p.Cys200Tyr
NC_007530	Chromosome	5078148 T	T	T	T	T	T	A	A	A	A	A	A	T	A	A	GBAA_RS27240	uvsE	UV damage endonuclease UvsE	missense_variant c.958_962delGGTAAinsTTACC p.SerSer320LeuPro
NC_007530	Chromosome	5078149 A	A	A	A	A	A	C	C	C	C	C	C	A	C	C	GBAA_RS27240	uvsE	UV damage endonuclease UvsE	missense_variant c.958_962delGGTAAinsTTACC p.SerSer320LeuPro
NC_007530	Chromosome	5078150 A	A	A	A	A	A	C	C	C	C	C	C	A	C	C	GBAA_RS27240	uvsE	UV damage endonuclease UvsE	missense_variant c.958_962delGGTAAinsTTACC p.SerSer320LeuPro
NC_007530	Chromosome	5085543 A	G	G	G	G	G	G	G	G	G	G	G	G	G	G	GBAA_RS27275	-	ABC transporter ATP-binding protein	missense_variant c.605T>C p.Val202Ala
NC_007530	Chromosome	5086144 C	A	A	A	A	A	A	A	A	A	A	A	A	A	A	GBAA_RS27275	-	ABC transporter ATP-binding protein	stop_gained c.4G>T p.Glu2*
NC_007530	Chromosome	5087266 A	G	G	G	G	G	G	G	G	G	G	G	G	G	G	IGR	-	-	-
NC_007530	Chromosome	5119683 A	A	A	A	A	A	T	T	T	T	T	T	T	T	T	IGR	-	-	-
NC_007530	Chromosome	5119709 C	C	C	C	C	C	T	T	T	T	T	T	T	T	T	IGR	-	-	-
NC_007530	Chromosome	5135426 C	T	T	T	T	T	T	T	T	T	T	T	T	T	T	GBAA_RS27525	-	LacI family transcriptional regulator	missense_variant c.496G>A p.Val166Met
NC_007530	Chromosome	5138394 G	A	A	A	A	A	G	G	G	G	G	G	G	G	G	GBAA_RS27540	-	homoserine O-succinyltransferase	missense_variant c.577C>T p.Arg193Cys
NC_007530	Chromosome	5142320 T	T	T	T	T	T	C	C	C	C	C	C	C	C	C	GBAA_RS27555	-	noncanonical pyrimidine nucleotidase, YjjG family	synonymous_variant c.420A>G p.Ser140Ser
NC_007530	Chromosome	5146943 G	A	A	A	A	A	G	G	G	G	G	G	G	G	G	GBAA_RS27580	thiD	hydroxymethylpyrimidine/phosphomethylpyrimidine kinase	missense_variant c.175G>A p.Ala59Thr
NC_007530	Chromosome	5149702 T	T	T	T	T	T	T	T	A	A	A	A	A	A	A	GBAA_RS27595	-	carbon starvation protein A	missense_variant c.1444A>T p.Ile482Phe
NC_007530	Chromosome	5153860 A	G	G	G	G	G	G	G	G	G	G	G	G	G	G	GBAA_RS27610	-	glycosyltransferase	missense_variant c.544T>C p.Tyr182His
NC_007530	Chromosome	5158582 A	G	G	G	G	G	G	G	G	G	G	G	G	G	G	GBAA_RS27630	-	methyl-accepting chemotaxis protein	missense_variant c.550A>G p.Lys184Glu
NC_007530	Chromosome	5164931 T	C	C	C	C	C	C	C	C	C	C	C	C	C	C	GBAA_RS27655	-	ABC transporter ATP-binding protein	missense_variant c.482T>C p.Val161Ala
NC_007530	Chromosome	5188327 C	A	A	A	A	A	A	A	A	A	A	A	A	A	A	GBAA_RS27750	-	transporter	missense_variant c.23G>T p.Arg8Leu
NC_007530	Chromosome	5195815 A	G	G	G	G	G	A	A	A	A	A	A	A	A	A	GBAA_RS27785	guaC	guanosine monophosphate reductase	missense_variant c.935_936delATinsGC p.Asp312Gly
NC_007530	Chromosome	5195816 T	C	C	C	C	C	T	T	T	T	T	T	T	T	T	GBAA_RS27785	guaC	guanosine monophosphate reductase	missense_variant c.935_936delATinsGC p.Asp312Gly
NC_007530	Chromosome	5210933 G	G	G	G	G	G	A	A	A	A	A	A	A	A	A	GBAA_RS27875	-	DHH family phosphoesterase	synonymous_variant c.1689C>T p.Ser563Ser
NC_007530	Chromosome	5213423 C	G	G	G	G	G	C	C	C	C	C	C	C	C	C	GBAA_RS27880	-	DUF2232 domain-containing protein	synonymous_variant c.216G>C p.Pro72Pro

a IGR, Intergenic region
