# Supplementary figures and images for: Loss of Bacitracin Resistance Due to a Large Genomic Deletion among Bacillus anthracis Strains
Source: mSystems. 2018 Oct 30;3(5):e00182-18. doi: 10.1128/mSystems.00182-18 (PMC6208641; doi:10.1128/mSystems.00182-18)

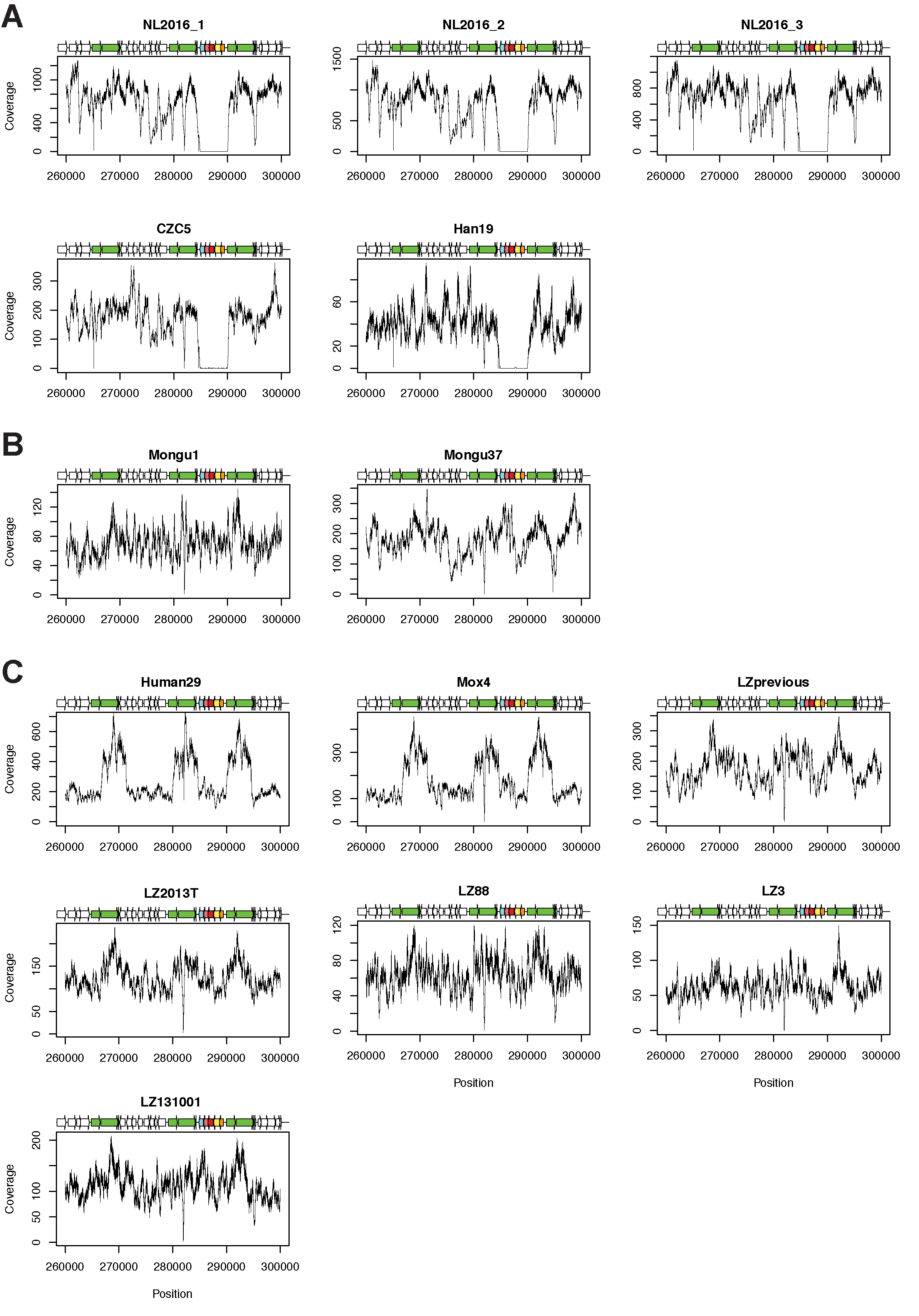

Supplement: FIG S1 [file sys005182281sf1.tif]

**A**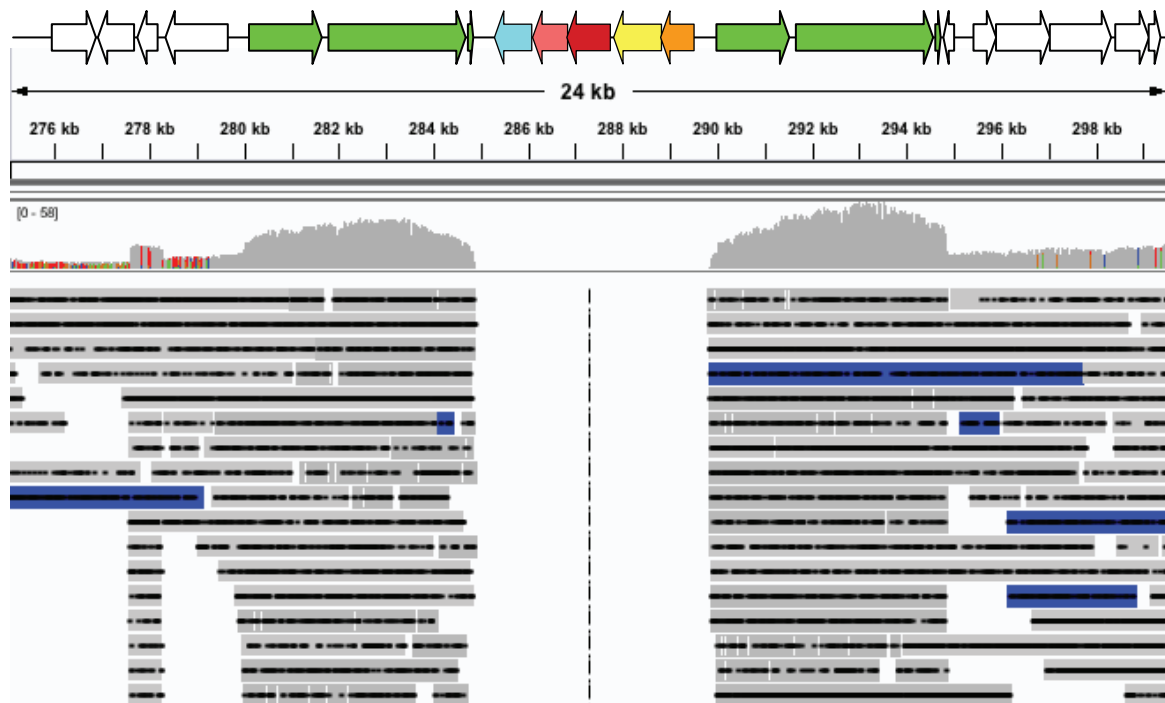**B**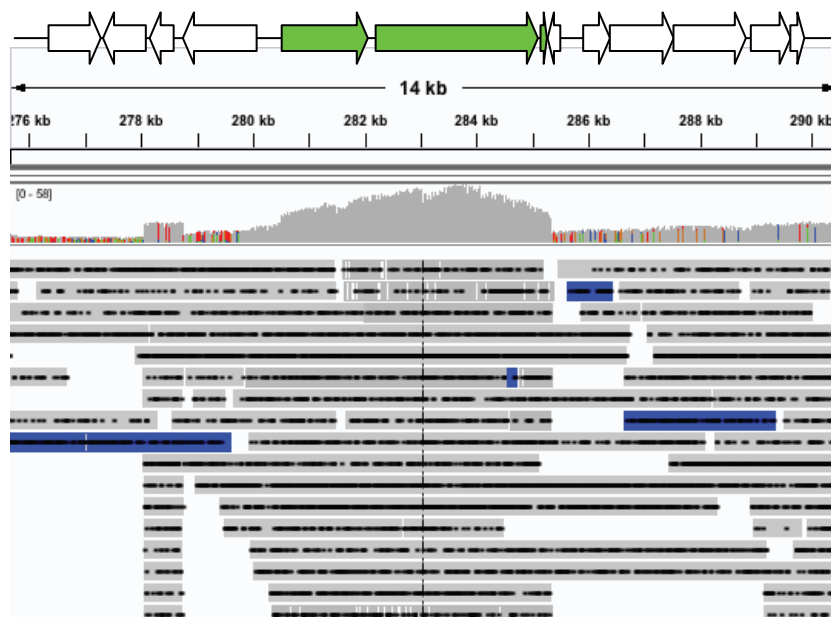

Supplement: FIG S2 [file sys005182281sf2.pdf]

**A**

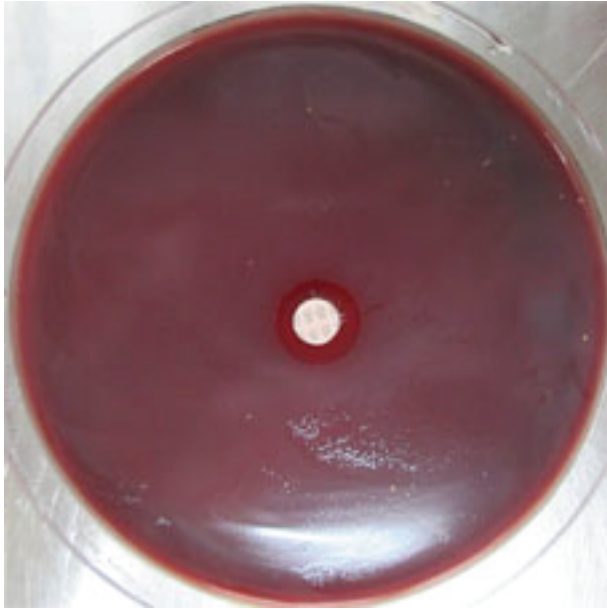

**B**

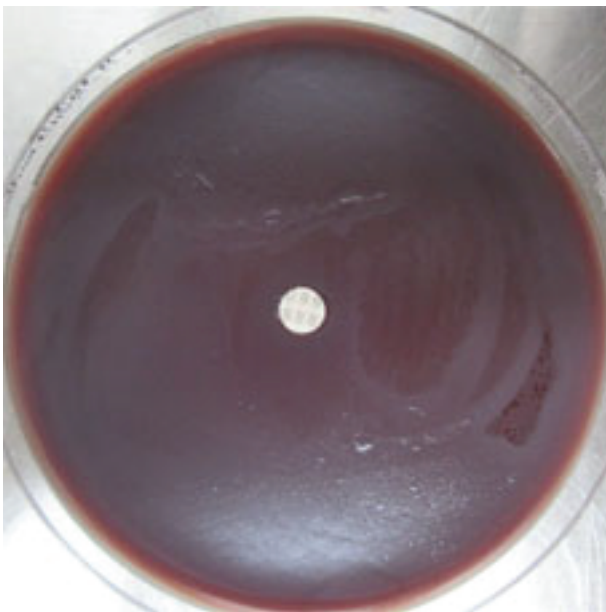

**C**

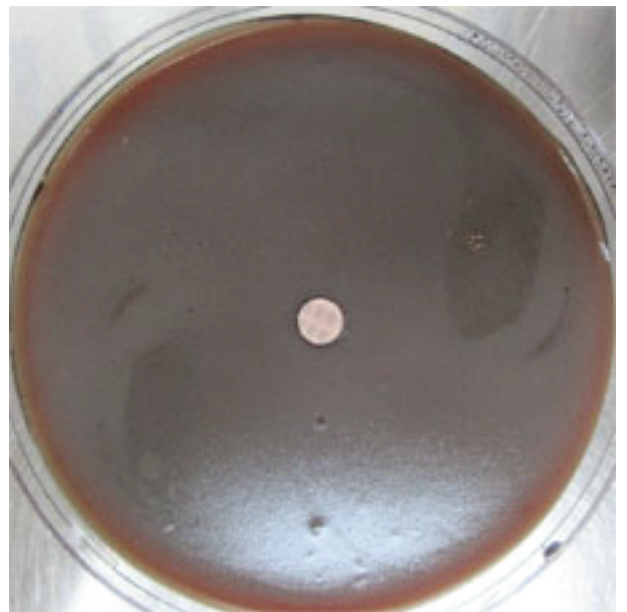

Supplement: FIG S3 [file sys005182281sf3.pdf]

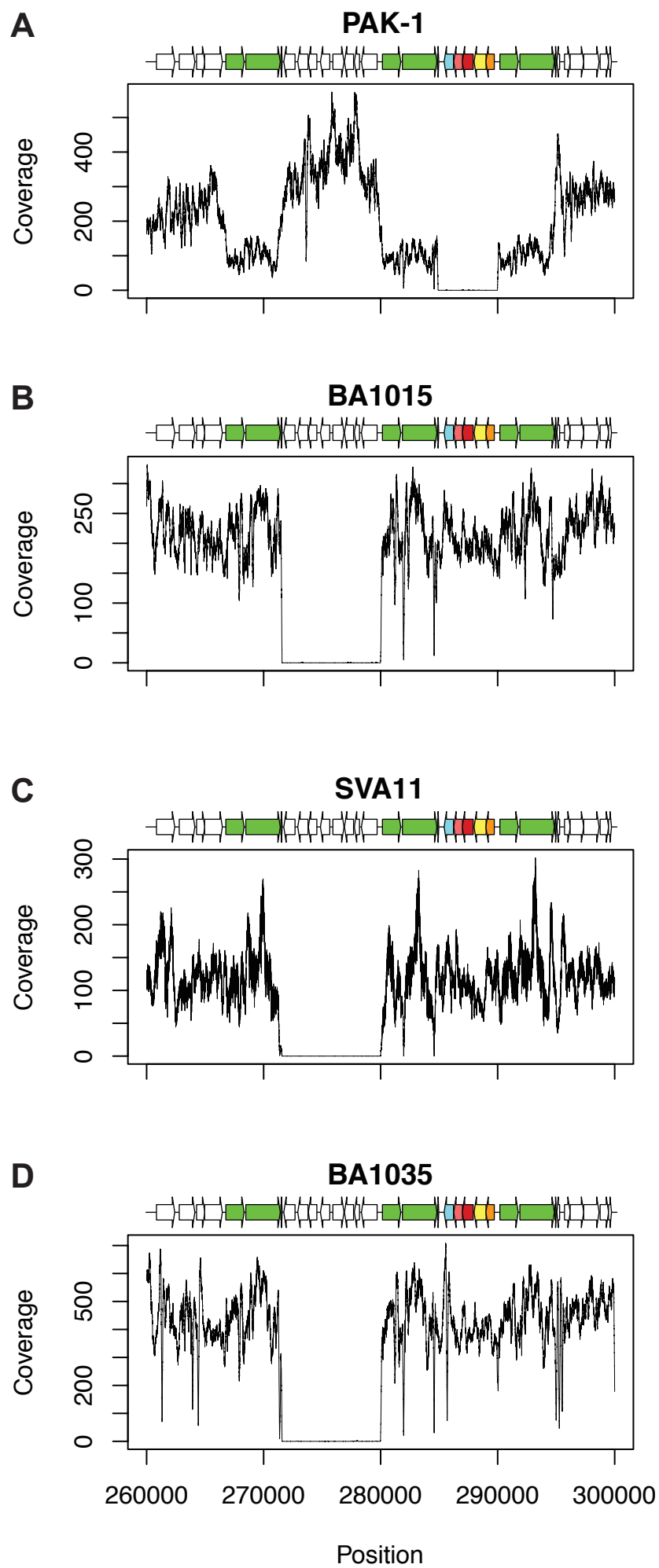

Supplement: FIG S5 [file sys005182281sf5.pdf]

**A**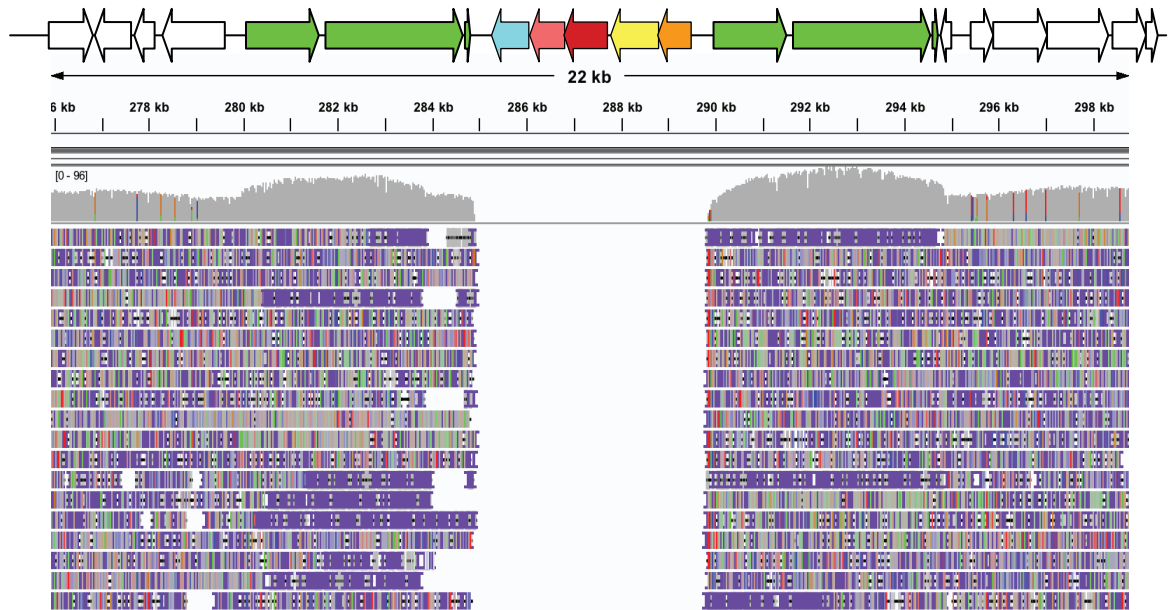**B**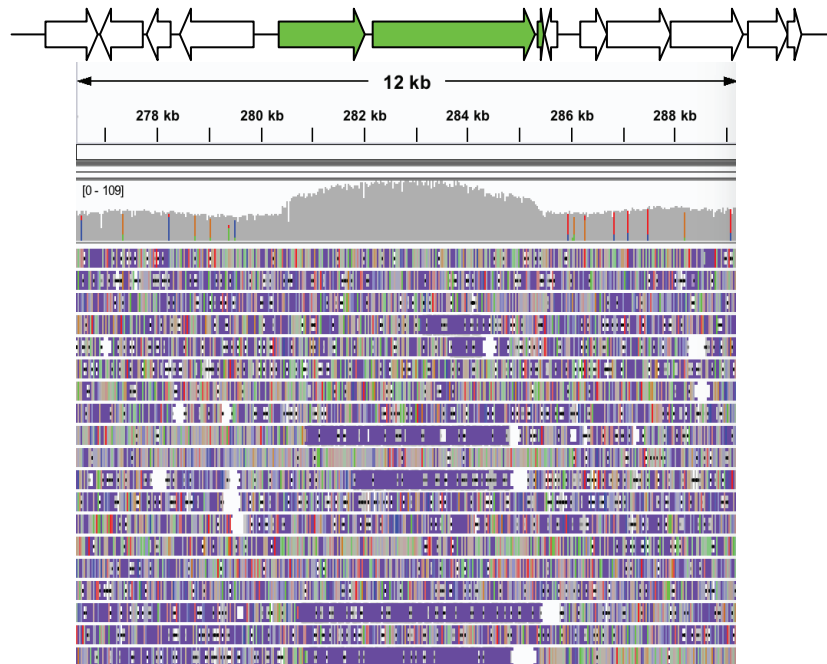

Supplement: FIG S6 [file sys005182281sf6.pdf]
