# Supplementary material for: Loss of Bacitracin Resistance Due to a Large Genomic Deletion among Bacillus anthracis Strains
Source: mSystems. 2018 Oct 30;3(5):e00182-18. doi: 10.1128/mSystems.00182-18 (PMC6208641; doi:10.1128/mSystems.00182-18)
Supplement: TABLE S2 [file sys005182281st2.pdf]

**Table S2.** ***Bacillus anthracis*** **strains whose complete sequences are available at the RefSeq database.**

| Strain | RefSeq Assembly accession | Isolation country | Reference of whole genome sequencing |
|---|---|---|---|
| Ames | GCF_000007845.1 | United States | Read et al., Nature, 2003 |
| Sterne | GCF_000008165.1 | South Africa | N/A |
| Ames Ancestor; A2084 | GCF_000008445.1 | United States | Ravel et al., J Bacteriol, 2008 |
| CDC 684 | GCF_000021445.1 | N/A[a] | N/A |
| A0248 | GCF_000022865.1 | N/A | N/A |
| H9401 | GCF_000258885.1 | South Korea | Chun et al., J Bacteriol, 2012 |
| A16R | GCF_000512775.1 | N/A | N/A |
| A16 | GCF_000512835.1 | N/A | N/A |
| SVA11 | GCF_000583105.1 | Sweden | Agren et al., PLoS One, 2014 |
| HYU01 | GCF_000725325.1 | Sourth Korea | Kim et al., Genome Announc, 2014 |
| 2000031021 | GCF_000742655.1 | United States | Daligault et al., Genome Announc, 2014 |
| Vollum | GCF_000742895.1 | United Kingdom | Daligault et al., Genome Announc, 2014 |
| Cvac02 | GCF_000747335.1 | China | N/A |
| Han | GCF_000747375.1 | China | N/A |
| Ames A0462 | GCF_000830095.1 | United States | N/A |
| PAK-1 | GCF_000832425.1 | Pakistan | Johnson et al., Genome Announc, 2015 |
| Vollum 1B | GCF_000832445.1 | United States | Johnson et al., Genome Announc, 2015 |
| K3 | GCF_000832465.1 | South Africa | Johnson et al., Genome Announc, 2015 |
| Ohio ACB | GCF_000832505.1 | United States | Johnson et al., Genome Announc, 2015 |
| SK-102 | GCF_000832565.1 | United States | Johnson et al., Genome Announc, 2015 |
| Pasteur | GCF_000832585.1 | N/A | Johnson et al., Genome Announc, 2015 |
| Sterne | GCF_000832635.1 | South Africa | Johnson et al., Genome Announc, 2015 |
| BA1015 | GCF_000832665.1 | United States | Johnson et al., Genome Announc, 2015 |
| BA1035 | GCF_000832725.1 | South Africa | Johnson et al., Genome Announc, 2015 |
| RA3 | GCF_000832745.1 | France | Johnson et al., Genome Announc, 2015 |
| V770-NP-1R | GCF_000832785.1 | United States | Johnson et al., Genome Announc, 2015 |
| 2002013094 | GCF_000832965.1 | United States | Johnson et al., Genome Announc, 2015 |
| Ames_BA1004 | GCF_000833065.1 | United States | Johnson et al., Genome Announc, 2015 |
| Canadian_bison | GCF_000833125.1 | Canada | Johnson et al., Genome Announc, 2015 |
| Turkey32 | GCF_000833275.1 | Turkey | Johnson et al., Genome Announc, 2015 |
| A1144 | GCF_000875715.1 | Argentina | Chen et al., J Virol, 2016 |
| Stendal | GCF_001543225.1 | Germany | Antwerpen et al., Genome Announc, 2016 |
| Tangail-1 | GCF_001654475.1 | Bangladesh | N/A |
| Tyrol 4675 | GCF_001936375.1 | Austria | Antwerpen et al., Genome Announc, 2017 |
| SPV842_15 | GCF_001990245.1 | Brazil | Siqueira et al, Braz J Microbiol, 2018 |
| FDAARGOS_341 | GCF_002208785.1 | N/A | N/A |
| 14RA5914 | GCF_002277915.1 | Germany | Elschner et al, Genome Announc, 2017 |
| Shikan-NIID | GCF_002356575.1 | Japan | Okutani et al, Genome Announc, 2015 |
| London_499 | GCF_003227955.1 | United Kingdom | Pullan et al, Microbiol Resour Announc, 2018, in press |

*a* N/A, not available.
