# Supplementary material for: Loss of Bacitracin Resistance Due to a Large Genomic Deletion among Bacillus anthracis Strains
Source: mSystems. 2018 Oct 30;3(5):e00182-18. doi: 10.1128/mSystems.00182-18 (PMC6208641; doi:10.1128/mSystems.00182-18)
Supplement: FIG S4 [file sys005182281sf4.pdf]

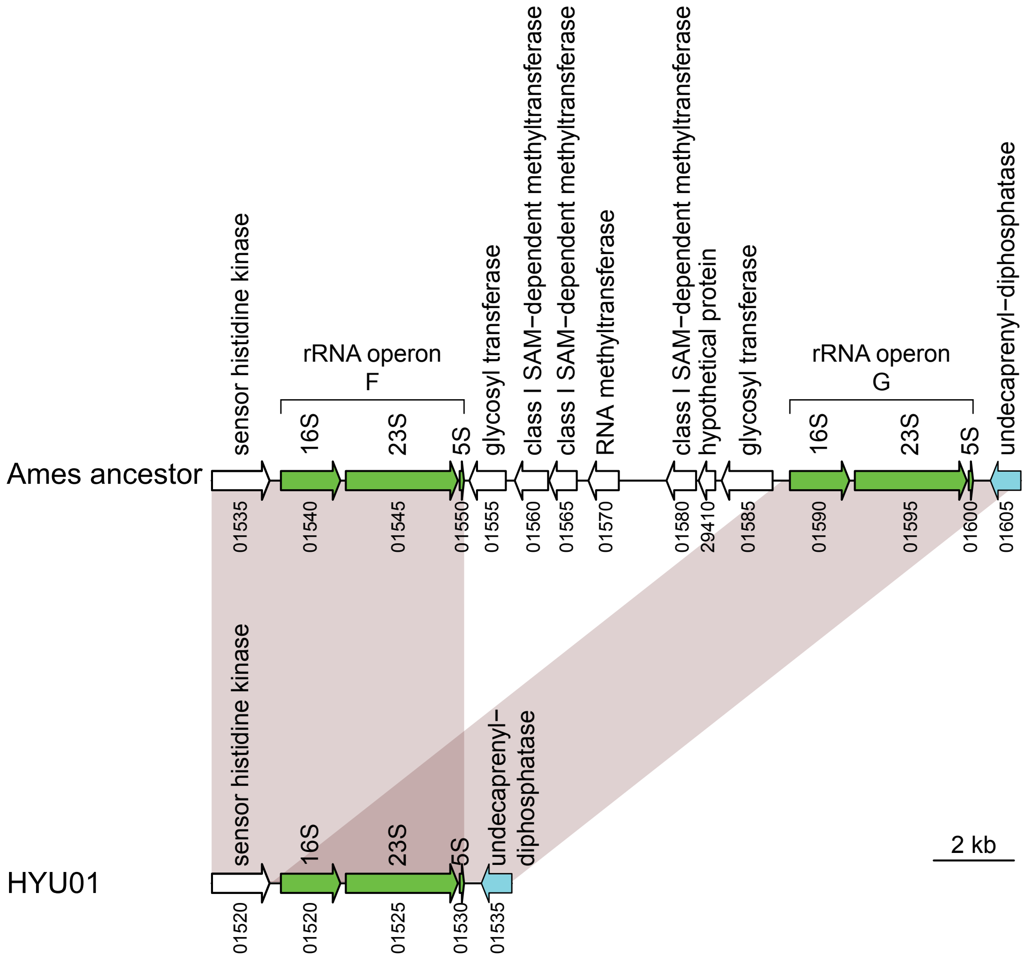

Figure S4. Comparison of genomic regions of deletion FG. Genes are indicated with a number of a locus tag following "GBAA_RS" for Ames ancestor and "HYU01_RS" for HYU01. Colors of arrows are the same as in Fig. 2A.
