# Supplementary material for: Loss of Bacitracin Resistance Due to a Large Genomic Deletion among Bacillus anthracis Strains
Source: mSystems. 2018 Oct 30;3(5):e00182-18. doi: 10.1128/mSystems.00182-18 (PMC6208641; doi:10.1128/mSystems.00182-18)
Supplement: TABLE S3 [file sys005182281st3.pdf]

Table S3. Locus tags of gene homologs within the bacitracin resistance gene cluster.

Species	Strain	Accession#	*bcrC*	*uppP*	*bcrB*	*bcrA*	*bacS*	*bacR*
Bacillus altitudinis	SGAir0031	NZ_CP022319.1	CFN77_RS17470	CFN77_RS15030	-	CFN77_RS19110	-	CFN77_RS10965
Bacillus altitudinis	P-10	NZ_CP024204.1	CSE15_RS17420	CSE15_RS14545	-	CSE15_RS19010	-	CSE15_RS10715
Bacillus amyloliquefaciens	DSM 7	NC_014551.1	BAMF_RS38290	BAMF_RS35385	-	-	-	BAMF_RS31695
Bacillus amyloliquefaciens	TA208	NC_017188.1	BAMTA208_RS17860	BAMTA208_RS14945	-	-	-	BAMTA208_RS05420
Bacillus amyloliquefaciens	LL3	NC_017190.1	LL3_RS18115	LL3_RS15205	-	-	-	LL3_RS11905
Bacillus amyloliquefaciens	XH7	NC_017191.1	BAXH7_RS17890	BAXH7_RS14975	-	-	-	BAXH7_RS05435
Bacillus amyloliquefaciens	IT-45	NC_020272.1	KSO_RS02100	KSO_RS04860	-	-	-	KSO_RS08530
Bacillus amyloliquefaciens	Y2	NC_017912.1	MUS_RS18595	MUS_RS15825	MUS_RS03990	MUS_RS03985	MUS_RS04005	MUS_RS04000
Bacillus amyloliquefaciens	CC178	NC_022653.1	U471_RS16800	U471_RS14075	-	U471_RS17480	-	U471_RS17465
Bacillus amyloliquefaciens	LFB112	NC_023073.1	U722_RS17005	U722_RS14280	-	-	-	U722_RS10855
Bacillus amyloliquefaciens	L-H15	NZ_CP010556.1	SB45_RS16760	SB45_RS14055	-	-	-	SB45_RS10645
Bacillus amyloliquefaciens	KHG19	NZ_CP007242.1	KHU1_RS15935	KHU1_RS13230	-	KHU1_RS17395	-	KHU1_RS09755
Bacillus amyloliquefaciens	L-S60	NZ_CP011278.1	XM40_RS16785	XM40_RS14085	-	-	-	XM40_RS10660
Bacillus amyloliquefaciens	MBE1283	NZ_CP013727.1	AVM03_RS11630	AVM03_RS08940	-	AVM03_RS12995	-	AVM03_RS05255
Bacillus amyloliquefaciens	S499	NZ_CP014700.1	AS588_RS16960	AS588_RS14195	-	-	-	AS588_RS04810
Bacillus amyloliquefaciens	UMAF6639	NZ_CP006058.1	BAMY6639_RS08440	BAMY6639_RS05690	-	BAMY6639_RS09120	-	BAMY6639_RS09105
Bacillus amyloliquefaciens	UMAF6614	NZ_CP006960.1	BAMY6614_RS01300	BAMY6614_RS17595	-	BAMY6614_RS01960	-	BAMY6614_RS01945
Bacillus amyloliquefaciens	B15	NZ_CP014783.1	A1R12_RS17115	A1R12_RS14345	-	A1R12_RS18585	-	A1R12_RS10830
Bacillus amyloliquefaciens	RD7-7	NZ_CP016913.1	BARD7_RS16190	BARD7_RS13535	-	-	-	BARD7_RS10190
Bacillus amyloliquefaciens	Y14	NZ_CP017953.1	BAMY_RS17640	BAMY_RS14930	-	-	-	BAMY_RS11325
Bacillus amyloliquefaciens	LM2303	NZ_CP018152.1	BSF20_RS09170	BSF20_RS11940	-	-	-	BSF20_RS15480
Bacillus amyloliquefaciens	WS-8	NZ_CP018200.1	BSO20_RS14115	BSO20_RS16985	BSO20_RS07905	BSO20_RS07905	BSO20_RS07890	BSO20_RS07890
Bacillus amyloliquefaciens	SRCM101267	NZ_CP021505.1	S101267_RS18875	S101267_RS16120	-	-	-	S101267_RS11910
Bacillus amyloliquefaciens	MT45	NZ_CP011252.1	WV34_RS17700	WV34_RS14720	-	-	-	WV34_RS11330
Bacillus amyloliquefaciens	ALB69	NZ_CP029070.1	DDT09_RS17785	DDT09_RS14960	-	DDT09_RS18450	-	DDT09_RS18435
Bacillus amyloliquefaciens	ALB65	NZ_CP029069.1	BAALB65_RS17920	BAALB65_RS15115	-	BAALB65_RS18580	-	BAALB65_RS18565
Bacillus anthracis	Ames	NC_003997.3	BA_2508	BA_0283	BA_0284	BA_0284	BA_0286	BA_0287
Bacillus anthracis	Sterne	NC_005945.1	BAS2329	BAS0269	BAS0270	BAS0270	BAS0272	BAS0272
Bacillus anthracis	Ames Ancestor; A2084	NC_007530.2	GBAA_RS12400	GBAA_RS01605	GBAA_RS01610	GBAA_RS01610	GBAA_RS01620	GBAA_RS01620
Bacillus anthracis	CDC 684	NC_012581.1	BAMEG_RS10505	BAMEG_RS02085	BAMEG_RS02090	BAMEG_RS02090	BAMEG_RS02100	BAMEG_RS02100
Bacillus anthracis	A0248	NC_012659.1	BAA_RS12910	BAA_RS02100	BAA_RS02105	BAA_RS02105	BAA_RS02115	BAA_RS02115
Bacillus anthracis	H9401	NC_017729.1	H9401_RS12375	H9401_RS07105	-	H9401_RS23155	-	H9401_RS01535
Bacillus anthracis	A16R	NZ_CP001974.2	A16R_RS12915	A16R_RS01615	A16R_RS01620	A16R_RS01620	A16R_RS01630	A16R_RS01535
Bacillus anthracis	A16	NZ_CP001970.2	A16_RS12415	A16_RS01610	A16_RS01615	A16_RS01615	A16_RS01625	A16_RS01625
Bacillus anthracis	SVA11	NZ_CP006742.1	MI63_RS12345	MI63_RS01540	MI63_RS01545	MI63_RS01545	MI63_RS01555	MI63_RS01555
Bacillus anthracis	HYU01	NZ_CP008846.1	HYU01_RS12350	HYU01_RS01535	HYU01_RS01540	HYU01_RS01540	HYU01_RS01550	HYU01_RS01550
Bacillus anthracis	2000031021	NZ_CP007618.1	DJ48_RS09375	DJ48_RS20145	DJ48_RS20140	DJ48_RS20135	DJ48_RS20130	DJ48_RS20125
Bacillus anthracis	Vollum	NZ_CP007666.1	DJ46_RS07750	DJ46_RS24905	DJ46_RS24910	DJ46_RS24910	DJ46_RS24920	DJ46_RS24920
Bacillus anthracis	Ames A0462	NZ_CP010792.1	TD69_RS12395	TD69_RS01595	TD69_RS01600	TD69_RS01600	TD69_RS01610	TD69_RS01610
Bacillus anthracis	PAK-1	NZ_CP009325.1	BD64_RS16305	BD64_RS21580	-	BD64_RS05515	-	BD64_RS27155
Bacillus anthracis	Vollum 1B	NZ_CP009328.1	BF39_RS07310	BF39_RS24455	BF39_RS24460	BF39_RS24460	BF39_RS24470	BF39_RS24470
Bacillus anthracis	K3	NZ_CP009331.1	BF37_RS24690	BF37_RS07530	BF37_RS07525	BF37_RS07520	BF37_RS07515	BF37_RS07510
Bacillus anthracis	Ohio ACB	NZ_CP009341.1	BG02_RS27315	BG02_RS16500	BG02_RS16505	BG02_RS16505	BG02_RS16515	BG02_RS16515
Bacillus anthracis	SK-102	NZ_CP009464.1	BF31_RS22710	BF31_RS05545	BF31_RS05540	BF31_RS05535	BF31_RS05530	BF31_RS05525
Bacillus anthracis	Pasteur	NZ_CP009476.1	BF26_RS02940	BF26_RS13605	BF26_RS13600	BF26_RS13595	BF26_RS13590	BF26_RS13585
Bacillus anthracis	Sterne	NZ_CP009541.1	AW20_RS02415	AW20_RS13220	AW20_RS13215	AW20_RS13210	AW20_RS13205	AW20_RS13200
Bacillus anthracis	BA1015	NZ_CP009544.1	BG01_RS15095	BG01_RS25920	BG01_RS25915	BG01_RS25910	BG01_RS25905	BG01_RS25900
Bacillus anthracis	BA1035	NZ_CP009700.1	BF89_RS01515	BF89_RS12325	BF89_RS12320	BF89_RS12315	BF89_RS12310	BF89_RS12305
Bacillus anthracis	RA3	NZ_CP009697.1	BF90_RS03005	BF90_RS20045	BF90_RS20050	BF90_RS20050	BF90_RS20060	BF90_RS20060
Bacillus anthracis	V770-NP-1R	NZ_CP009598.1	AK39_RS14640	AK39_RS25450	AK39_RS25445	AK39_RS25440	AK39_RS25435	AK39_RS25430
Bacillus anthracis	2002013094	NZ_CP009902.1	BF27_RS26755	BF27_RS09615	BF27_RS09610	BF27_RS09605	BF27_RS09600	BF27_RS09595
Bacillus anthracis	Ames	NZ_CP009981.1	BF25_RS12940	BF25_RS23750	BF25_RS23745	BF25_RS23740	BF25_RS23735	BF25_RS23730
Bacillus anthracis	Canadian_bison	NZ_CP010322.1	BF24_RS01570	BF24_RS18685	BF24_RS18690	BF24_RS18690	BF24_RS18700	BF24_RS18700
Bacillus anthracis	Turkey32	NZ_CP009315.1	AS53_RS22745	AS53_RS12000	AS53_RS12005	AS53_RS12005	AS53_RS12015	AS53_RS12015
Bacillus anthracis	A1144	NZ_CP010852.1	KD35_RS13625	KD35_RS02975	KD35_RS02980	KD35_RS02980	KD35_RS02990	KD35_RS02990
Bacillus anthracis	Stendal	NZ_CP014179.1	AW166_RS13735	AW166_RS02925	AW166_RS02930	AW166_RS02930	AW166_RS02940	AW166_RS02940
Bacillus anthracis	Tangail-1	NZ_CP015779.1	A8C77_RS13740	A8C77_RS02935	A8C77_RS02940	A8C77_RS02940	A8C77_RS02950	A8C77_RS02950
Bacillus anthracis	Parent2	NZ_CP012729.1	AOQ81_RS12410	AOQ81_RS01610	AOQ81_RS01615	AOQ81_RS01615	AOQ81_RS01625	AOQ81_RS01625
Bacillus anthracis	Parent1	NZ_CP012730.1	AOQ80_RS12415	AOQ80_RS01610	AOQ80_RS01615	AOQ80_RS01615	AOQ80_RS01625	AOQ80_RS01625
Bacillus anthracis	PR01	NZ_CP012720.1	AOD59_RS12415	AOD59_RS01610	AOD59_RS01615	AOD59_RS01615	AOD59_RS01625	AOD59_RS01625
Bacillus anthracis	PR02	NZ_CP012721.1	AOD60_RS12415	AOD60_RS01610	AOD60_RS01615	AOD60_RS01615	AOD60_RS01625	AOD60_RS01625
Bacillus anthracis	PR05	NZ_CP012722.1	AOD63_RS12405	AOD63_RS01605	AOD63_RS01610	AOD63_RS01610	AOD63_RS01620	AOD63_RS01620
Bacillus anthracis	PR06	NZ_CP012723.1	AOD64_RS12415	AOD64_RS01610	AOD64_RS01615	AOD64_RS01615	AOD64_RS01625	AOD64_RS01625
Bacillus anthracis	PR07	NZ_CP012724.1	AOD65_RS12410	AOD65_RS01610	AOD65_RS01615	AOD65_RS01615	AOD65_RS01625	AOD65_RS01625
Bacillus anthracis	PR08	NZ_CP012725.1	AOD66_RS12410	AOD66_RS01610	AOD66_RS01615	AOD66_RS01615	AOD66_RS01625	AOD66_RS01625
Bacillus anthracis	PR09-1	NZ_CP012726.1	AOD67_RS12410	AOD67_RS01610	AOD67_RS01615	AOD67_RS01615	AOD67_RS01625	AOD67_RS01625
Bacillus anthracis	PR09-4	NZ_CP012727.1	AOD68_RS12410	AOD68_RS01610	AOD68_RS01615	AOD68_RS01615	AOD68_RS01625	AOD68_RS01625
Bacillus anthracis	PR10-4	NZ_CP012728.1	AOD69_RS12410	AOD69_RS01610	AOD69_RS01615	AOD69_RS01615	AOD69_RS01625	AOD69_RS01625
Bacillus anthracis	Tyrol 4675	NZ_CP018903.1	BVB96_RS12880	BVB96_RS01625	BVB96_RS01630	BVB96_RS01630	BVB96_RS01640	BVB96_RS01640
Bacillus anthracis	SPV842_15	NZ_CP019588.1	BZG08_RS13000	BZG08_RS01630	BZG08_RS01635	BZG08_RS01635	BZG08_RS01645	BZG08_RS01645
Bacillus anthracis	FDAARGOS_341	NZ_CP022044.1	CEQ19_RS28635	CEQ19_RS17335	CEQ19_RS17340	CEQ19_RS17340	CEQ19_RS17350	CEQ19_RS17350
Bacillus anthracis	14RA5914	NZ_CP023001.1	CJF63_RS00575	CJF63_RS18410	CJF63_RS18415	CJF63_RS18415	CJF63_RS18425	CJF63_RS18425
Bacillus anthracis	Shikan-NIID	NZ_AP014833.1	BASH2_RS17595	BASH2_RS28860	BASH2_RS28855	BASH2_RS28850	BASH2_RS28845	BASH2_RS28840
Bacillus anthracis	London_499	NZ_CP029805.1	DNQ11_RS12920	DNQ11_RS01635	DNQ11_RS01640	DNQ11_RS01640	DNQ11_RS01650	DNQ11_RS01650
Bacillus anthracis	Cvac02	NZ_CP008853.1	BACVAC02_RS12430	BACVAC02_RS01600	BACVAC02_RS01605	BACVAC02_RS01605	BACVAC02_RS01615	BACVAC02_RS01615
Bacillus anthracis	Han	NZ_CP008854.1	BAHAN_RS12410	BAHAN_RS01590	BAHAN_RS01595	BAHAN_RS01595	BAHAN_RS01605	BAHAN_RS01605
Bacillus aryabhattai	K13	NZ_CP024035.1	CR091_RS24645	CR091_RS02340	-	CR091_RS10265	CR091_RS12955	CR091_RS21335
Bacillus atrophaeus	SRCM101359	NZ_CP021500.1	S101359_RS18305	S101359_RS15525	-	S101359_RS01505	-	S101359_RS01495
Bacillus atrophaeus	1942	NC_014639.1	BATR1942_RS15510	BATR1942_RS12845	-	BATR1942_RS19245	-	BATR1942_RS19235
Bacillus atrophaeus	NRS 1221A	NZ_CP010778.1	TD68_RS15160	TD68_RS12495	-	TD68_RS18945	-	TD68_RS18935
Bacillus atrophaeus	GQJK17	NZ_CP022653.1	BaGK_RS19090	BaGK_RS16385	-	BaGK_RS01500	-	BaGK_RS01490
Bacillus beveridgei	MLTeJB	NZ_CP012502.1	-	-	-	BBEV_RS08470	-	BBEV_RS06995
Bacillus bombysepticus	Wang	NZ_CP007512.1	-	CY96_RS00670	CY96_RS00675	CY96_RS00675	CY96_RS00685	CY96_RS00685
Bacillus cellulosilyticus	DSM 2522	NC_014829.1	-	-	-	-	-	BCELL_RS09165
Bacillus cereus	ATCC 14579	NC_004722.1	-	BC1384	-	BC4537	-	BC0298
Bacillus cereus	ATCC 10987	NC_003909.8	-	BCE_RS07535	-	BCE_RS22265	-	BCE_RS01655
Bacillus cereus	E33L	NC_006274.1	-	BCE33L_RS01620	BCE33L_RS01625	BCE33L_RS01625	BCE33L_RS01635	BCE33L_RS01635
Bacillus cereus	Q1	NC_011969.1	-	BCQ_RS07365	-	BCQ_RS21970	-	BCQ_RS01525
Bacillus cereus	B4264	NC_011725.1	-	BCB4264_RS01595	BCB4264_RS01600	BCB4264_RS01600	BCB4264_RS01610	BCB4264_RS01610
Bacillus cereus	AH187	NC_011658.1	-	BCAH187_RS07555	-	BCAH187_RS22720	-	BCAH187_RS01635
Bacillus cereus	G9842	NC_011772.1	-	BCG9842_RS01510	BCG9842_RS01515	BCG9842_RS01515	BCG9842_RS01525	BCG9842_RS01525
Bacillus cereus	AH820	NC_011773.1	BCAH820_RS12665	BCAH820_RS01620	BCAH820_RS01625	BCAH820_RS01625	BCAH820_RS01635	BCAH820_RS01635
Bacillus cereus	03BB102	NC_012472.1	-	BCA_RS02510	BCA_RS02515	BCA_RS02515	BCA_RS02525	BCA_RS02525
Bacillus cereus	CI	NC_014335.1	BACI_RS12395	BACI_RS07155	-	BACI_RS22475	-	BACI_RS01675
Bacillus cereus	F837/76	NC_016779.1	BCF_RS12365	BCF_RS06890	-	BCF_RS22300	-	BCF_RS01615
Bacillus cereus	NC7401	NC_016771.1	-	BCN_RS07230	-	BCN_RS22360	-	BCN_RS01575
Bacillus cereus	FRI-35	NC_018491.1	-	BCK_RS01450	-	BCK_RS13205	-	BCK_RS06800
Bacillus cereus	A1	NZ_CP015727.1	-	DA68_RS24880	DA68_RS24885	DA68_RS24885	DA68_RS24895	DA68_RS24895
Bacillus cereus	03BB87	NZ_CP009941.1	-	NT98_RS21905	-	NT98_RS05670	-	NT98_RS27505
Bacillus cereus	D17	NZ_CP009300.1	-	AW22_RS23160	AW22_RS23155	AW22_RS23150	AW22_RS23145	AW22_RS23140
Bacillus cereus	03BB102	NZ_CP009318.1	-	AS54_RS02845	AS54_RS02850	AS54_RS02850	AS54_RS02860	AS54_RS02860
Bacillus cereus	FM1	NZ_CP009369.1	-	BG03_RS20540	-	BG03_RS05005	-	BG03_RS26055
Bacillus cereus	3a	NZ_CP009596.1	BG11_RS08245	BG11_RS19100	BG11_RS19095	BG11_RS19090	BG11_RS19085	BG11_RS19080
Bacillus cereus	G9241	NZ_CP009590.1	-	AQ16_RS05925	-	AQ16_RS17845	-	AQ16_RS11515
Bacillus cereus	ATCC 4342	NZ_CP009628.1	-	BF35_RS06750	-	BF35_RS18380	-	BF35_RS12170
Bacillus cereus	03BB108	NZ_CP009641.1	-	AK40_RS26250	AK40_RS26255	AK40_RS26255	AK40_RS26265	AK40_RS26265
Bacillus cereus	E33L	NZ_CP009968.1	-	BF28_RS29420	BF28_RS29415	BF28_RS29410	BF28_RS29405	BF28_RS29400
Bacillus cereus	S2-8	NZ_CP009605.1	BF33_RS09890	BF33_RS20735	BF33_RS20730	BF33_RS20725	BF33_RS20720	BF33_RS20715
Bacillus cereus	FORC_005	NZ_CP009686.1	-	FORC5_RS01600	FORC5_RS01605	FORC5_RS01605	FORC5_RS01615	FORC5_RS01615
Bacillus cereus	NJ-W	NZ_CP012483.1	-	ACN91_RS27440	ACN91_RS27435	ACN91_RS27430	ACN91_RS27425	ACN91_RS27420
Bacillus cereus	FORC_013	NZ_CP011145.1	-	FORC13_RS01705	FORC13_RS01710	FORC13_RS01710	FORC13_RS01720	FORC13_RS01720

Bacillus cereus	CMCC P0021	NZ_CP011151.1	-	WR47_RS01640	WR47_RS01645	WR47_RS01645	WR47_RS01655	WR47_RS01655
Bacillus cereus	CMCC P0011	NZ_CP011153.1	-	WR51_RS01590	WR51_RS01595	WR51_RS01595	WR51_RS01605	WR51_RS01605
Bacillus cereus	HN001	NZ_CP011155.1	-	WR52_RS01455	WR52_RS01460	WR52_RS01460	WR52_RS01470	WR52_RS01470
Bacillus cereus	FORC_024	NZ_CP012691.1	-	FORC24_RS01620	FORC24_RS01625	FORC24_RS01625	FORC24_RS01635	FORC24_RS01635
Bacillus cereus	AR156	NZ_CP015589.1	-	A7A70_RS16255	-	A7A70_RS04280	-	A7A70_RS10705
Bacillus cereus	ISSFR-9F	NZ_CP018933.1	BEI49_RS12480	BEI49_RS01375	BEI49_RS01380	BEI49_RS01380	BEI49_RS01390	BEI49_RS01390
Bacillus cereus	ISSFR-3F	NZ_CP018931.1	BEI48_RS16075	BEI48_RS27180	BEI48_RS27175	BEI48_RS27170	BEI48_RS27165	BEI48_RS27160
Bacillus cereus	JEM-2	NZ_CP018935.1	BEI53_RS24825	BEI53_RS07610	BEI53_RS07605	BEI53_RS07600	BEI53_RS07595	BEI53_RS07590
Bacillus cereus	FORC021	NZ_CP014486.1	-	FORC21_RS01645	FORC21_RS01650	FORC21_RS01650	FORC21_RS01660	FORC21_RS01660
Bacillus cereus	D12_2	NZ_CP016315.1	-	BA203_RS01625	BA203_RS01630	BA203_RS01630	BA203_RS01640	BA203_RS01640
Bacillus cereus	M3	NZ_CP016316.1	-	BA202_RS01715	BA202_RS01720	BA202_RS01720	BA202_RS01730	BA202_RS01730
Bacillus cereus	FORC_048	NZ_CP017234.1	-	FORC48_RS01640	FORC48_RS01645	FORC48_RS01645	FORC48_RS01655	FORC48_RS01655
Bacillus cereus	K8	NZ_CP016595.1	-	BA204_RS01610	BA204_RS01615	BA204_RS01615	BA204_RS01625	BA204_RS01625
Bacillus cereus	M13	NZ_CP016360.1	-	BA201_RS01625	BA201_RS01630	BA201_RS01630	BA201_RS01640	BA201_RS01640
Bacillus cereus	FORC_047	NZ_CP017060.1	-	FORC47_RS07175	-	FORC47_RS23345	-	FORC47_RS01565
Bacillus cereus	C1L	NZ_CP022445.1	-	CGZ63_RS01630	CGZ63_RS01635	CGZ63_RS01635	CGZ63_RS01645	CGZ63_RS01645
Bacillus cereus	CC-1	NZ_CP023179.1	-	CK938_RS07575	CK938_RS01735	CK938_RS01735	CK938_RS01745	CK938_RS01745
Bacillus cereus	MLY1	NZ_CP024655.1	-	CSW12_RS06490	-	CSW12_RS22285	-	CSW12_RS28870
Bacillus cereus	TG1-6	NZ_CP026678.1	-	C2I25_RS04495	-	C2I25_RS16655	-	C2I25_RS10085
Bacillus cereus	FORC60	NZ_CP020383.1	-	FORC60_RS01640	FORC60_RS01645	FORC60_RS01645	FORC60_RS01655	FORC60_RS01655
Bacillus cereus	FT9	NZ_CP008712.1	-	BCRFT9_RS07285	-	BCRFT9_RS21555	-	BCRFT9_RS23145
Bacillus clausii	KSM-K16	NC_006582.1	-	ABC_RS16875	-	ABC_RS18835	-	ABC_RS06195
Bacillus clausii	ENTPro	NZ_CP012475.1	-	DB29_RS14475	-	DB29_RS03910	-	DB29_RS03880
Bacillus clausii	DSM 8716	NZ_CP019985.1	-	BC8716_RS11560	-	BC8716_RS00295	-	BC8716_RS00255
Bacillus coagulans	ATCC 7050	NZ_CP009709.1	BF29_RS06590	BF29_RS13710	-	BF29_RS07080	-	BF29_RS03235
Bacillus coagulans	36D1	NC_016023.1	BCOA_RS28785	BCOA_RS21735	-	BCOA_RS28290	-	BCOA_RS32245
Bacillus coagulans	2-6	NC_015634.1	BCO26_RS11345	BCO26_RS01740	-	BCO26_RS11740	-	BCO26_RS08160
Bacillus coagulans	HM-08	NZ_CP010525.1	SB48_RS04960	SB48_RS15405	-	SB48_RS09890	-	SB48_RS08240
Bacillus coagulans	S-lac	NZ_CP011939.1	AB434_RS17970	AB434_RS07505	-	AB434_RS13030	-	AB434_RS14670
Bacillus coagulans	BC-HY1	NZ_CP017888.1	BIZ35_RS08900	BIZ35_RS16365	-	BIZ35_RS09435	-	BIZ35_RS05500
Bacillus coagulans	R11	NZ_CP026649.1	C3766_RS12930	C3766_RS02185	-	C3766_RS13430	-	C3766_RS09380
Bacillus coagulans	LA204	NZ_CP025437.1	CYJ15_RS13285	CYJ15_RS02350	-	CYJ15_RS13805	-	CYJ15_RS09470
Bacillus cohnii	DSM 6307	NZ_CP018866.1	-	-	-	BC6307_RS06695	-	BC6307_RS21015
Bacillus cytotoxicus	NVH 391-98	NC_009674.1	BCER98_RS08890	BCER98_RS01590	BCER98_RS01595	BCER98_RS01595	BCER98_RS01605	BCER98_RS01605
Bacillus cytotoxicus	CH_38	NZ_CP024098.1	CG483_RS09745	CG483_RS06645	CG483_RS01770	CG483_RS01770	CG483_RS01780	CG483_RS01780
Bacillus cytotoxicus	CH_25	NZ_CP024101.1	CG482_RS09595	CG482_RS01675	CG482_RS01680	CG482_RS01680	CG482_RS01690	CG482_RS01690
Bacillus cytotoxicus	CH_23	NZ_CP024104.1	CG481_RS09605	CG481_RS01675	CG481_RS01680	CG481_RS01680	CG481_RS01690	CG481_RS01690
Bacillus cytotoxicus	CH_13	NZ_CP024109.1	CG479_RS09350	CG479_RS01645	CG479_RS01650	CG479_RS01650	CG479_RS01660	CG479_RS01660
Bacillus cytotoxicus	CH_4	NZ_CP024111.1	CG478_RS05530	CG478_RS08630	CG478_RS01765	CG478_RS01765	CG478_RS01775	CG478_RS01775
Bacillus cytotoxicus	CH_39	NZ_CP024096.1	CG475_RS09745	CG475_RS06645	CG475_RS01765	CG475_RS01765	CG475_RS01775	CG475_RS01775
Bacillus cytotoxicus	CH_15	NZ_CP024107.1	CG480_RS05530	CG480_RS08630	CG480_RS01765	CG480_RS01765	CG480_RS01775	CG480_RS01775
Bacillus cytotoxicus	CH_2	NZ_CP024116.1	CG476_RS09735	CG476_RS06635	CG476_RS01765	CG476_RS01765	CG476_RS01775	CG476_RS01775
Bacillus cytotoxicus	CH_1	NZ_CP024120.1	CG474_RS09665	CG474_RS01735	CG474_RS01740	CG474_RS01740	CG474_RS01750	CG474_RS01750
Bacillus cytotoxicus	CH_3	NZ_CP024113.1	CG477_RS09700	CG477_RS06600	CG477_RS01765	CG477_RS01765	CG477_RS01775	CG477_RS01775
Bacillus endophyticus	Hbe603	NZ_CP011974.1	-	BEH_RS02485	BEH_RS14905	BEH_RS14900	BEH_RS14895	BEH_RS14890
Bacillus flexus	KLBMP 4941	NZ_CP016790.1	-	BC359_RS18175	-	BC359_RS12420	-	BC359_RS05940
Bacillus gibsonii	FJAT-10019	NZ_CP017070.1	BGM20_RS12460	BGM20_RS09660	-	BGM20_RS16325	-	BGM20_RS16315
Bacillus glycinifermentans	BGLY	NZ_LT603683.1	BGLY_RS21255	BGLY_RS18320	BGLY_RS13925	BGLY_RS13925	BGLY_RS13935	BGLY_RS13940
Bacillus glycinifermentans	KBN06P03352	NZ_CP023481.1	COP00_RS13585	COP00_RS10225	-	COP00_RS14950	-	COP00_RS15725
Bacillus halodurans	C-125	NC_002570.2	-	-	-	AYT26_RS04340	-	AYT26_RS04350
Bacillus horikoshii	20a	NZ_CP020880.1	-	B4U37_RS03300	-	B4U37_RS08545	-	B4U37_RS14050
Bacillus infantis	NRRL B-14911	NC_022524.1	-	N288_RS01095	-	N288_RS03910	-	N288_RS04570
Bacillus intestinalis	T30	NZ_CP011051.1	BIS30_RS07950	BIS30_RS05095	-	BIS30_RS11820	-	BIS30_RS11810
Bacillus kochii	BDGP4	NZ_CP022983.1	-	-	-	CKF48_RS09115	-	CKF48_RS09435
Bacillus krulwichiae	AM31D	NZ_CP020814.1	-	-	-	-	-	BkAM31D_RS07520
Bacillus lehensis	G1	NZ_CP003923.1	-	-	-	BLEG1_RS08510	-	BLEG1_RS10015
Bacillus lentus	NCTC4824	NZ_LS483476.1	-	DQN84_RS02180	-	DQN84_RS19190	-	DQN84_RS02715
Bacillus licheniformis	ATCC 14580; DSM 13	NC_006270.3	TRNA_RS40575	TRNA_RS37750	-	TRNA_RS22955	-	TRNA_RS33680
Bacillus licheniformis	DSM 13	NC_006322.1	BLI_RS19070	BLI_RS16250	-	BLI_RS01440	-	BLI_RS12175
Bacillus licheniformis	HRBL-15TDI7	NZ_CP014781.1	AB684_RS19360	AB684_RS16825	-	AB684_RS01490	-	AB684_RS01480
Bacillus licheniformis	BL1202	NZ_CP017247.1	BL1202_RS20435	BL1202_RS17490	-	BL1202_RS01530	-	BL1202_RS01520
Bacillus licheniformis	SCK B11	NZ_CP014795.1	BaDB11_RS10220	BaDB11_RS07360	-	BaDB11_RS14095	-	BaDB11_RS03255
Bacillus licheniformis	SCDB 14	NZ_CP014842.1	B14_RS02960	B14_RS05840	-	B14_RS20315	-	B14_RS20325
Bacillus licheniformis	SCDB 34	NZ_CP014793.1	B34_RS12415	B34_RS09520	-	B34_RS16360	-	B34_RS16350
Bacillus licheniformis	SCCB 37	NZ_CP014794.1	B37_RS17210	B37_RS14355	-	B37_RS21085	-	B37_RS21075
Bacillus licheniformis	SRCM101441	NZ_CP021507.1	S101441_RS19825	S101441_RS16390	-	S101441_RS01545	-	S101441_RS01535
Bacillus licheniformis	SRCM100141	NZ_CP021669.1	S100141_RS16600	S100141_RS13735	-	S100141_RS20495	-	S100141_RS20485
Bacillus licheniformis	SRCM100027	NZ_CP021677.1	S100027_RS20115	S100027_RS17250	-	S100027_RS01500	-	S100027_RS01490
Bacillus licheniformis	BL-010	NZ_CP022477.1	CG450_RS02410	CG450_RS04990	-	CG450_RS20940	-	CG450_RS20950
Bacillus licheniformis	ATCC 9789	NZ_CP023729.1	CPQ91_RS19665	CPQ91_RS16785	-	CPQ91_RS01490	-	CPQ91_RS12300
Bacillus licheniformis	14ADL4	NZ_CP026673.1	BL14DL4_RS10625	BL14DL4_RS07515	-	BL14DL4_RS14525	-	BL14DL4_RS03365
Bacillus licheniformis	CBA7132	NZ_CP021970.1	CD200_RS19410	CD200_RS16545	-	CD200_RS01535	-	CD200_RS01525
Bacillus megaterium	ATCC 14581	NZ_CP009920.1	-	BG04_RS13765	-	BG04_RS21900	-	BG04_RS11245
Bacillus megaterium	DSM 319	NC_014103.1	-	BMD_RS02245	-	-	-	BMD_RS25415
Bacillus megaterium	QM B1551	NC_014019.1	-	BMQ_RS02240	-	-	-	BMQ_RS25455
Bacillus megaterium	WSH-002	NC_017138.1	-	BMWSH_RS22855	-	BMWSH_RS07770	BMWSH_RS12680	BMWSH_RS04510
Bacillus megaterium	Q3	NZ_CP010586.1	AS52_RS24565	AS52_RS02205	-	-	-	AS52_RS21330
Bacillus megaterium	JX285	NZ_CP018874.1	-	BUW91_RS02275	-	BUW91_RS10425	-	BUW91_RS25975
Bacillus megaterium	YC4-R4	NZ_CP026736.1	C2I28_RS06710	C2I28_RS10410	-	C2I28_RS18480	C2I28_RS21155	C2I28_RS03410
Bacillus methanolicus	MGA3	NZ_CP007739.1	-	BMMGA3_RS01820	-	BMMGA3_RS02890	-	BMMGA3_RS10535
Bacillus muralis	G25-68	NZ_CP017080.1	ABE28_RS05280	ABE28_RS21280	-	-	-	ABE28_RS08600
Bacillus mycoides	ATCC 6462	NZ_CP009692.1	-	BG05_RS02865	BG05_RS02860	BG05_RS02855	BG05_RS02850	BG05_RS02845
Bacillus mycoides	KBAB4	NC_010184.1	-	BCERKBAB4_RS01605	BCERKBAB4_RS01610	BCERKBAB4_RS01610	BCERKBAB4_RS01620	BCERKBAB4_RS01620
Bacillus mycoides	219298	NZ_CP007626.1	-	MA06_RS16405	MA06_RS16415	MA06_RS16415	MA06_RS16425	MA06_RS16425
Bacillus mycoides	WSBC 10204	NZ_CP009746.1	-	BWEI_RS17080	-	BWEI_RS01755	-	BWEI_RS27440
Bacillus mycoides	BTZ	NZ_CP009651.1	-	BG07_RS12465	BG07_RS12455	BG07_RS12450	BG07_RS12445	BG07_RS12440
Bacillus mycoides	Gnyt1	NZ_CP020743.1	B7492_RS15745	B7492_RS01635	B7492_RS01640	B7492_RS01640	B7492_RS01650	B7492_RS01650
Bacillus oceanisediminis	2691	NZ_CP015506.1	-	A361_RS13475	-	A361_RS15260	-	A361_RS15270
Bacillus paralicheniformis	ATCC 9945a	NC_021362.1	BALI_RS19170	BALI_RS16550	BALI_RS12760	BALI_RS12760	BALI_RS12770	BALI_RS12770
Bacillus paralicheniformis	BL-09	NZ_CP010524.1	SC10_RS19370	SC10_RS16750	SC10_RS12975	SC10_RS12975	SC10_RS12985	SC10_RS12985
Bacillus paralicheniformis	MDJK30	NZ_CP020352.1	BLMD_RS19445	BLMD_RS16765	BLMD_RS12940	BLMD_RS12940	BLMD_RS12950	BLMD_RS12950
Bacillus paralicheniformis	14DA11	NZ_CP023168.1	CK945_RS20985	CK945_RS18200	CK945_RS13995	CK945_RS13995	CK945_RS14005	CK945_RS14005
Bacillus paralicheniformis	Bac84	NZ_CP023665.1	CP943_RS20005	CP943_RS17065	CP943_RS13155	CP943_RS13155	CP943_RS13165	CP943_RS13165
Bacillus paralicheniformis	Bac48	NZ_CP023666.1	CP942_RS14255	CP942_RS17220	CP942_RS21155	CP942_RS21155	CP942_RS21150	CP942_RS21145
Bacillus pseudofirmus	OF4	NC_013791.2	-	-	-	-	-	BPOF4_RS07145
Bacillus pumilus	SH-B9	NZ_CP011007.1	UP12_RS17015	UP12_RS14680	-	UP12_RS18540	-	UP12_RS10400
Bacillus pumilus	SAFR-032	NC_009848.4	BPUM_RS16350	BPUM_RS13945	-	BPUM_RS17930	-	BPUM_RS10250
Bacillus pumilus	MTCC B6033	NZ_CP007436.1	BW16_RS16870	BW16_RS14475	-	BW16_RS18490	-	BW16_RS10690
Bacillus pumilus	W3	NZ_CP011150.1	VT48_RS16815	VT48_RS14460	-	-	-	VT48_RS10660
Bacillus pumilus	GR-8	NZ_CP009108.1	ID12_RS02675	ID12_RS00280	-	ID12_RS04270	-	ID12_RS15170
Bacillus pumilus	NJ-M2	NZ_CP012329.1	AKO65_RS06300	AKO65_RS08960	-	AKO65_RS04770	-	AKO65_RS12670
Bacillus pumilus	NJ-V2	NZ_CP012482.1	AMR71_RS02615	AMR71_RS05270	-	AMR71_RS01080	-	AMR71_RS08980
Bacillus pumilus	TUAT1	NZ_AP014928.1	BTUAT1_RS16800	BTUAT1_RS14420	-	BTUAT1_RS18365	-	BTUAT1_RS10405
Bacillus pumilus	SH-B11	NZ_CP010997.1	UP15_RS17155	UP15_RS14820	-	UP15_RS18850	-	UP15_RS10685
Bacillus pumilus	NJ-V	NZ_CP012330.1	AKO66_RS02625	AKO66_RS05285	-	AKO66_RS01090	-	AKO66_RS09030
Bacillus pumilus	PDSLzg-1	NZ_CP016784.1	BEN31_RS17785	BEN31_RS02045	-	BEN31_RS16360	-	BEN31_RS05715
Bacillus pumilus	GLB197	NZ_CP018574.1	BS467_RS06615	BS467_RS09495	-	BS467_RS05035	-	BS467_RS13375
Bacillus pumilus	NCTC10337	NZ_LT906438.1	CKW02_RS17845	CKW02_RS15465	-	CKW02_RS19465	-	CKW02_RS10585
Bacillus safensis	KCTC 12796BP	NZ_CP018197.1	BSL056_RS18145	BSL056_RS15720	-	-	-	BSL056_RS11385
Bacillus safensis	U14-5	NZ_CP015607.1	BSA145_RS03205	BSA145_RS06135	-	-	-	BSA145_RS19565
Bacillus safensis	U41	NZ_CP015610.1	BSA41_RS15315	BSA41_RS12900	-	-	-	BSA41_RS09065
Bacillus safensis	U17-1	NZ_CP015611.1	BSA171_RS02750	BSA171_RS05165	-	-	-	BSA171_RS09005
Bacillus safensis	BRM1	NZ_CP018100.1	BRL64_RS17315	BRL64_RS14700	-	BRL64_RS18820	-	BRL64_RS10945

Bacillus siamensis	SCSIO 05746	NZ_CP025001.1	CWD84_RS02240	CWD84_RS05250	-	-	-	CWD84_RS19140
Bacillus simplex	SH-B26	NZ_CP011008.1	-	UP17_RS22750	-	-	-	UP17_RS15635
Bacillus simplex	DSM 1321	NZ_CP017704.1	-	BS1321_RS14775	-	BS1321_RS24660	-	BS1321_RS22345
Bacillus smithii	DSM 4216	NZ_CP012024.1	BSM4216_RS02315	BSM4216_RS05485	-	BSM4216_RS14770	-	BSM4216_RS15655
Bacillus sonorensis	SRCM101395	NZ_CP021920.1	S101395_RS02545	S101395_RS19935	S101395_RS10175	S101395_RS10170	S101395_RS10165	S101395_RS10160
Bacillus subtilis	168	NC_000964.3	BSU36530	BSU31150	-	BSU02570	-	BSU02550
Bacillus subtilis	W23	NC_014479.1	BSUW23_RS18280	BSUW23_RS15425	-	BSUW23_RS01475	-	BSUW23_RS01465
Bacillus subtilis	BSn5	NC_014976.1	BSN5_RS09265	BSN5_RS06515	-	BSN5_RS13110	-	BSN5_RS13100
Bacillus subtilis	BEST195	NC_017196.2	BSNT_RS19435	BSNT_RS15930	-	BSNT_RS01520	-	BSNT_RS01510
Bacillus subtilis	TU-B-10	NC_016047.1	GYO_RS40705	GYO_RS37755	-	GYO_RS23830	-	GYO_RS23820
Bacillus subtilis	RO-NN-1	NC_017195.1	I33_RS17620	I33_RS14870	-	-	-	I33_RS11085
Bacillus subtilis	QB928	NC_018520.1	B657_RS19330	B657_RS16530	-	B657_RS01570	-	B657_RS01560
Bacillus subtilis	BSP1	NC_019896.1	A7A1_RS02390	A7A1_RS05225	-	A7A1_RS19280	-	A7A1_RS19290
Bacillus subtilis	XF-1	NC_020244.1	C663_RS17870	C663_RS15075	-	C663_RS01490	-	C663_RS01480
Bacillus subtilis	6051-HGW	NC_020507.1	BSU6051_RS18875	BSU6051_RS16145	-	BSU6051_RS01520	-	BSU6051_RS01510
Bacillus subtilis	BAB-1	NC_020832.1	I653_RS17640	I653_RS14845	-	I653_RS01455	-	I653_RS01445
Bacillus subtilis	PY79	NC_022898.1	U712_RS17815	U712_RS15085	-	U712_RS01520	-	U712_RS01510
Bacillus subtilis	BEST7003	NZ_AP012496.1	BEST7003_RS17890	BEST7003_RS15170	-	BEST7003_RS01520	-	BEST7003_RS01510
Bacillus subtilis	JH642 substr. AG174	NZ_CP007800.1	BSUA_RS19700	BSUA_RS16900	-	BSUA_RS01550	-	BSUA_RS01540
Bacillus subtilis	AG1839	NZ_CP008698.1	BSUB_RS19720	BSUB_RS16920	-	BSUB_RS01550	-	BSUB_RS01540
Bacillus subtilis	OH 131.1	NZ_CP007409.1	Q433_RS18505	Q433_RS15685	-	Q433_RS01470	-	Q433_RS01460
Bacillus subtilis	TO-A	NZ_CP005997.1	M036_RS18720	M036_RS15925	-	M036_RS01430	-	M036_RS01420
Bacillus subtilis	ATCC 13952	NZ_CP009748.1	KS08_RS17850	KS08_RS14940	-	-	-	KS08_RS11410
Bacillus subtilis	ATCC 19217	NZ_CP009749.1	KS07_RS16680	KS07_RS13995	KS07_RS03865	KS07_RS03860	KS07_RS03880	KS07_RS03875
Bacillus subtilis	Bs-916	NZ_CP009611.1	KO64_RS17080	KO64_RS14275	-	-	-	KO64_RS10540
Bacillus subtilis	SG6	NZ_CP009796.1	OB04_RS18645	OB04_RS15910	-	OB04_RS01345	-	OB04_RS01335
Bacillus subtilis	168	NZ_CP010052.1	QU35_RS18900	QU35_RS16170	-	QU35_RS01520	-	QU35_RS01510
Bacillus subtilis	PS832	NZ_CP010053.1	QX56_RS18875	QX56_RS16145	-	QX56_RS01520	-	QX56_RS01510
Bacillus subtilis	3NA	NZ_CP010314.1	RP72_RS19650	RP72_RS16850	-	RP72_RS01550	-	RP72_RS01540
Bacillus subtilis	BS49	NZ_LN649259.1	VV34_RS19930	VV34_RS17045	-	VV34_RS01550	-	VV34_RS0154
Bacillus subtilis	KCTC 1028	NZ_CP011115.1	O7A_RS19755	O7A_RS16960	-	O7A_RS01550	-	O7A_RS01540
Bacillus subtilis	HJ5	NZ_CP007173.1	AW03_RS18150	AW03_RS15280	-	AW03_RS01390	-	AW03_RS01380
Bacillus subtilis	UD1022	NZ_CP011534.1	ABA10_RS18285	ABA10_RS15430	-	ABA10_RS01495	-	ABA10_RS01485
Bacillus subtilis	TO-A JPC	NZ_CP011882.1	ABU16_RS02475	ABU16_RS20885	-	ABU16_RS06385	-	ABU16_RS06375
Bacillus subtilis	BSD-2	NZ_CP013654.1	AT706_RS00315	AT706_RS03185	-	AT706_RS17055	-	AT706_RS17065
Bacillus subtilis	DE111	NZ_CP013984.1	AN935_RS18415	AN935_RS15605	-	AN935_RS01310	-	AN935_RS01300
Bacillus subtilis	CU1050	NZ_CP014166.1	AWM80_RS18630	AWM80_RS15830	-	AWM80_RS01555	-	AWM80_RS01545
Bacillus subtilis	CGMCC 2108	NZ_CP014471.1	AWV81_RS19500	AWV81_RS16000	-	AWV81_RS01515	-	AWV81_RS01505
Bacillus subtilis	ATCC 49760	NZ_CP014840.1	A1D11_RS01565	A1D11_RS04235	-	A1D11_RS18270	-	A1D11_RS18280
Bacillus subtilis	SZMC 6179J	NZ_CP015004.1	A3772_RS19635	A3772_RS16835	-	A3772_RS01555	-	A3772_RS01545
Bacillus subtilis	delta6	NZ_CP015975.1	A8O17_RS17885	A8O17_RS15085	-	A8O17_RS01460	-	A8O17_RS01450
Bacillus subtilis	KCTC 3135	NZ_CP015375.1	AS891_RS04465	AS891_RS07270	-	AS891_RS00495	-	AS891_RS00505
Bacillus subtilis	168G	NZ_CP016852.1	BFI33_RS19755	BFI33_RS16955	-	BFI33_RS01555	-	BFI33_RS01545
Bacillus subtilis	HJ0-6	NZ_CP016894.1	BSHJ0_RS19745	BSHJ0_RS16920	-	BSHJ0_RS01540	-	BSHJ0_RS01530
Bacillus subtilis	BS16045	NZ_CP017112.1	BS16045_RS19375	BS16045_RS16575	-	BS16045_RS01555	-	BS16045_RS01545
Bacillus subtilis	BS38	NZ_CP017314.1	BSBS38_RS18770	BSBS38_RS15650	-	BSBS38_RS01520	-	BSBS38_RS01510
Bacillus subtilis	HRBS-10TDI13	NZ_CP015222.1	A4A60_RS19775	A4A60_RS16370	-	A4A60_RS01535	-	A4A60_RS01525
Bacillus subtilis	VV2	NZ_CP017676.1	BKN48_RS07640	BKN48_RS04895	-	BKN48_RS11570	-	BKN48_RS11560
Bacillus subtilis	J-5	NZ_CP018295.1	BHE96_RS18415	BHE96_RS15590	-	BHE96_RS19105	-	BHE96_RS19090
Bacillus subtilis	MJ01	NZ_CP018173.1	BAX60_RS03160	BAX60_RS00175	-	BAX60_RS07090	-	BAX60_RS07080
Bacillus subtilis	KH2	NZ_CP018184.1	BSR08_RS15660	BSR08_RS12115	-	BSR08_RS19695	-	BSR08_RS19685
Bacillus subtilis	29R7-12	NZ_CP017763.1	BKP58_RS14855	BKP58_RS18410	-	BKP58_RS10815	-	BKP58_RS10825
Bacillus subtilis	NCIB 3610	NZ_CP020102.1	B4U62_RS19680	B4U62_RS16845	-	B4U62_RS01550	-	B4U62_RS01540
Bacillus subtilis	GQJK2	NZ_CP020367.1	BSK2_RS18625	BSK2_RS15725	-	BSK2_RS01505	-	BSK2_RS01495
Bacillus subtilis	Bs-115	NZ_CP020722.1	B7470_RS09120	B7470_RS05585	-	B7470_RS13140	-	B7470_RS13130
Bacillus subtilis	TLO3	NZ_CP021169.1	B9N48_RS18255	B9N48_RS15560	-	B9N48_RS01500	-	B9N48_RS01490
Bacillus subtilis	CW14	NZ_CP016767.1	BCV50_RS15420	BCV50_RS18270	-	BCV50_RS11365	-	BCV50_RS11375
Bacillus subtilis	SRCM101444	NZ_CP021498.1	S101444_RS18725	S101444_RS15835	-	S101444_RS01535	-	S101444_RS01525
Bacillus subtilis	SRCM100757	NZ_CP021499.1	S100757_RS18720	S100757_RS15830	-	S100757_RS01535	-	S100757_RS01525
Bacillus subtilis	SRCM100761	NZ_CP021889.1	S100761_RS18720	S100761_RS15830	-	S100761_RS01535	-	S100761_RS01525
Bacillus subtilis	SRCM100333	NZ_CP021892.1	S100333_RS19330	S100333_RS16130	-	S100333_RS01530	-	S100333_RS01520
Bacillus subtilis	SRCM101392	NZ_CP021921.1	S101392_RS19010	S101392_RS16180	-	S101392_RS01650	-	S101392_RS01640
Bacillus subtilis	ge28	NZ_CP021903.1	CD007_RS18540	CD007_RS15685	-	CD007_RS01505	-	CD007_RS01495
Bacillus subtilis	SX01705	NZ_CP022287.1	BSSX_RS18565	BSSX_RS15675	-	BSSX_RS01505	-	BSSX_RS01495
Bacillus subtilis	DKU_NT_02	NZ_CP022890.1	CJZ70_RS01650	CJZ70_RS04790	-	CJZ70_RS18710	-	CJZ70_RS18720
Bacillus subtilis	DKU_NT_03	NZ_CP022891.1	CJZ71_RS09765	CJZ71_RS06335	-	CJZ71_RS13695	-	CJZ71_RS13685
Bacillus subtilis	TLO3	NZ_CP023257.1	CLD04_RS19005	CLD04_RS16140	-	CLD04_RS01860	-	CLD04_RS01850
Bacillus subtilis	ATR2	NZ_CP018133.1	BMJ37_RS17795	BMJ37_RS14990	-	BMJ37_RS18470	-	BMJ37_RS18455
Bacillus subtilis	BJ3-2	NZ_CP025941.1	C0W65_RS06865	C0W65_RS03905	-	C0W65_RS11295	-	C0W65_RS11285
Bacillus subtilis	ATCC 21228	NZ_CP020023.1	BS21228_RS09735	BS21228_RS06200	-	BS21228_RS13760	-	BS21228_RS13750
Bacillus subtilis	KCTC 13429	NZ_CP029465.1	DKG76_RS19340	DKG76_RS16440	-	DKG76_RS01510	-	DKG76_RS01500
Bacillus subtilis	50-1	NZ_CP020915.1	B9L64_RS10810	B9L64_RS07985	-	B9L64_RS14765	-	B9L64_RS14755
Bacillus subtilis	NRS 231	NZ_CP010434.1	SD85_RS18270	SD85_RS15415	-	SD85_RS01465	-	SD85_RS01455
Bacillus subtilis	D12-5	NZ_CP014858.1	KHRBS_RS02415	KHRBS_RS05240	-	KHRBS_RS20150	-	KHRBS_RS20160
Bacillus thermoamylovorans	SSBM	NZ_CP023704.1	-	CQJ30_RS02595	-	CQJ30_RS18285	-	CQJ30_RS11635
Bacillus thuringiensis	97-27	NC_005957.1	BT9727_2194	BT9727_0251	BT9727_0252	BT9727_0252	BT9727_0254	BT9727_0254
Bacillus thuringiensis	YBT-1518	NC_022873.1	-	YBT1518_RS01870	YBT1518_RS01875	YBT1518_RS01875	YBT1518_RS01885	YBT1518_RS01885
Bacillus thuringiensis	Al Hakam	NC_008600.1	-	BALH_RS01735	BALH_RS01740	BALH_RS01740	BALH_RS01750	BALH_RS01750
Bacillus thuringiensis	BMB171	NC_014171.1	-	BMB171_RS01595	BMB171_RS01600	BMB171_RS01600	BMB171_RS01610	BMB171_RS01610
Bacillus thuringiensis	YBT-020	NC_017200.1	-	YBT020_RS07945	-	YBT020_RS22520	-	YBT020_RS01660
Bacillus thuringiensis	CT-43	NC_017208.1	-	CT43_RS06965	-	CT43_RS32940	-	CT43_RS01450
Bacillus thuringiensis	HD-771	NC_018500.1	-	BTG_RS13365	-	BTG_RS25895	-	BTG_RS19075
Bacillus thuringiensis	HD-789	NC_018508.1	-	BTF1_RS27330	BTF1_RS27335	BTF1_RS27335	BTF1_RS27345	BTF1_RS27345
Bacillus thuringiensis	MC28	NC_018693.1	-	MC28_RS25005	MC28_RS25010	MC28_RS25010	MC28_RS25020	MC28_RS25020
Bacillus thuringiensis	Bt407	NC_018877.1	-	BTB_RS07040	-	BTB_RS33870	-	BTB_RS01510
Bacillus thuringiensis	HD73	NC_020238.1	-	HD73_RS01815	HD73_RS01820	HD73_RS01820	HD73_RS01830	HD73_RS01830
Bacillus thuringiensis	IS5056	NC_020376.1	-	H175_RS06955	-	H175_RS35345	-	H175_RS01450
Bacillus thuringiensis	YBT-1520	NZ_CP004858.1	-	YBT1520_RS02095	YBT1520_RS02100	YBT1520_RS02100	YBT1520_RS02110	YBT1520_RS02110
Bacillus thuringiensis	HD-1	NZ_CP004870.1	-	BTK_RS01940	BTK_RS01945	BTK_RS01945	BTK_RS01955	BTK_RS01955
Bacillus thuringiensis	YBT-1520	NZ_CP007607.1	-	DF16_RS00035	DF16_RS00030	DF16_RS00025	DF16_RS00020	DF16_RS00015
Bacillus thuringiensis	HD-29	NZ_CP010089.1	-	BT4G5_RS01330	BT4G5_RS01335	BT4G5_RS01335	BT4G5_RS01345	BT4G5_RS01345
Bacillus thuringiensis	HD1011	NZ_CP009335.1	BF38_RS21725	BF38_RS10850	BF38_RS10855	BF38_RS10855	BF38_RS10865	BF38_RS10865
Bacillus thuringiensis	HD571	NZ_CP009600.1	-	BF32_RS17715	BF32_RS17720	BF32_RS17720	BF32_RS17730	BF32_RS17730
Bacillus thuringiensis	HD682	NZ_CP009720.1	-	BF36_RS23890	BF36_RS23885	BF36_RS23880	BF36_RS23875	BF36_RS23870
Bacillus thuringiensis	97-27	NZ_CP010088.1	BG06_RS01200	BG06_RS11445	BG06_RS11440	BG06_RS11435	BG06_RS11430	BG06_RS11425
Bacillus thuringiensis	HD1002	NZ_CP009351.1	-	AS86_RS23850	AS86_RS23845	AS86_RS23840	AS86_RS23835	AS86_RS23830
Bacillus thuringiensis	BGSC 4AA1	NZ_CP010577.1	-	SD98_RS01500	SD98_RS01505	SD98_RS01505	SD98_RS01515	SD98_RS01515
Bacillus thuringiensis	YC-10	NZ_CP011349.1	-	XI92_RS12645	XI92_RS12640	XI92_RS12635	XI92_RS12630	XI92_RS12625
Bacillus thuringiensis	HS18-1	NZ_CP012099.1	-	AC241_RS07020	-	AC241_RS22455	-	AC241_RS01550
Bacillus thuringiensis	HD521	NZ_CP010106.1	-	NF53_RS01495	NF53_RS01500	NF53_RS01500	NF53_RS01510	NF53_RS01510
Bacillus thuringiensis	YWC2-8	NZ_CP013055.1	-	AQ980_RS28905	AQ980_RS28900	AQ980_RS28895	AQ980_RS28890	AQ980_RS28885
Bacillus thuringiensis	CTC	NZ_CP013274.1	-	ATN06_RS07220	-	ATN06_RS23040	-	ATN06_RS01660
Bacillus thuringiensis	Pasteur Institute Standard strain	NZ_AP014864.1	-	KNN_RS28760	KNN_RS28755	KNN_RS28750	KNN_RS28745	KNN_RS28740
Bacillus thuringiensis	Bt185	NZ_CP014282.1	-	AXW78_RS01570	AXW78_RS01575	AXW78_RS01575	AXW78_RS01585	AXW78_RS01585
Bacillus thuringiensis	HD12	NZ_CP014847.1	-	A3L20_RS07450	-	A3L20_RS23685	-	A3L20_RS01495
Bacillus thuringiensis	Bc601	NZ_CP015150.1	-	BtBc_RS01885	BtBc_RS01890	BtBc_RS01890	BtBc_RS01900	BtBc_RS01900
Bacillus thuringiensis	BGSC 4C1	NZ_CP015176.1	-	Bt4C1_RS01445	Bt4C1_RS01450	Bt4C1_RS01450	Bt4C1_RS01460	Bt4C1_RS01460
Bacillus thuringiensis	MYBT18246	NZ_CP015350.1	-	BT246_RS07835	-	BT246_RS36485	-	BT246_RS01540
Bacillus thuringiensis	KNU-07	NZ_CP016588.1	-	BCM43_RS00175	BCM43_RS00170	BCM43_RS00165	BCM43_RS00160	BCM43_RS00155
Bacillus thuringiensis	Bt18247	NZ_CP015250.1	-	BTI247_RS08175	-	BTI247_RS23525	-	BTI247_RS01520
Bacillus thuringiensis	L-7601	NZ_CP020002.1	-	B4918_RS07320	-	B4918_RS25050	-	B4918_RS01575
Bacillus thuringiensis	ATCC 10792	NZ_CP021061.1	-	CAB88_RS06560	-	CAB88_RS23335	-	CAB88_RS00685
Bacillus thuringiensis	SCG04-02	NZ_CP017577.1	-	BJG91_RS25975	BJG91_RS25980	BJG91_RS25980	BJG91_RS25990	BJG91_RS25990

Bacillus thuringiensis	YGd22-03	NZ_CP019230.1	-	BVH75_RS27660	BVH75_RS27665	BVH75_RS27665	BVH75_RS27675	BVH75_RS27675
Bacillus thuringiensis	BM-BT15426	NZ_CP020723.1	-	B7P25_RS01725	B7P25_RS01730	B7P25_RS01730	B7P25_RS01740	B7P25_RS01740
Bacillus thuringiensis	c25	NZ_CP022345.1	-	CCX84_RS24420	CCX84_RS24415	CCX84_RS24410	CCX84_RS24405	CCX84_RS24400
Bacillus thuringiensis	XL6	NZ_CP013000.1	-	BTXL6_RS16670	-	BTXL6_RS05475	-	BTXL6_RS06155
Bacillus toyonensis	BCT-7112	NC_022781.1	-	BTOYO_RS15065	BTOYO_RS15070	BTOYO_RS15070	BTOYO_RS15080	BTOYO_RS15080
Bacillus vallismortis	NBIF-001	NZ_CP020893.1	B9C48_RS17280	B9C48_RS14415	B9C48_RS04040	B9C48_RS04035	B9C48_RS04055	B9C48_RS04050
Bacillus velezensis	FZB42	NC_009725.1	RBAM_RS16820	RBAM_RS14095	-	RBAM_RS17495	-	RBAM_RS17480
Bacillus velezensis	CAU B946	NC_016784.1	BACAU_RS17605	BACAU_RS14825	-	-	-	BACAU_RS11120
Bacillus velezensis	YAU B9601-Y2	NC_017061.1	BANAU_RS18615	BANAU_RS15850	BANAU_RS04020	BANAU_RS04015	BANAU_RS04035	BANAU_RS04030
Bacillus velezensis	AS43.3	NC_019842.1	B938_RS17095	B938_RS14380	-	-	-	B938_RS02835
Bacillus velezensis	UCMB5036	NC_020410.1	BAM5036_RS16805	BAM5036_RS14030	-	BAM5036_RS18230	-	BAM5036_RS10550
Bacillus velezensis	UCMB-5033	NC_022075.1	RBAU_RS17785	RBAU_RS15050	-	-	-	RBAU_RS11515
Bacillus velezensis	UCMB5113	NC_022081.1	BASU_RS17020	BASU_RS14345	-	-	-	BASU_RS10780
Bacillus velezensis	NAU-B3	NC_022530.1	BAPNAU_RS18325	BAPNAU_RS15490	BAPNAU_RS04050	BAPNAU_RS04045	BAPNAU_RS04065	BAPNAU_RS04060
Bacillus velezensis	TrigoCor1448	NZ_CP007244.1	AJ82_RS16935	AJ82_RS14215	-	AJ82_RS17625	-	AJ82_RS17610
Bacillus velezensis	SQR9	NZ_CP006890.1	V529_RS17740	V529_RS15045	V529_RS03830	V529_RS03825	V529_RS03845	V529_RS03840
Bacillus velezensis	JS25R	NZ_CP009679.1	NG74_RS17135	NG74_RS14305	NG74_RS03960	NG74_RS03955	NG74_RS03975	NG74_RS03970
Bacillus velezensis	NJN-6	NZ_CP007165.1	AW02_RS17605	AW02_RS14880	-	-	-	AW02_RS11470
Bacillus velezensis	JJ-D34	NZ_CP011346.1	AAV29_RS18140	AAV29_RS15410	-	-	-	AAV29_RS11665
Bacillus velezensis	YJ11-1-4	NZ_CP011347.1	AAV30_RS02165	AAV30_RS04900	AAV30_RS15250	AAV30_RS15250	AAV30_RS15235	AAV30_RS15235
Bacillus velezensis	G341	NZ_CP011686.1	ABH13_RS17410	ABH13_RS14470	-	-	-	ABH13_RS11000
Bacillus velezensis	B25	NZ_LN999829.1	BAMMD1_RS16345	BAMMD1_RS13620	-	-	-	BAMMD1_RS10205
Bacillus velezensis	CC09	NZ_CP015443.1	A1D33_RS11355	A1D33_RS14170	-	A1D33_RS09930	-	A1D33_RS17650
Bacillus velezensis	S3-1	NZ_CP016371.1	A5891_RS16880	A5891_RS14070	A5891_RS03960	A5891_RS03955	A5891_RS03975	A5891_RS03970
Bacillus velezensis	LS69	NZ_CP015911.1	A8142_RS16750	A8142_RS13940	A8142_RS03820	A8142_RS03815	A8142_RS03835	A8142_RS03830
Bacillus velezensis	M75	NZ_CP016395.1	BBJ33_RS17470	BBJ33_RS14725	-	-	-	BBJ33_RS11240
Bacillus velezensis	D2-2	NZ_CP014990.2	A2I97_RS17350	A2I97_RS14540	-	-	-	A2I97_RS10910
Bacillus velezensis	SYBC H47	NZ_CP017747.1	BI197_RS16595	BI197_RS13825	-	-	-	BI197_RS04650
Bacillus velezensis	9912D	NZ_CP017775.1	BK055_RS18710	BK055_RS15805	-	BK055_RS19370	-	BK055_RS19355
Bacillus velezensis	GH1-13	NZ_CP019040.1	BVH55_RS18410	BVH55_RS15595	-	BVH55_RS19895	-	BVH55_RS12010
Bacillus velezensis	sx01604	NZ_CP018007.1	BLL65_RS01810	BLL65_RS04685	BLL65_RS15010	BLL65_RS15010	BLL65_RS14995	BLL65_RS14995
Bacillus velezensis	JTYP2	NZ_CP020375.1	BAJT_RS17275	BAJT_RS14410	BAJT_RS04040	BAJT_RS04035	BAJT_RS04055	BAJT_RS04050
Bacillus velezensis	9D-6	NZ_CP020805.1	B7941_RS00425	B7941_RS17380	-	-	-	B7941_RS05465
Bacillus velezensis	CBMB205	NZ_CP011937.1	AAV34_RS02185	AAV34_RS05050	AAV34_RS15440	AAV34_RS15440	AAV34_RS15425	AAV34_RS15425
Bacillus velezensis	ZL918	NZ_CP021338.1	B9C70_RS17470	B9C70_RS14665	-	-	-	B9C70_RS11065
Bacillus velezensis	GQJK49	NZ_CP021495.1	BAGQ_RS17415	BAGQ_RS14535	BAGQ_RS04055	BAGQ_RS04050	BAGQ_RS04070	BAGQ_RS04065
Bacillus velezensis	T20E-257	NZ_CP021976.1	CEG11_RS17340	CEG11_RS14555	-	-	-	CEG11_RS11025
Bacillus velezensis	157	NZ_CP022341.1	CFN60_RS17665	CFN60_RS14765	CFN60_RS04095	CFN60_RS04090	CFN60_RS04110	CFN60_RS04105
Bacillus velezensis	TB1501	NZ_CP022531.1	CG798_RS03315	CG798_RS00520	-	CG798_RS04010	-	CG798_RS03995
Bacillus velezensis	NJAU-Z9	NZ_CP022556.1	CF389_RS16555	CF389_RS00215	-	-	-	CF389_RS03750
Bacillus velezensis	SCDB 291	NZ_CP022654.2	CHN56_RS13060	CHN56_RS15885	-	-	-	CHN56_RS08100
Bacillus velezensis	SCGB 1	NZ_CP023320.1	CLI97_RS08475	CLI97_RS05645	-	-	-	CLI97_RS13430
Bacillus velezensis	SCGB 574	NZ_CP023431.1	CLI98_RS07350	CLI98_RS04445	CLI98_RS13695	CLI98_RS13690	CLI98_RS13710	CLI98_RS13705
Bacillus velezensis	LABIM40	NZ_CP023748.1	CQJ38_RS17545	CQJ38_RS14710	-	CQJ38_RS19125	-	CQJ38_RS02745
Bacillus velezensis	L-1	NZ_CP023859.1	CRH11_RS02920	CRH11_RS00130	-	CRH11_RS03620	-	CRH11_RS03605
Bacillus velezensis	NKG-1	NZ_CP024203.1	CS376_RS18985	CS376_RS16155	-	CS376_RS19710	-	CS376_RS19695
Bacillus velezensis	TJ02	NZ_CP024797.1	CUB85_RS18200	CUB85_RS15405	-	CUB85_RS18900	-	CUB85_RS18885
Bacillus velezensis	CN026	NZ_CP024897.1	CVD07_RS17480	CVD07_RS14670	-	CVD07_RS18150	-	CVD07_RS18135
Bacillus velezensis	Lzh-a42	NZ_CP025308.1	CXP43_RS19150	CXP43_RS16305	CXP43_RS04105	CXP43_RS04100	CXP43_RS04120	CXP43_RS04115
Bacillus velezensis	CMT-6	NZ_CP025341.1	CXR48_RS17265	CXR48_RS14470	-	-	-	CXR48_RS10960
Bacillus velezensis	GFP-2	NZ_CP021011.1	B6257_RS07730	B6257_RS04990	-	B6257_RS08380	-	B6257_RS08360
Bacillus velezensis	10075	NZ_CP025939.1	C0W57_RS15935	C0W57_RS13125	-	C0W57_RS16605	-	C0W57_RS16590
Bacillus velezensis	DKU_NT_04	NZ_CP026533.1	C3438_RS07275	C3438_RS04415	-	C3438_RS07940	-	C3438_RS07925
Bacillus velezensis	CGMCC 11640	NZ_CP026610.1	C3Z10_RS19395	C3Z10_RS16505	-	C3Z10_RS20850	-	C3Z10_RS12525
Bacillus velezensis	DR-08	NZ_CP028437.1	DA376_RS17515	DA376_RS14615	DA376_RS04160	DA376_RS04155	DA376_RS04175	DA376_RS04170
Bacillus velezensis	J7-1	NZ_CP028440.1	DA378_RS13755	DA378_RS16565	-	-	-	DA378_RS00590
Bacillus velezensis	8-2	NZ_CP028439.1	DA377_RS03225	DA377_RS06035	-	-	-	DA377_RS09560
Bacillus velezensis	131-4	NZ_CP028441.1	DA379_RS15110	DA379_RS12310	-	-	-	DA379_RS08780
Bacillus velezensis	GYL4	NZ_CP020874.1	B9C53_RS12400	B9C53_RS15185	B9C53_RS06060	B9C53_RS06060	B9C53_RS06045	B9C53_RS06045
Bacillus velezensis	QST713	NZ_CP025079.1	BVQ_RS19140	BVQ_RS16220	-	BVQ_RS20620	-	BVQ_RS12135
Bacillus velezensis	LDO2	NZ_CP029034.1	DDE72_RS18940	DDE72_RS02230	DDE72_RS12690	DDE72_RS12690	DDE72_RS12675	DDE72_RS12675
Bacillus velezensis	BS-37	NZ_CP023414.1	CMR26_RS19165	CMR26_RS01865	-	-	-	CMR26_RS05460
Bacillus velezensis	W1	NZ_CP028375.1	C9888_RS19125	C9888_RS16275	C9888_RS04070	C9888_RS04065	C9888_RS04085	C9888_RS04080
Bacillus weihaiensis	Alg07	NZ_CP016020.1	-	-	-	A9C19_RS01570	-	A9C19_RS07410
Bacillus xiamenensis	VV3	NZ_CP017786.1	BK049_RS10410	BK049_RS13040	-	BK049_RS08835	-	BK049_RS16710
