# Supplementary material for: Loss of Bacitracin Resistance Due to a Large Genomic Deletion among Bacillus anthracis Strains
Source: mSystems. 2018 Oct 30;3(5):e00182-18. doi: 10.1128/mSystems.00182-18 (PMC6208641; doi:10.1128/mSystems.00182-18)
Supplement: TABLE S4 [file sys005182281st4.pdf]

Table S4. Canonical SNPs in B. anthracis Zambian isolates.

Strain	A.Br.001	A.Br.002	A.Br.003	A.Br.004	A.Br.006	A.Br.007	A.Br.008	A.Br.009	B.Br.001	B.Br.002	B.Br.003	B.Br.004	A/B.Br.001
NL2016_1	T	A	A	T	A	T	T	A	T	G	G	T	A
NL2016_2	T	A	A	T	A	T	T	A	T	G	G	T	A
NL2016_3	T	A	A	T	A	T	T	A	T	G	G	T	A
CZC5	T	A	A	T	A	T	T	A	T	G	G	T	A
Han19	T	A	A	T	A	T	T	A	T	G	G	T	A
Mongu1	T	A	A	T	A	T	T	A	T	G	G	T	A
Mongu37	T	A	A	T	A	T	T	A	T	G	G	T	A
Human29	T	A	A	T	A	T	T	A	T	G	G	T	A
Mox4	T	A	A	T	A	T	T	A	T	G	G	T	A
LZprevious	T	A	A	T	A	T	T	A	T	G	G	T	A
LZ2013T	T	A	A	T	A	T	T	A	T	G	G	T	A
LZ88	T	A	A	T	A	T	T	A	T	G	G	T	A
LZ3	T	A	A	T	A	T	T	A	T	G	G	T	A
LZ131001	T	A	A	T	A	T	T	A	T	G	G	T	A
K0123[a]	T	G	A	T	A	T	T	A	T	G	G	T	A

a The strain described as type strain of A.Br.005/006 group in Van Ert et al, 2007.
